# Accurate gene consensus at low nanopore coverage

**DOI:** 10.1093/gigascience/giac102

**Published:** 2022-11-09

**Authors:** Rocío Espada, Nikola Zarevski, Adèle Dramé-Maigné, Yannick Rondelez

**Affiliations:** Gulliver Lab, ESPCI Paris, PSL University, CNRS, 75005 Paris, France; Gulliver Lab, ESPCI Paris, PSL University, CNRS, 75005 Paris, France; Gulliver Lab, ESPCI Paris, PSL University, CNRS, 75005 Paris, France; Gulliver Lab, ESPCI Paris, PSL University, CNRS, 75005 Paris, France

**Keywords:** nanopore sequencing, consensus sequence, low coverage, gene library

## Abstract

**Background:**

Nanopore technologies allow high-throughput sequencing of long strands of DNA at the cost of a relatively large error rate. This limits its use in the reading of amplicon libraries in which there are only a few mutations per variant and therefore they are easily confused with the sequencing noise. Consensus calling strategies reduce the error but sacrifice part of the throughput on reading typically 30 to 100 times each member of the library.

**Findings:**

In this work, we introduce SINGLe (SNPs In Nanopore reads of Gene Libraries), an error correction method to reduce the noise in nanopore reads of amplicons containing point variations. SINGLe exploits that in an amplicon library, all reads are very similar to a wild-type sequence from which it is possible to experimentally characterize the position-specific systematic sequencing error pattern. Then, it uses this information to reweight the confidence given to nucleotides that do not match the wild-type in individual variant reads and incorporates it on the consensus calculation.

**Conclusions:**

We tested SINGLe in a mutagenic library of the KlenTaq polymerase gene, where the true mutation rate was below the sequencing noise. We observed that contrary to other methods, SINGLe compensates for the systematic errors made by the basecallers. Consequently, SINGLe converges to the true sequence using as little as 5 reads per variant, fewer than the other available methods.

## Findings

### Background

Nanopore is a powerful technology for high-throughput DNA sequencing, currently commercialized by Oxford Nanopore Technologies [1]. It provides sequence base calls reconstructed from conductivity records during the translocation of a single DNA molecule through a protein pore. This approach offers portability and real-time sequencing, using simple experimental protocols, for a relatively low cost. A minION device can read DNA strands of various lengths, from PCR products up to megabase genomic fragments, and current versions return at least 5 × 10^9^ bases in one run. Therefore, it is an attractive device for sequencing libraries of amplicons that are too long for other next-generation sequencing technologies. There is an increasing interest in using next-generation sequencing technologies for analyzing gene libraries that are highly diverse but have low variability (i.e., containing many different sequences differing from each other by only a few point mutations and for which a reference is available). This is the case in directed evolution experiments, in which the genetic libraries typically originate from a single ancestral sequence (the wild-type) that has been submitted to limited randomization, for example, using error-prone PCR (epPCR) [2]. Another application is the detection of structural variants in cancer cells [3].

Unfortunately, nanopore's relatively high error rate (≈6–15%) prevents the accurate detection of point genetic variation directly from individual reads [4–6], and specific tools are not yet available. Previous work aiming at high-quality sequencing from nanopore data has concentrated on polishing tools such as Nanopolish [7] or Racon [8] combined with Medaka [9]. These approaches start from a draft assembly and use the coverage depth to compute an averaged consensus at each position, via various computational approaches. Nanopolish reports an accuracy over 99.5%, for a 29× sequencing coverage, and Medaka 98% in detection of single-nucleotide polymorphisms (SNPs) with a coverage of 100×. While these tools primarily apply to genome assembly, a number of experimental protocols were developed in order to apply these pipelines in the specific case of amplicon library sequencing. These strategies aim to read and associate several replicates of the same molecule. This has been achieved by creating sequence concatenates using rolling circular amplification [10], which retrieved an accuracy of 99.5% for coverage of 150×, and via gene barcoding prior to amplification [11] with a reported accuracy over 99.9% for 25× coverage. Inconveniently, these methods reduce the number of different variants that can be studied, because a part of the sequencing throughput is invested in reading each sequence multiple times.

In this work, we introduce SINGLe (SNPs In Nanopore reads of Gene Libraries), a method that improves detection of single mutations via consensus calling in reads of libraries for which a reference sequence is known. SINGLe is first trained on a set of reads of the reference by a nanopore sequencer, and then it is applied to the reads of the actual library to correct their quality scores (Qscore). Finally, these values can be used in the consensus calling of individual variants.

Here, we applied SINGLe to the gene of KlenTaq, a truncated variant of the well-known Taq polymerase, of approximately 1.7 kb in length. We first tested it on a small set of 7 known mutants containing 2 to 9 point mutations and later a larger library of approximately 1,200 variants. SINGLe reduced the sequencing noise, allowing a better identification of true point mutations. Therefore, as few as 5 to 7 reads return a trustable consensus sequence, outperforming the state-of-the-art tools for consensus computation of nanopore sequencing, Medaka and Nanopolish. This translates into a more efficient exploitation of the sequencing throughput.

### SINGLe method

**Figure 1:**
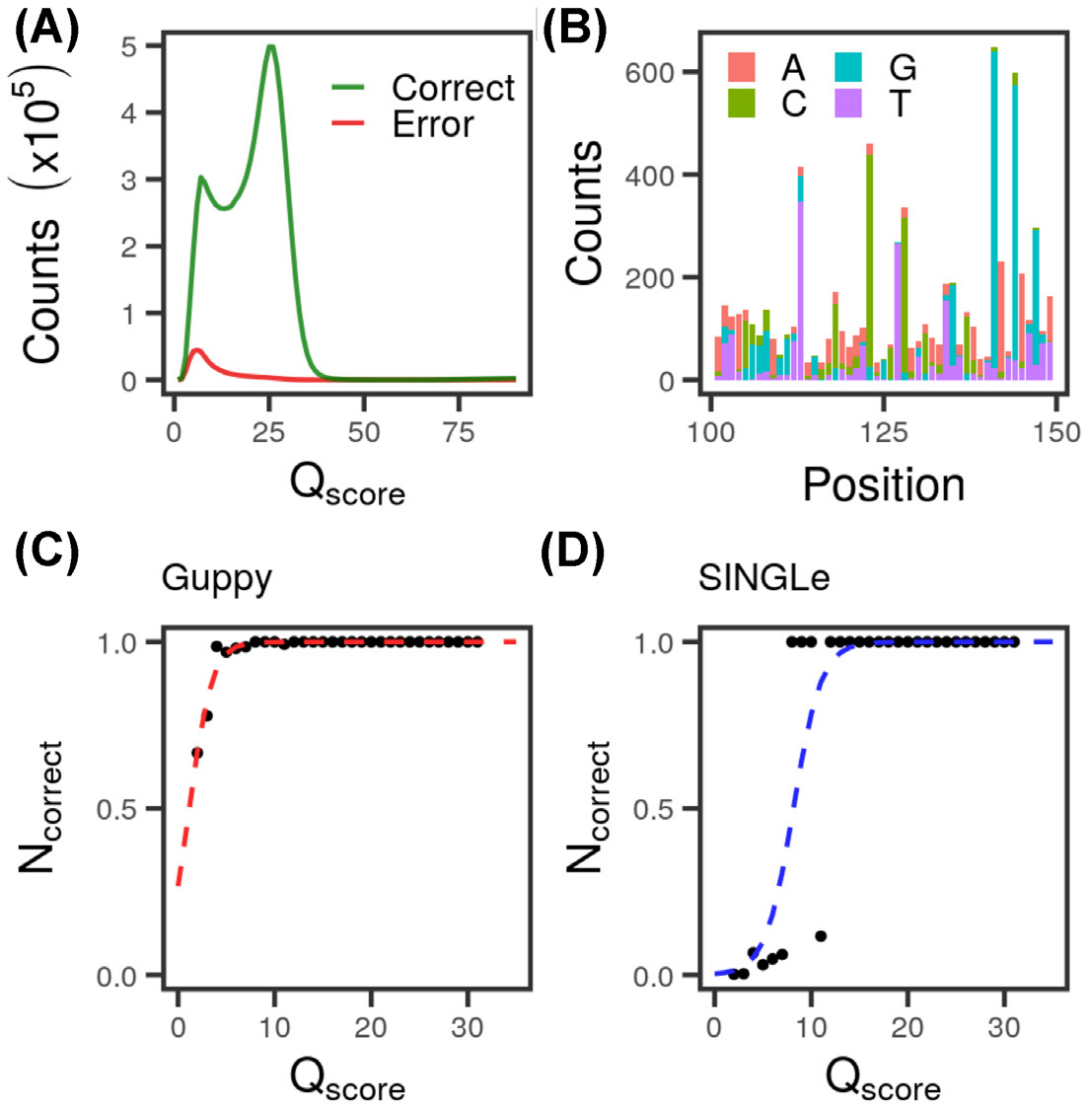
(A) Distribution of Qscore returned by Guppy basecaller for each nucleotide on the sequencing of the wild-type KlenTaq gene, classified as correct reads (green) and errors (red). (B) Nanopore sequencing errors per position on reads of the wild-type KlenTaq gene. Colors indicate the nucleotide reported. Wild-type (correct) nucleotides are not plotted. Only positions 100 to 150 are shown here. An equivalent plot for all positions is available in Supplementary Fig. S2. (C) Example of logistic regression over reads of a known wild-type sequence. Black dots are the proportion of correct nucleotides with a given Qscore in one position and in comparison to one possible error. Dashed red line is the logistic regression performed. (D) Same plots as C after data were weighted according to the prior probabilities. Blue line is *p_SINGLe_*.

We used a nanopore sequencer to read 5,847 strands of the wild-type KlenTaq gene (length 1,662 nucleotides), for which we have a ground-truth sequence obtained by Sanger sequencing (Supplementary Sequence S1). In this data set, we can confidently attribute mismatches between the read sequences and the known wild-type as sequencing errors and matches as correct reads. In Fig. 1A (and a normalized version in Supplementary Fig. S1), we plotted the distribution of correct/error nucleotides according to the Qscore returned by Oxford nanopore's basecaller, Guppy. The Qscore assigned to each nucleotide tends to be low when a wrong nucleotide is assigned, as expected. Notice that the inverse is not true: some low Qscores correspond to correctly basecalled bases, so both distributions overlap. Thus, a simple classification based on the Qscores is not possible to distinguish signal from noise. We used this same dataset to plot the counts of errors by position and nucleotide (Fig. 1B and Supplementary Fig. S2). The errors are not homogeneously distributed, and they are more frequent in some positions of the DNA sequence than others. Previous work has also shown that nanopore sequencing produces some systematic errors [4, 12, 13], even for high-accuracy basecalling. These 2 observations inspired SINGLe as a procedure to reduce the nonrandom part of the sequencing errors, using the information contained in the Qscore.

The first step in SINGLe is to fit the probability of being a correct read on these wild-type reads. We counted the errors in each position and nucleotide and plotted it against the Qscore (Fig. 1C). We fitted this relation by a logistic regression using a binomial model, which provided a classifier able to convert the reported Qscore to the probability that this read is indeed correct. Nevertheless, as it was computed over wild-type reads that do not contain true mutations, the model was heavily biased against mutations. This is not representative of the actual proportion of errors/correct reads present in the mismatches of a set of mutants. To adapt the classifier, an *a priori* expectation of mutations (*p_prior_*_−_*_right_*) is needed, which must come from independent information. In the presented case, the variant sequences originated from an epPCR, for which we possessed an estimate of the mutation rate *m_n→n_*_’_ given by the manufacturer [14]. We computed the *a priori* probability of observing nucleotide *n*′ as *p_prior−right_* (*n*→*n′*) = *m_n→n′_* − <*m*>/[Σ_*n*_ counts_reference(*n*) Σ_*n*’_  *m_n→n_*_′_], where <*m*> is the mean number of mutations expected in the library (reported by the epPCR kit manufacturer), and counts_reference(*n*) is how many times the nucleotide *n* is present in the wild-type DNA strand. We also set the *a priori* expectation of an observed mismatch to be a sequencing error (*p^p,n^_prior_*_−_*_error_*) to the sequencing error rate at that position in the wild-type set, independently of the Qscore. Using these values, we computed for each strand, position (*p*), and nucleotide (*n*) the probability of observing a mutation with Qscore = Q as P*^p,n^*_mutation_ (Q) = [counts*^p,n^*(Q)/Σ_Q’_ counts*^p,n^*(Q′)] − *p^p,n^_prior_*_−error_, where counts*^p,n^* is the number of times the nucleotide *n* appears in position *n* across all the reads. Similarly, we computed the probability of observing a wild-type nucleotide with Qscore = Q as P*^p,n^*_wild-type_(Q) = [counts*^p,wild-type^*(Q)/Σ_Q’_ counts*^p,wild-type^*(Q′)] − *p_prior_*_−right_(wild-type→*n*). Finally, we normalized N*^p,n^*_correct_ (Q) = P*^p,n^*_wild-type_/[P*^p,n^*_wild-type_(Q) + P*^p,n^*_mutation_(Q)]. This process shifts the logistic regression toward the higher Qscore, allowing the classifier to accept a number of observed mismatches consistent with the prior expectation (Fig. 1D). The fits were done independently for each position and possible mismatched nucleotides. To include deletions in this analysis (which do not have a Qscore assigned by the basecaller), we fixed their confidence value as the minimum of the Qscore of their direct nearest neighbors in the nucleotide sequence. This decision was inspired by the observation that the Qscore is correlated between consecutive nucleotides (Supplementary Fig. S3). Insertions were ignored as very few are expected (<1%) and it is not possible to obtain enough reads to fit all possible insertions. In applications where SINGLe is used to compute a high-quality consensus, the original score of the inserted bases can be carried over. We also separated the fits for forward and reverse strands as the error rate per position is different in each case (see “Are reverse and forward reads different in nanopore sequencing?” in the supplementary material). All together, we obtained 13,296 = 1,662 × 4 × 2 regressions, one for each position of the gene (1,662 bp), for each non-wild-type nucleotide or deletion (4 possibilities in total for each position) and for the forward and reverse sense of sequencing. Please refer to the supplementary material for a brief discussion on “How many nanopore reads are needed to fit SINGLe?”.

The regressions were then used to rescore the mismatches in the mutant library: for each non-wild-type nucleotide aligned to the reference, we evaluated the Qscore reported by Guppy in the SINGLe fit obtained for that particular position and nucleotide and defined this value (*p_SINGLe_*) as its probability of being correct *p_right_*. For nucleotides read as wild-type, their Qscore is directly transformed into a *p_right_* according to the Q values reported by Guppy: *p_Guppy_ =*1 – 10*^−^*^Qscore/10^. Finally, to compute a consensus sequence, we performed a weighted count of each nucleotide (and deletion) in each position (by summing *p_SINGLe_* values instead of ones) and defined the consensus nucleotide as the one with the highest value. Homopolymer regions were sorted so that the deletions are always at the 3′ side on the forward strand. To compare, we also computed the variant consensus sequence using *p_Guppy_*instead of *p_SINGLe_* for nucleotides that do not match the wild-type or by unweighted majority vote. In these cases, we did not sort the homopolymer region as it had a detrimental effect (see Supplementary Fig. S4).

### Analysis

We tested SINGLe in a small set composed of 7 variants of KlenTaq (named #1 to #7), which we obtained from independent bacterial clones and barcoded them using the nanopore barcoding kit before sequencing. Their true sequence was obtained by Sanger. Each contains 2 to 9 point mutations (Supplementary Table S1). Variants #1 to #5 only present nucleotide substitutions. Variant #6 has 7 substitutions, 2 of them in consecutive positions, and 1 deletion in a non-homopolymer region. Variant #7 has 5 substitutions and a deletion in a homopolymer (“GG” to “G-”).

#### Signal-to-noise ratio

A straightforward procedure to filter errors is to only trust read nucleotides that have a probability of being correct *p_right_*higher than a threshold. In this section, we compare how this process performs over the 7 KlenTaq variants when using either *p_right_* = *p_Guppy_* or *p_right_* = *p_SINGLe_*.

**Figure 2:**
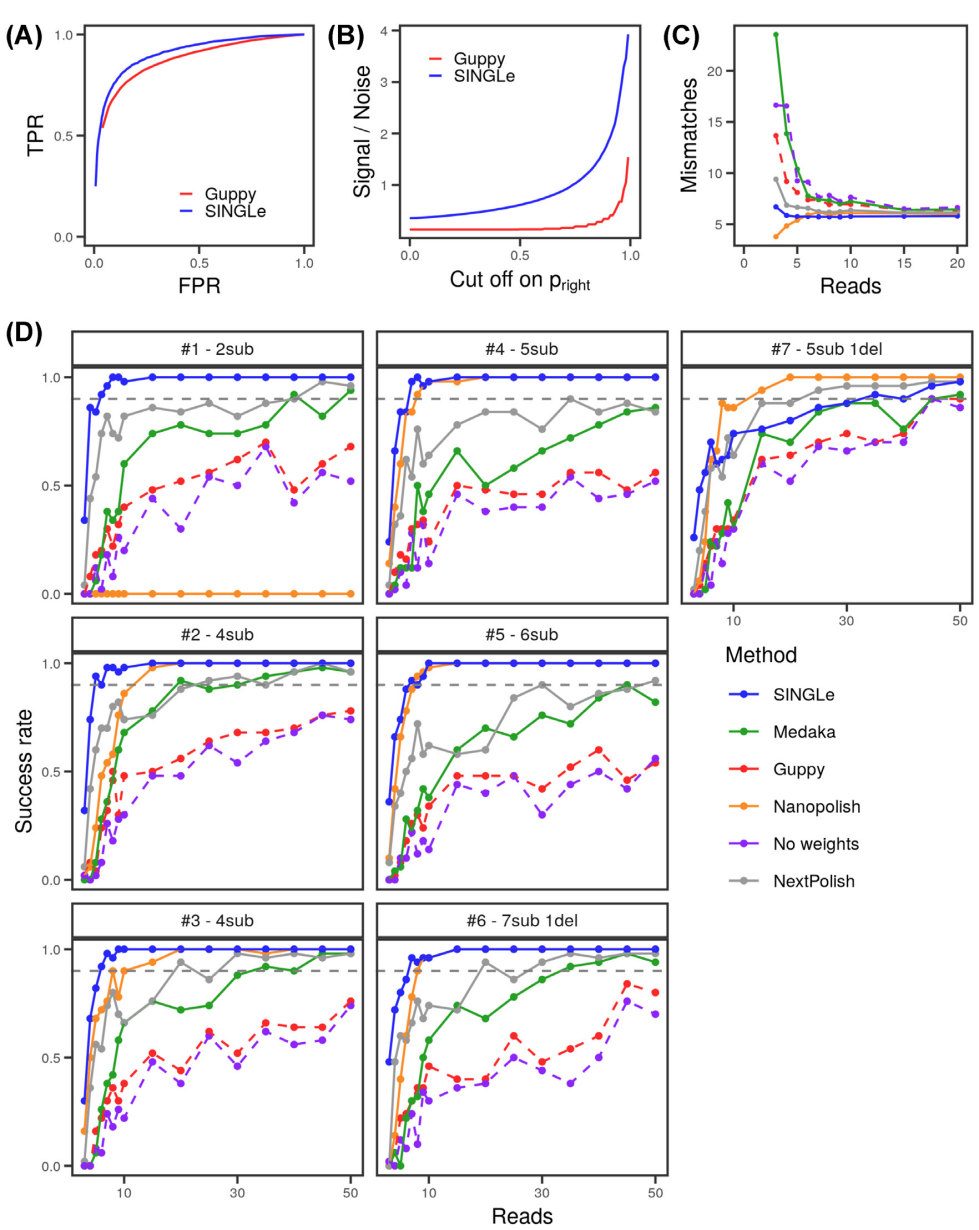
(A) True-positive rate (TPR) vs false-positive rate (FPR) (or ROC curve) for SINGLe (blue) and Guppy (red). (B) Signal-to-noise ratio when classifying mismatches as mutations when they reach *p_right_* indicated on the x-axis, for SINGLe (blue) and Guppy (red). (C) Mean number of mismatches between the consensus of variant #3 (4 substitutions) and the wild-type sequence when using a subset of sequences (x-axis) to compute the consensus. Colors indicate the method used for computing the consensus. (D) Success rate on obtaining the true sequence when computing the consensus with a subset of reads. The size of the subset used is indicated on the x-axis. The success rate is computed over 50 different consensuses on independent subsets of reads. The colors indicate the method used for the consensus calling. Each panel has one of the 7 variants analyzed, and the number of substitutions (sub) and deletions (del) is indicated on top.

We defined *signal* as the number of mismatches to wild-type known to be actual mutations with *p_right_* higher than the threshold (true positives) and *noise* as the number of those mismatches known to be a wild-type nucleotide (false positives). The counts are also weighted by *p_right_* for each nucleotide; that is, instead of summing one for each occurrence, we summed the *p_right_* associated with the nucleotide. Results are shown in Fig. 2A (receiver operating characteristic [ROC] curve), Fig. 2B (signal-to-noise ratio), and Supplementary Fig. S5 (signal-to-noise ratio without weighting counts by *p_right_*). For all thresholds, SINGLe has a higher signal-to-noise ratio (up to 6 times higher, depending on the cutoff), thus facilitating the identification of actual mutations. This remains true when no cutoff is applied (cutoff = 0). Notice that the ratio is different for both methods at cutoff zero because the counts are weighted by *p_right_*.

#### Consensus sequences

We compared the consensus sequences of the 7 KlenTaq variants obtained by SINGLe and by other methods. Given several aligned nanopore reads of the same variant, the consensus can be computed by simple majority counting how many times each nucleotide was read in one position and keeping the most frequent. We call this method “no weights.” The counts can be weighted by *p_Guppy_*, and the nucleotide with the larger weighted count can be chosen. We call this method “Guppy.” Or the counts can be weighted by *p_SINGLe_* (“SINGLe” method). We also computed the consensus using Nanopolish, which works directly on the raw electrical signal instead of the basecalled sequences in combination with hidden markov models (HMM) to detect base variations [7]; Medaka, which counts nucleotides and uses a neural network to compare to a draft assembly and define mutations [9]; and NextPolish, which takes into account the neighbors of the nucleotide [15].

For each method, we computed the consensus independently for each variant using subsets of 3 to 50 sequences drawn randomly from all available reads and repeated 50 times for each subset size. In Fig. 2D, we plotted the success rate on the consensus computation (i.e., how many times the obtained consensus matches exactly the true sequence). For variants #1 to #6, the convergence is faster when using SINGLe weights: perfect consensuses are obtained for more than 90% of attempts starting from 5 to 7 sequences. Nanopolish reaches a 90% of success rate in 8 to 15 reads but fails for variant #1 (it systematically misses mutation G23A). Medaka requires more than 20 reads to reach 90% success, and it does not converge for variants #4 and #5. NextPolish needs between 15 and 50 reads depending on the variant. Finally, using *p_Guppy_* or no weights has a poorer performance, not reaching 90% success for 50 reads for any of the variants #1 to #6. Notice that variant #6 has 2 consecutive mutations, and they are properly detected by SINGLe. Variant #7 has a deletion in a homopolymer, which is a challenging mutation to detect. In this case, SINGLe needs 35 reads to converge to the true sequence, still outperforming Medaka, Guppy, and no weights (they need 45 reads). Only Nanopolish converges faster, with 15 reads.

Fig. 2C shows the total number of mismatches (averaged over the 50 trials) reported by each method for variant #3, according to the number of reads used to compute the consensus. For any set size, SINGLe reports the closest number of true mutations compared to the other methods. In Supplementary Fig. S6, this is analyzed in more detail: we classified the nucleotides in each consensus sequence according to true/false mutations and true/false wild-types and observed that actually SINGLe detects true mutations with the fewest number of reads while keeping the lowest rate of false mutations. Nanopolish has a high rate of false wild-types and underestimates the true mutations. Medaka has a similar behavior and on top adds false mutations. Consensus using Guppy scores or no weight reports a high number of false mutations, and so does NextPolish (although with a lower error rate).

#### Consensus in a large gene library

**Figure 3:**
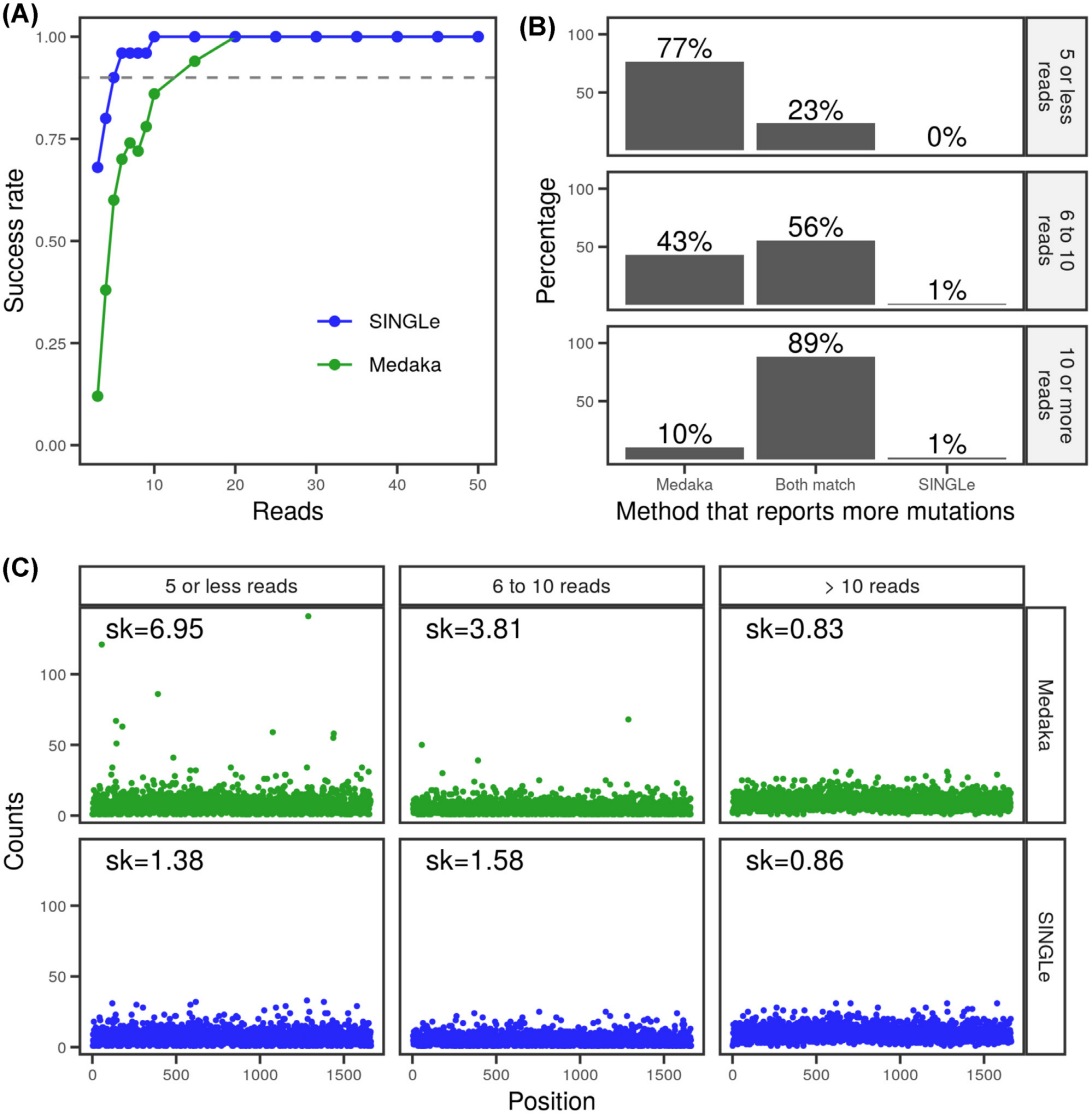
(A) For the most frequent variant in the library, success rate of the consensus computed from subsets of available reads using Medaka (green) or SINGLe (blue). (B) Comparison of the consensus sequences returned by Medaka and SINGLe. We split the results by the number of reads available for each variant (fewer than 5, 6 to 10, and more than 10). The bars in the middle indicate the proportion of variants whose consensus sequence is the same computed by Medaka or by SINGLe. The left bar indicates the proportion of variants whose consensus sequences have more mutations when computed with Medaka than when computed by SINGLe, and the right bar indicates the opposite. (C) Mismatches to wild-type on the consensus computed by Medaka (upper panels) and SINGLe (lower panels) by position and classified according to the number of reads available for the consensus computation (label on top). To quantify the systematicity of the mismatches, we used the skewness (sk) of the distribution of counts.

We also tested SINGLe on a large library of mutants of the KlenTaq gene obtained by epPCR, containing approximately 1,200 variants with a mean mutation rate of 8 mutations/kb. The variants are unknown but associated with a barcode of 36 nucleotides downstream the STOP codon. We sequenced the library using nanopore, grouped the reads according to the barcode, and computed the consensus sequence for the reads associated with each barcode. We first confirmed the consistency of the different consensus methods on this library, with respect to the results obtained in previous sections of this article. We chose the most represented barcode (901 reads) and computed the success rate using Medaka and SINGLe for subsets of reads. As ground truth, we used the consensus computed with all available reads, which is the same for all methods except Nanopolish (Supplementary Table S2). As shown in Fig.   3A, SINGLe returns the correct consensus sequence over 90% of the time when using at least 5 reads, while Medaka needs 15. Similar results were obtained for the other 9 most frequent barcodes using various methods for computing the consensus sequence (Supplementary Table S2 and Supplementary Fig. S7).

We computed consensus sequences using SINGLe or Medaka for all the mutants in our library, provided that the identifying barcode is present at least 4 times in our dataset, and compared the number of mutations reported by both methods (Fig. 3B). Out of the 1,174 variants for which we had at least 10 reads, both methods report the same consensus in 1,039 cases (89%). Among the 610 variants for which we had 6 to 10 reads, we obtained the same consensus sequence using Medaka or SINGLe in only 339 variants (56%), and for the 839 variants with fewer than 5 reads, only 23% of the variants obtained the same consensus with both methods. For the other variants, independently of the number of available reads, Medaka returns more mutations than SINGLe in most cases. This is consistent with the observation in the previous section: Medaka tends to predict more mutations than there actually are. Less than 1% of all computed consensuses have more mutations by SINGLe than by Medaka.

If SINGLe improves the consensus sequences by reducing systematic errors from the basecalling, then the mutations predicted for a randomly mutated gene library should be homogeneously distributed along the sequence. In Fig. 3C, we show that this is the case. When there are more than 10 reads available in each cluster, SINGLe and Medaka predict mutations that are similarly distributed throughout the gene, as reflected by the skewness of the distribution of mutation counts (sk), around 0.8 in both cases. When there are only 6 to 10 reads available, Medaka tends to predict mutations on some preferred spots, increasing sk to 3.8. The effect is even larger for 5 or fewer reads: Medaka shows strong systematic errors (sk around 7), while SINGLe's reaches sk = 1.3. In Supplementary Fig. S8, this same analysis is performed for other consensus calling methods, and they all have a higher sk than SINGLe. Finally, we also compared the bias of the mutations in our library to the one reported by the manufacturer (Supplementary Fig. S9). All methods (except Nanopolish) had a correlation of .94 when sequences with more than 10 reads were used. Only SINGLe reaches this value for the sequences with 4 or 5 reads available.

## Discussion

The relatively high error rate in single-molecule nanopore sequencing limits some applications such as the analysis of libraries containing many different, but genetically similar, sequences. The approach we introduce here, SINGLe, leverages the fact that the sequencing errors in this case are partly systematic, as previously noted [4, 12]. We accumulate many reads from the reference gene to build a sequence-specific error model that locally corrects for the sequencing biases. Applying this procedure on a set of 7 variants of the KlenTaq gene with an average mutation rate of 3 bases/kb, we showed that correcting the confidence values provides a large increase in the signal-to-noise ratio. Consequently, the consensus calling returns 90% of perfect results from typically 5 to 7 reads. This implies a faster convergence than other methods currently used: Nanopolish requires 8 to 15 reads, Medaka needs at least 20 to 50 reads, and NextPolish between 15 and 50 reads to achieve a similar performance. Therefore, with SINGLe, a lower burden on the sequencing throughput is taken to obtain true consensus sequences. Even when using very few reads to compute a consensus, SINGLe detects the true mutations (high sensibility) without increasing the false mutations as much as other methods do (specificity). We also tested SINGLe in a library of approximately 1,200 variants of KlenTaq, which are uniquely barcoded for clustering of the DNA strands. When many reads are available, Medaka and SINGLe report a similar number of mutations. But for variants with few reads, only 23% of the times both methods match, and Medaka predicts more mutations in the remaining 77% of the cases. Furthermore, when we plotted the location of the mutations detected by each method, we observed that the ones returned by SINGLe are spread along the strand, while other methods present some hot spot positions. We interpret them as a consequence of the systematic errors produced by nanopore sequencing and that SINGLe overcomes.

SINGLe needs an expected average number of mutations in the test set. Here, our reference sequence was assumed to be perfect, and we could evaluate precisely the average mutation rate in the test set because it originated from a controlled experimental mutagenesis protocol. In other situations, it would be possible to use short-read sequencing (e.g., Illumina) to evaluate this number. If the full sequence is submitted to short-read high-quality sequencing, it would even be possible to obtain more precise priors (e.g., specific to each position and nucleotide). Our approach would then be used to phase these statistical mutations to single long reads. An underlying assumption of our method is that the distribution of Qscore observed at a particular position for the wild-type base reflects appropriately the distribution of Qscore that would be observed for a variant base at that position. This approximation is necessary since the error model is built from a single sequence and hence has a single ‘‘true” base per position. Fortunately, the difference in Qscore distributions for ‘‘true” versus ‘‘error” seems large enough for our method to perform well within that approximation.

SINGLe needs to characterize the sequencing errors done on an appropriate reference sequence. Because it applies a probabilistic approach on the erroneous nanopore reads, irrespective of what mechanism actually causes the systematic errors, it should not depend on the genes’ properties. On the other hand, SINGLe is limited to analyze variants that are close neighbors of the reference and where mutations can be considered independent. We did not try to adapt the method to detect alterations beyond point replacements or deletions, which may require a more complex analysis pipeline. Encouragingly, variant 6 contained 2 contiguous mutations and was properly analyzed by our consensus approach. Finally, to our knowledge, SINGLe is the first tool fully focused on analyzing gene libraries sequenced by standard nanopore technology. Our approach provides a large improvement of signal to noise at very little experimental effort or throughput reduction. The reads of the reference sequences, needed to train SINGLe, can be obtained simultaneously with the libraries using standard barcoding protocols and only use a small fraction of the sequencing throughput. There are no other modifications to the experimental protocol, and the computational error correction process can be simply added to any analysis pipeline after basecalling. SINGLe allows an exploitation of the sequencing throughput 10 to 20 times better than Medaka and 4 times better than Nanopolish, but with a better performance, as we found that Nanopolish systematically misses some mutations. Therefore, SINGLe can be combined with experimental methods to obtain more accurate consensus sequences of large libraries of long genetic elements.

SINGLe is available as an R package that fits the errors on reads of a reference sequence; it then assigns p_SINGLe_ values on a library and a consensus sequence can be computed if a table with the barcodes in each read is provided. Its inputs are the .sam files obtained after a minimap2 alignment and samtool nucleotide count, the prior mutational rate, and the reference sequence.

## Methods

### Samples preparation and sequencing

KlenTaq wild-type and 7 variants: the wild-type gene was amplified using a high-fidelity PCR (Q5 polymerase from NEB Ipswich, MA, USA) from a stored plasmid following NEB recommendations with primers GGGATTATTCTTTGGCGCTCAGCCAAT and ACCATGCGTCTGCTGCATGAAT. The mutants were obtained via epPCR using the GeneMorph II kit (Agilent Santa Clara, CA, USA). We started from 1.1 nM dam-methylated DNA, and we used the same primers as for the wild-type. Thermocycling was performed as follows: 95°C for 2 minutes, followed by 25 cycles of [95°C for 30 seconds + 65°C for 30 seconds + 72°C for 2 minutes] and a final extension at 72°C for 10 minutes. In both cases, we digested the PCR product with DpnI (NEB) and purified it using columns (Macherey-Nagel Düren, Germany). We put the genes in a pIVEX vector via Gibson assembly (NEB Hi-Fi DNA assembly) using 125 ng gene DNA (93 ng for wild-type gene) and 100 ng vector in an approximately 2:1 insert/vector molar ratio and incubated for 15 minutes at 50°C. We purified and concentrated DNA with a Zymo Research (CA, USA) kit. We transformed the product into chemocompetent KRX bacteria. We spread them on a Petri dish with LB and ampicillin (one plate for the library, another for the wild-type). We incubated them overnight at 37°C, and the next day, we randomly picked some clones from the library and from the wild-type. We verified the presence of the plasmid via colony PCR using DreamTaq polymerase (Thermo Fisher Waltham, MA, USA). Positive clones were grown overnight in liquid LB with antibiotics and mini-prepped to obtain plasmid DNA. A fraction of the plasmid was used for high-quality sequencing (Sanger sequencing). Another fraction of each clone was used for amplification by PCR with Q5 polymerase (NEB) in independent tubes. We used primers that included the minION barcode adapters: ACTTGCCTGTCGCTCTATCTTCAGTGTGCTGGAATTCGCCCTTTTA and TTTCTGTTGGTGCTGATATTGCAGACCACAACGGTTTCCCTCTAGAAATA. Thermocycling was performed as follows: 98°C for 30 seconds, 23 cycles of [98°C for 10 seconds + 59°C for 30 seconds + 72°C for 1 minute], and final extension at 72°C for 2 minutes. We digested the product with Dpn1 (NEB) and gel purified it using the Macherey-Nagel kit. We proceeded to sequencing following standard Oxford Nanopore protocols. We used 1 barcode for wild-type and 1 for each of the mutants 1 to 7. We used kits EXP-PCB001 for barcoding, SQK-LSK108 for ligation, and EXP-LLB001 for flow cell loading. minION flow cell version was R9.4/FLO-MIN106, and thus the sequencing was 1D. After data preprocessing (see section on minION reads preprocessing), we had the following reads per variant: 716 (variant 1), 453 (variant 2), 645 (variant 3), 428 (variant 4), 422 (variant 5), 369 (variant 6), and 358 (variant 7).

KlenTaq large library: Mutants were obtained via epPCR using the GeneMorph II kit (Agilent). We started from 0.12 ng DNA (pIVEX vector containing KlenTaq gene). We used primers GCCAGTGTGCTGGAATTCGCCCTTTTATTAATG and CCCTCTAGAAATAATTTTGTTTAACTTTAAGAAGGAGATATACCATG. Thermocycling was performed as follows: 95°C for 2 minutes, followed by 30 cycles of [95°C for 30 seconds + 58°C for 30 seconds + 72°C for 2 minutes] and final extension at 72°C for 10 minutes. We digested the product with DpnI (NEB) for 1 hour and purified it using columns (Macherey-Nagel). We put the mutagenized genes in a pIVEX vector, previously amplified with primers that incorporated a random barcode downstream the STOP codon (aattccagcacactggcDHVBDHVBDHVBDHVBDHVBDHVBDHVBDHVBDHVBaagcccgaaaggaagctgag and ttaaagttaaacaaaattatttctagagggaaaccgttg). Previously, a SalI site was added into the pIVEX vector, upstream the T7 promoter. We used NEB Hi-Fi DNA assembly using 100 ng vector DNA and 103 ng insert DNA, in a 2:1 insert/vector molar ratio, and incubated for 15 minutes at 50°C. We purified and concentrated DNA with a Zymo Research kit. We transformed the product into chemocompetent T7 Express lysY/Iq bacteria (NEB C3013I) via heat shock and spread them on a Petri dish with LB and ampicillin. We incubated overnight at 37°C and obtained around 1,000 colony-forming units (CFU). We grew the library overnight at 37°C in liquid LB with ampicillin and extracted the plasmid using the Macheray-Nagel kit. We digested the library plasmid with EcoRI-HF and SalI-HF from NEB in rCutSmart buffer at 37°C for 1 hour 40 minutes, plus an inactivation step at 65°C for 20 minutes. We added proteinase K and incubated at 37°C for 15 minutes. We gel purified the sample to keep the fragment containing the KlenTaq gene and used the Zymo Research kit for concentration and purification of the DNA. We dialyzed our sample using Millipore membranes for 1 hour. For Oxford Nanopore sequencing, we used a flongle (version FLO-FLG001) and SQK-LSK110 kit for sample preparation. We started with 110 fmol and loaded 50 fmol into the flow cell.

### minION reads preprocessing

minION raw data were basecalled using ONT Guppy version 5.1 using model dna_r9.4.1_450bps_sup.cfg. We kept reads that had a length of 1,700 to 2,100 nucleotides and a mean Qscore larger than 10 (except for the large library, for which we used a Qscore cutoff of 15). For the wild-type and the 7 variants, we also used Guppy for de-multiplexing. For the KlenTaq large library, we used a custom script made in the lab to group barcodes by exact match. Sequences were aligned to the reference wild-type using minimap2 version 2.21 [16], using the options minimap2 -ax map-ont –sam-hit-only. We used samtools 1.7 [17] to create sorted bam files via the commands samtools view -S -b and samtools sort.

### Consensus by Nanopolish

We used the scripts provided by Oxford Nanopore, multi_to_single_fast5 and single_to_multi_fast5 (version 4.0), to split fast5 files and reassemble them according to the associated barcode. We then used the commands nanopolish index, samtools sort, samtools index, and nanopolish variants –consensus, according to the Nanopolish manual, to compute the consensus. Nanopolish version is 0.13.3.

### Consensus by Medaka

We used racon (version 1.4) to polish our sequences and used it as an input in the consensus computation by Medaka (version 1.4), which we ran by the command line medaka_consensus with default parameters.

### Consensus by NextPolish

We used NextPolish (version 1.4.1) with options task = best, rerun = 3, genome = KlenTaq wild-type sequence, and lgs_options -min_read_len 1k -max_depth 100.

## Additional Files

**SINGLe_Supplementary_reviewed.pdf** contains the following information:

**Sequence S1:** Nucleotide sequence of the wild type gene of KlenTaq

**Table S1:** Mutations on the seven KlenTaq variants tested (small set).

**Table S2:** Consensus sequence for the ten most frequent variants in the KlenTaq library using all available reads.

**Figure S1:** Normalized distribution of nucleotide’s Qscore returned by Guppy basecaller during the sequencing of the wild type gene of KlenTaq.

**Figure S2:** Errors per position produced by Guppy basecaller on Nanopore reads of the wild type gene of KlenTaq.

**Figure S3:** Analysis of the correlations between Qscores of consecutive nucleotides in a DNA strand read by a Nanopore Sequencer.

**Figure S4:** Success rate of consensus computation when sorting or not the nucleotides within homopolymers.

**Figure S5:** Signal to noise ratio, without weighting the counts by p_right_.

**Figure S6:** Analysis of the quality of the consensus sequence obtained with different methods over the reads of the small set of KlenTaq mutants.

**Figure S7:** Success rate of the consensus computation from subsets of available reads for the ten most frequent variants in the KlenTaq library.

**Figure S8:** Mismatches to wild type on the consensus computed by all tested methods by position, and classified according to the number of reads available for the consensus computation.

**Figure S9:** Comparison of mutational bias reported by the manufacturer of the epPCR kit, and the obtained mutations on the reads of the large KlenTaq library, according to the method used to compute consensus.

**Figure S10:** Analysis on how many Nanopore reads are needed to fit SINGLe.

**Figure S11:** Analysis on how do reverse and forward reads differ in Nanopore sequencing.

giac102_GIGA-D-22-00024_Original_Submission

giac102_GIGA-D-22-00024_Revision_1

giac102_GIGA-D-22-00024_Revision_2

giac102_Response_to_Reviewer_Comments_Original_Submission

giac102_Response_to_Reviewer_Comments_Revision_1

giac102_Reviewer_1_Report_Original_Submission Jeroen de Ridder -- 5/9/2022 Reviewed

giac102_Reviewer_1_Report_Revision_1 Jeroen de Ridder -- 8/15/2022 Reviewed

giac102_Reviewer_2_Report_Original_Submission Sebastian JÃ¼nemann -- 5/13/2022 Reviewed

giac102_Supplemental_File

## Availability

Project name: SINGLe

Project home page: https://github.com/rocioespci/single, also available in bioconductor 10.18129/B9.bioc.single

Operating system: Platform independent

Programming language: R (> = 4.1).

Other requirements: Imports R packages Biostrings, BiocGenerics, dplyr, GenomicAlignments, IRanges, methods, reshape2, rlang, Rsamtools, stats, stringr, tidyr, utils. To create input files, minimap2 and samtools are required (or equivalent software to align data to reference and create a bam file).

License: MIT

Software is registered at scicrunch.org (identifier SINGLe, RRID:SCR_022810) and biotools (https://bio.tools/SINGLe).

## Data Availability

The dataset supporting the results of this article is available in the European Nucleotide Archive repository, ERP135743, run ERR8778685 (nanopore reads of 7 mutants and wild-type), and ERR8778797 (nanopore reads of library) and in GigaDB [18] (Sanger sequencing for the 7 mutants of KlenTaq, sequence of wild-type KlenTaq gene, and the full code and processed data to reproduce the figures in the article and supplementary material).

## Funding

Supported by the European Research Council, H2020 Marie Sklodowska-Curie Research and Innovation program, 845976 (R. Espada) and European Research Council, Consolidator Grant No. 647275 ProFF (Y. Rondelez).

## Competing Interests

The authors declare that they have no competing interests.

## Abbreviations

epPCR: error-prone PCR; SINGLe: SNPs In Nanopore reads of Gene Libraries; Qscore: quality score; Sk: skewness; ROC: receiver operating characteristic; HMM: hidden markov models; SNP: single-nucleotide polymorphism.

## Authors' Contributions

R.E. and Y.R. designed research and conceptualization. Y.R. performed supervision of the research. R.E. and A.D.M. performed experimental research. R.E., N.Z., and Y.R. performed methodology and formal analysis. R.E. wrote the software. R.E. and Y.R. wrote the manuscript, and A.D.M. and N.Z. reviewed and edited the manuscript.

## References

[1] Oxford Nanopore Technologies. https://nanoporetech.com/. Accessed 1 February 2022.

[2] Sze MA, Schloss PD. The impact of DNA polymerase and number of rounds of amplification in PCR on 16S rRNA gene sequence data. mSphere. 2019;4(3). 10.1128/mSphere.00163-19 PMC6531881 31118299

[3] Thibodeau ML, O'Neill K, Dixon K, et al. Improved structural variant interpretation for hereditary cancer susceptibility using long-read sequencing. Genet Med. 2020;22(11):1892–7. 32624572 10.1038/s41436-020-0880-8 PMC7605438

[4] Wang Y, Zhao Y, Bollas A, et al. Nanopore sequencing technology, bioinformatics and applications. Nat Biotechnol. 2021;39(11):1348–65. 34750572 10.1038/s41587-021-01108-x PMC8988251

[5] Sedlazeck FJ, Rescheneder P, Smolka M, et al. Accurate detection of complex structural variations using single-molecule sequencing. Nat Methods. 2018;15(6):461–8. 29713083 10.1038/s41592-018-0001-7 PMC5990442

[6] Gong L, Wong C-H, Cheng W-C, et al. Picky comprehensively detects high-resolution structural variants in nanopore long reads. Nat Methods. 2018;15(6):455–60. 29713081 10.1038/s41592-018-0002-6 PMC5990454

[7] Loman NJ, Quick J, Simpson JT. A complete bacterial genome assembled de novo using only nanopore sequencing data. Nat Methods. 2015;12(8):733–5. 26076426 10.1038/nmeth.3444

[8] Vaser R, Sović I, Nagarajan N, et al. Fast and accurate de novo genome assembly from long uncorrected reads. Genome Res. 2017;27(5):737–46. 28100585 10.1101/gr.214270.116 PMC5411768

[9] Medaka https://github.com/nanoporetech/medaka. Accessed 23rd October 2022.

[10] Li C, Chng KR, Boey EJH, et al. Incseq: accurate single molecule reads using nanopore sequencing. Gigascience. 2016;5(1):s13742–016. 10.1186/s13742-016-0140-7 PMC4970289 27485345

[11] Karst SM, Ziels RM, Kirkegaard RH, et al. High-accuracy long-read amplicon sequences using unique molecular identifiers with Nanopore or PacBio sequencing. Nat Methods. 2021;18(2):165–9. 33432244 10.1038/s41592-020-01041-y

[12] Krishnakumar R, Sinha A, Bird SW, et al. Systematic and stochastic influences on the performance of the MinION nanopore sequencer across a range of nucleotide bias. Sci Rep. 2018;8(1):3159. 29453452 10.1038/s41598-018-21484-w PMC5816649

[13] Huang Y-T, Liu P-Y, Shih P-W. Homopolish: a method for the removal of systematic errors in nanopore sequencing by homologous polishing. Genome Biol. 2021;22(1):95. 33789731 10.1186/s13059-021-02282-6 PMC8011154

[14] GeneMorph II Random Mutagenesis Kit. Instruction manual. https://www.agilent.com/cs/library/usermanuals/Public/200550.pdf. Accessed 23rd October 2022.

[15] Hu J, Fan J, Sun Z, et al. NextPolish: a fast and efficient genome polishing tool for long-read assembly. Bioinformatics. 2020;36(7):2253–5. 31778144 10.1093/bioinformatics/btz891

[16] Li H. Minimap2: pairwise alignment for nucleotide sequences. Bioinformatics. 2018;34(18):3094–100. 29750242 10.1093/bioinformatics/bty191 PMC6137996

[17] Li H, Handsaker B, Wysoker A, et al. The sequence alignment/map format and SAMtools. Bioinformatics. 2009;25(16):2078–9. 19505943 10.1093/bioinformatics/btp352 PMC2723002

[18] Espada R, Dramé-Maigné A, Zarevski N, et al. Supporting data for “Accurate gene consensus at low nanopore coverage.”. GigaScience Database. 2022. 10.5524/102265. PMC9646519 36352541

