# Supplementary material for: Accurate gene consensus at low nanopore coverage
Source: Gigascience. 2022 Nov 9;11:giac102. doi: 10.1093/gigascience/giac102 (PMC9646519; doi:10.1093/gigascience/giac102)
Supplement: giac102_GIGA-D-22-00024_Original_Submission [file giac102_giga-d-22-00024_original_submission.pdf]

GigaScience

Accurate gene consensus at low nanopore coverage

--Manuscript Draft--

**Manuscript Number:**	GIGA-D-22-00024
**Full Title:**	Accurate gene consensus at low nanopore coverage
**Article Type:**	Technical Note
**Funding Information:**	H2020 European Research Council (845976) — PhD Rocio Espada<br>H2020 European Research Council (647275) — PhD Rondelez Yannick
**Abstract:**	Nanopore technologies allow high throughput sequencing of long strands of DNA at the cost of a relatively large error rate. This limits its use in the reading of amplicon libraries in which there are only a few mutations per variant and therefore they are easily confused with the sequencing noise. Consensus calling strategies reduce the error but sacrifice part of the throughput on reading typically 30 to 100 times each member of the library.<br>In this work, we develop SINGLe (SNPs In Nanopore reads of Gene Libraries), an error correction method to reduce the noise in nanopore reads of amplicons containing point variations. SINGLe exploits that in an amplicon library, all reads are very similar to a wild type sequence, from which it is possible to experimentally characterize the position-specific systematic sequencing error pattern. Then, it uses this information to reweight the confidence given to nucleotides that do not match the wild type in individual variant reads, and incorporates it on the consensus calculation.<br>We tested SINGLe in a mutagenic library of the KlenTaq polymerase gene, where the true mutation rate was below the sequencing noise. We observed that contrary to other methods, SINGLe compensates for the systematic errors made by the basecallers. Consequently, SINGLe converges to the true sequence 5 times faster than other methods.
**Corresponding Author:**	Rondelez Yannick, PhD<br>ESPCI Paris/CNRS/PSL<br>Paris, FRANCE
**Corresponding Author Secondary Information:**	
**Corresponding Author's Institution:**	ESPCI Paris/CNRS/PSL
**Corresponding Author's Secondary Institution:**	
**First Author:**	Rocio Espada
**First Author Secondary Information:**	
**Order of Authors:**	Rocio Espada<br>Adèle Dramé-Maigné<br>Nikola Zarevski<br>Rondelez Yannick, PhD
**Order of Authors Secondary Information:**	
**Additional Information:**	
**Question**	**Response**
Are you submitting this manuscript to a special series or article collection?	No
**Experimental design and statistics**	Yes

Powered by Editorial Manager® and ProduXion Manager® from Aries Systems Corporation

Full details of the experimental design and statistical methods used should be given in the Methods section, as detailed in our Minimum Standards Reporting Checklist. Information essential to interpreting the data presented should be made available in the figure legends.<br><br>Have you included all the information requested in your manuscript?	
**Resources**<br><br>A description of all resources used, including antibodies, cell lines, animals and software tools, with enough information to allow them to be uniquely identified, should be included in the Methods section. Authors are strongly encouraged to cite Research Resource Identifiers (RRIDs) for antibodies, model organisms and tools, where possible.<br><br>Have you included the information requested as detailed in our Minimum Standards Reporting Checklist?	Yes
**Availability of data and materials**<br><br>All datasets and code on which the conclusions of the paper rely must be either included in your submission or deposited in publicly available repositories (where available and ethically appropriate), referencing such data using a unique identifier in the references and in the “Availability of Data and Materials” section of your manuscript.<br><br>Have you have met the above requirement as detailed in our Minimum Standards Reporting Checklist?	Yes

Powered by Editorial Manager® and ProduXion Manager® from Aries Systems Corporation

Manuscript
Click here to access/download;Manuscript;SINGLE_technicalnote.pdf

Title page

Title:

Accurate gene consensus at low nanopore coverage

Authors:

Espada Rocío.

Gulliver Lab, ESPCI Paris, PSL University, CNRS, 75005 Paris, France.

Contact: rocio.espada@espci.fr.

Zarevski Nikola. Gulliver,

Gulliver Lab, ESPCI Paris, PSL University, CNRS, 75005 Paris, France.

Contact: niko.zarevski@gmail.com

Dramé-Maigné Adèle.

Gulliver Lab, ESPCI Paris, PSL University, CNRS, 75005 Paris, France.

Contact: docdeldou@gmail.com

Rondelez Yannick.

Gulliver Lab, ESPCI Paris, PSL University, CNRS, 75005 Paris, France.

Contact: yannick.rondelez@espci.fr.

Corresponding author

Abstract

Background Nanopore technologies allow high throughput sequencing of long strands of DNA at the cost of a relatively large error rate. This limits its use in the reading of amplicon libraries in which there are only a few mutations per variant and therefore they are easily confused with the sequencing noise. Consensus calling strategies reduce the error but sacrifice part of the throughput on reading typically 30 to 100 times each member of the library.

Findings In this work, we develop SINGLe (SNPs In Nanopore reads of Gene Libraries), an error correction method to reduce the noise in nanopore reads of amplicons containing point variations. SINGLe exploits that in an amplicon library, all reads are very similar to a wild type sequence, from which it is possible to experimentally characterize the position-specific systematic sequencing error pattern. Then, it uses this information to reweight the confidence given to nucleotides that do not match the wild type in individual variant reads, and incorporates it on the consensus calculation.

Conclusions We tested SINGLe in a mutagenic library of the KlenTaq polymerase gene, where the true mutation rate was below the sequencing noise. We observed that contrary to other methods, SINGLe compensates for the systematic errors made by the basecallers. Consequently, SINGLe converges to the true sequence 5 times faster than other methods.

Keywords:

Nanopore sequencing; consensus sequence; low coverage; gene library

Findings

Background

Nanopore is a powerful technology for high throughput DNA sequencing, currently commercialized by Oxford Nanopore Technologies [1]. It provides sequence base calls reconstructed from conductivity records during the translocation of a single DNA molecule through a protein pore. This approach offers portability and real time sequencing, using simple experimental protocols, and for a relatively low cost. A minION device can read DNA strands of various lengths, from PCR products up to megabase genomic fragments, and current versions return at least 5×10^9 bases in one run. Therefore, it is an attractive device for sequencing libraries of amplicons that are too long for other next generation sequencing technologies. Unfortunately, nanopore's relatively high error rate (≈ 6-15%) prevents the accurate detection of point genetic variation directly from individual reads [2][3][4]. Previous work aiming at high quality sequencing from nanopore data has concentrated on polishing tools such as Nanopolish [5], Racon [6] and Medaka [7]. These approaches start from a draft assembly and use the coverage depth to compute an averaged consensus at each position, via various computational approaches. Nanopolish, which uses the raw nanopore signal, reports an accuracy over 99.5% for a 29x sequencing coverage, while Racon reports 97% for 59x. Medaka reports 98% of accuracy in detection of single nucleotide polymorphisms (SNP) with a coverage of 100x. While these tools primarily apply to genome assembly, a number of experimental protocols were developed in order to leverage these pipelines in the specific case of amplicon library sequencing. These strategies aim to read and associate several replicates of the same molecule. This has been achieved by creating sequence concatenates using rolling circular amplification [8], which retrieved an accuracy of 99.5% for coverage of 150x, and via gene barcoding prior to amplification [9] with a reported accuracy over 99.9% for 25x coverage. Inconveniently, these methods add experimental efforts to the preparation of the sample prior to sequencing, are sensitive to bias occurring during the PCR amplification steps [10], and reduce the

3

number of different variants that can be studied, because a part of the sequencing throughput is invested in reading each sequence many times.

Here, we propose a computational tool for amplicon sequencing, which applies in the case where the library is highly diverse but has low variability, i.e. it contains many different sequences differing from each other by only a few point mutations. This is for example the case in directed evolution experiments, where the genetic libraries typically originate from a single ancestral sequence (the wild type) that has been submitted to limited randomization, for example using error-prone replication [10]. There is an increasing interest in using NGS technologies to follow directed evolution experiments [11][12], but specific tools are not yet available. We developed a computational protocol, SINGLe (SNPs In Nanopore reads of Gene Libraries), which improves variant detection in individual reads from such libraries, for which a reference gene is known. In contrast to previous work, this approach uses standard 1D protocol minION sequencing and library preparation, and has a very limited impact on throughput.

We base our method on two observations, made during the sequencing of many identical copies of the reference sequence. First, the confidence or quality scores (Qscore) assigned by the basecalling process to each nucleotide are usually low when a wrong nucleotide is assigned (Figure 1A), as expected. Second, the errors are not homogeneously distributed, and they are more frequent in some positions of the DNA sequence (Figure 1B and Figure S1). Previous work has also shown that nanopore sequencing produces some systematic errors [2,13][14], even for high accuracy basecalling. These observations suggest that it should be possible to reduce the non-random part of the sequencing errors, using the information contained in the Qscore. SINGLe consists of two steps: the first one uses the reference reads to build a statistical model of the error pattern. Here, we used a direction, position and nucleotide-specific logistic regression. In the second step, this information is used to re-analyze basecalls for the variant library and to update the confidence value of each nucleotide read in this dataset.

4

We tested SINGLe using the gene of KlenTaq, a truncated variant of the well-known Taq polymerase, of approximately 1.7 kb in length. We trained our model on approximately 6000 reads of the wild type. We first tested it in a small set of seven known mutants containing 2 to 9 point mutations, and later on a full library of ~1200 variants. Our computational correction reduced the sequencing noise, allowing a better identification of true point mutations. This reduced the number of reads needed to obtain a trustable consensus sequence to as low as 5-7 reads, outperforming the state-of-the-art tools for consensus computation of nanopore sequencing, Medaka and Nanopolish.

Method

SINGLe is designed to identify true mutations in gene libraries as those produced by error-prone PCR (ePCR). These libraries have a few number of mutations per gene (typically less than 20 mutations per kb). In this range, we assume that when the sequencer reports a wild type nucleotide is correct, and that we can neglect the times a mutation is reported as a wild type. Then, the challenge is to identify when the sequencer is correct or not if it reports a mismatch.

We used a nanopore sequencer to read 5847 strands of the wild type KlenTaq gene (length 1662 nucleotides), for which we have a ground truth sequence (in supplementary Table S1). In this data set, we can confidently attribute mismatches between the read sequences and the known wild type as sequencing errors, and matches as correct reads. In Figure 1A, we plot the distribution of correct/error nucleotides according to the Qscore returned by Oxford nanopore basecaller, Guppy. The distribution for the errors is centered around the lower Qscores. Unfortunately, the distribution for the correct reads is not completely separated from the errors, thus a classification based only on the Qscore is not possible. In figures 1B and S1, we plot the counts of errors by position and nucleotide. This distribution is not homogeneous, indicating that nanopore sequencing produces some systematic errors.

We used the reads of the wild type gene to count the errors produced by the sequencer in each position and nucleotide and plot it against the Qscore reported by guppy (Figure

5

1C). We fitted this relation by a logistic regression, which provided a classifier able to convert the reported Qscore to the probability that this read is indeed correct (p_{right}). Nevertheless, as it is computed over wild type reads which do not contain true mutations, the model is heavily biased against mutation. This is not representative of the actual proportion of errors/correct reads present in the mismatches of a set of mutants. To adapt the classifier, an a priori expectation of mutations ($p_{prior-right}$) is needed, which must come from independent information. In the present case, the variant sequences originate from an error prone PCR (ePCR) process, for which we possess an estimate of the mutation frequency. We also obtain the *a priori* expectation of an observed mismatch to be a sequencing error ($p_{prior-error}$) as the sequencing error rate at that position in the wild type set. We therefore compute $p_{prior-right}$ and $p_{prior-error}$, which we use to reweight the wild type set before fitting. This shifts the logistic regression towards the higher Qscore, allowing the classifier to accept a number of observed mismatches consistent with the prior expectation (Figure 1D).

The fits are done independently for each position and possible mismatched nucleotides. To include deletions in this analysis (which do not have a Qscore assigned by the basecaller), we fixed their confidence value as the minimum of the Qscore of their nearest neighbors in the nucleotide sequence. This decision was inspired by the observation that Qscore is correlated between consecutive nucleotides (Figure S2). Insertions were ignored. We also separated the fits for forward and reverse reads. All together, we obtained 13296=1662×4×2 regressions, one for each position of the gene (1662 bp), for each non-wild type nucleotide or deletion possible (4 possibilities in total for each position) and for the forward and reverse sense of sequencing.

Finally, the regressions are used to re-scored the mismatches observed in the mutant library. We compute the consensus of a set of reads by weighting the nucleotides' frequencies by SINGLe's p_{right}. The nucleotide (or deletion) with the highest weighted count is kept as the consensus in that position. Homopolymers regions were sorted so that gaps are always at the 3' side on the forward strand. To compare, we also computed the variant consensus sequence (VCS) using Qscores from guppy basecaller or by

unweighted majority vote. In these cases, we did not sort the homopolymers region as it had a detrimental effect (see Figure S5).

7

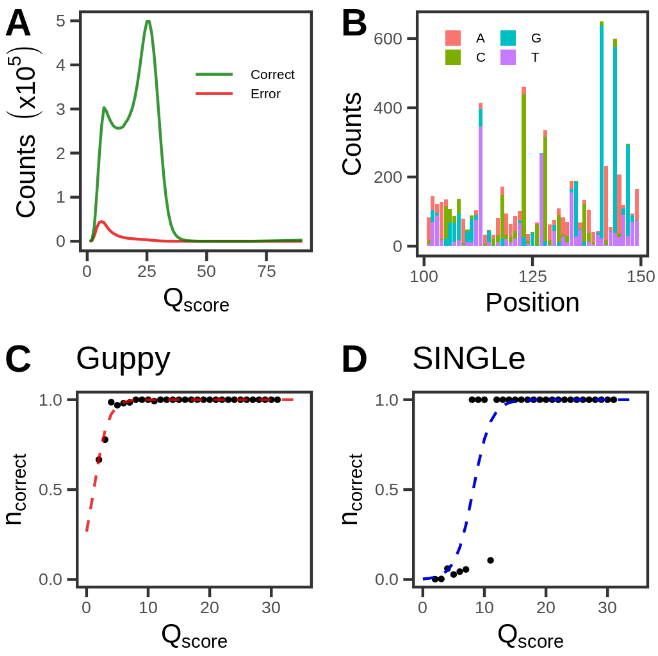

Figure 1: A Distribution of bases' Qscore returned by Guppy basecaller on the sequencing of wild type klen taq gene, classified as correct reads (green) and errors (red). Guppy assigns low Qscores to sequencing errors. **B** Sequencing errors per position produced by guppy basecaller on the wild type klen taq gene. Colors indicate the nucleotide informed by the basecaller, and wild type (correct) nucleotides are not shown. Errors are not homogeneously distributed. **C** Example of logistic regression over reads of a known wild type sequence. Black dots are the proportion of correct reads against the Qscore reported by guppy basecaller. Dashed red line is the logistic regression performed. **D** Same plots as C after data was weighted according to the prior probability of actual mutation.

Analyses

We tested SINGLe in two sets: first seven variants of KlenTaq (named #1 to #7) which we obtained from seven independent bacterial clones and barcoded using the nanopore barcoding kit before sequencing. Their true sequence was obtained by Sanger. They contain 2-9 point mutations (supplementary table S2). Variants #1 to #5 only present nucleotide substitutions. Variant #6 has 7 substitutions, two of them in consecutive

positions, and one deletion in a non homopolymer region. Variant #7 has 5 substitution and a deletion in a homopolymer ('GG' to 'G-'). The second set is a larger mutagenesis library of KlenTaq obtained by ePCR, with a mean mutation rate of 8muts/kB and it contains ~1200 variants. Each of them is associated with a barcode located downstream the STOP codon. The barcode is 36 nucleotides long and has the structure $(HVBD)_n$ to avoid homopolymers longer than 3 bases.

Signal to noise ratio

The high error rate in nanopore sequencing makes it difficult to distinguish actual mutations from sequencing errors in single reads. A straightforward procedure to filter errors is to only trust read positions which have a high probability of being correct. In this section, we compare how this threshold process performs when using, either, the raw Qscores returned by guppy basecaller (converted to a probability as $p_{guppy}=1-10^{-Q/10}$) or p_{right} after fitting with a logistic regression using prior probabilities (SINGLe).

We used the reads on the seven variants of KlenTaq, for which we know if each mismatch is a sequencing error or an actual mutation. We defined signal as the number of mismatches known to be mutations with a p_{right} higher than the threshold (true positives), and noise as the number of those mismatches known to be non-mutated positions (false positives). The counts are weighted by p_{right} for each nucleotide. Results are shown in Figure 2A, both as a ROC curve and as a signal-to-noise ratio. For all thresholds, SINGLe has a higher signal to noise ratio (up to 6 times higher, depending on the cut off), thus facilitating the identification of actual mutations. This remains true when no cut off is applied (cut off = 0). Results are similar if we count the number of mismatches over the threshold without weighting them by p_{right} (supplementary figure S3).

Consensus sequences

One strategy for lowering noise in nanopore sequencing consists in computing a variant consensus sequence (VCS), i.e. reading several times a variant and using these reads to calculate the most likely sequence. Usually, average on more reads leads to a more

accurate VCS. We hypothesized that the corrected weights returned by SINGLe will help to converge faster on the actual sequence, as they increase the confidence on mutations over the sequencing errors. We tested this hypothesis over the set of 7 known KlenTaq variants.

For the VCS, we proceeded as explained in the Methods section. To compare with existing methods, we also computed the consensus by Medaka (after polishing with Racon) and Nanopolish. We computed the consensus on a set of 3 to 50 sequences drawn randomly from all available reads, and repeated 50 times for each set size. In Figure 2B, we plot the success rate on VCS computation, i.e. how many times the obtained consensus matches exactly the true sequence. For variants #1 to #6, the convergence is faster when using SINGLe weights: perfect consensuses are obtained for more than 90% of attempts starting from 5-7 sequences. The closest performance is Nanopolish, which reaches the same success rate in 8 to 15 reads but fails for variant #1. Medaka requires more than 20 reads to reach 90% of success, and it does not converge for variant #4. Finally, using p_{guppy} or no weights has a poorer performance, not reaching 90% of success for 50 reads for any of the 6 variants. Notice that variant #6 has two consecutive mutations and they are properly detected. Variant #7 has a deletion in a homopolymer which is a challenging mutation to detect (Figure 2C). In this case, SINGLe needs 35 reads to converge to the true sequence, outperforming Medaka or the VCS computed with guppy Qscores or not scores (they need 45 reads). Only Nanopolish converges faster, with 15 reads.

When looking closer at the convergence to the true sequence in Figure 2D and S4, we see that erroneous consensus sequences predicted by, SINGLe typically only contain a few extra mismatches, even when averaging as few as 3 reads (typically one extra mismatch). On the other hand, Medaka starts from many more errors, returning over 15 wrong mismatches for 3 reads. Using Qscores or no weights for the VCS computation have similar performance as Medaka. Curiously, Nanopolish is the only method which misses mutations instead of reporting errors as mutations when few reads are used.

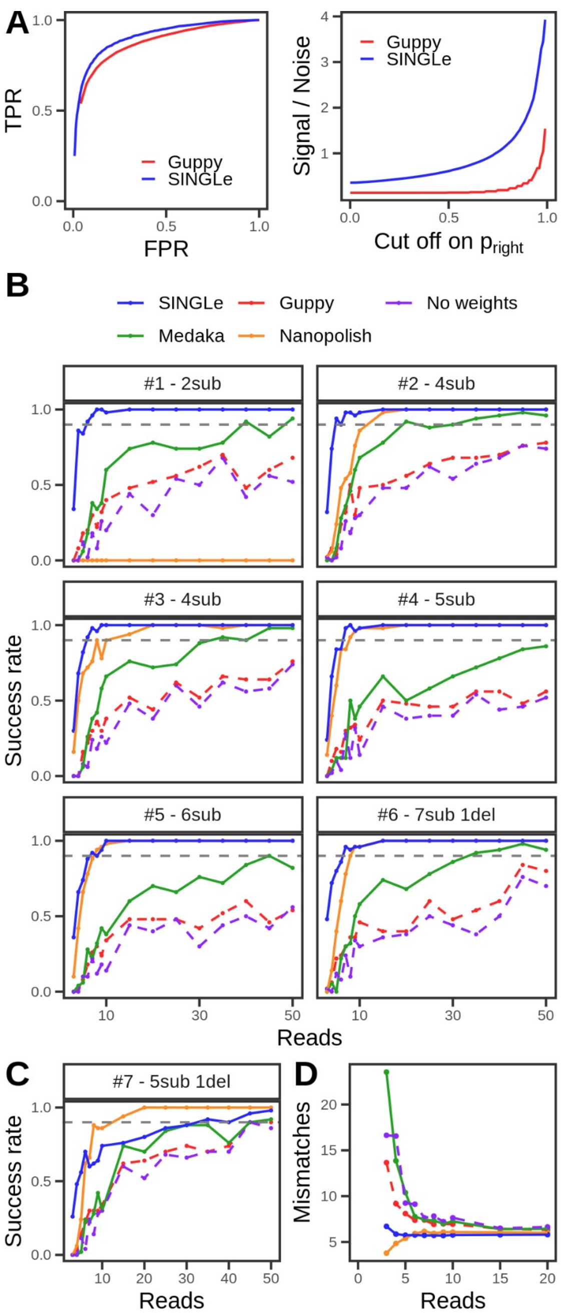

Figure 2: SINGLe improvement tested over the reads of seven known mutants of KlenTaq. **A** On the left, true positive rate (TPR) vs false positive rate (FPR) (or ROC curve). On the right, signal to noise ratio when classifying mismatches as mutations when they reach a p_{right} indicated on the x axis. **B and C** For each known sequence (#1 to #7), we took a subset of the available reads (x axis) and computed the consensus with various methods. We repeated 50 times for each set size. Y axis indicates the success rate on obtaining the correct sequence. On top of each plot we indicate the number of substitutions (sub) and deletions (del) with respect to the wildtype gene. SINGLe

outperforms all tested methods, with a success rate over 0.9 with as few as 5 to 7 reads (panel B). Variant 7 (panel C) contains a deletion in a homopolymer which requires more reads to be properly detected (35 for SINGLe, 15 for nanopolish, over 40 for other methods). **D** Mean number of mismatches among the 50 subsets for variant #3 (4 sub). SINGLe convergence fast and steady to the true number of mutations. Except nanopolish, all methods predict a higher number of mismatches than the actual mutations when few reads are used on the consensus calculation, and then decrease towards the correct number of mutations as more reads are included.

Consensus in a large gene library

We also tested SINGLe on a large library of mutants of the Klen Taq gene. The variants are unknown, but associated with a barcode downstream the STOP codon. We sequenced the library using nanopore, grouped the reads according to the barcode and computed the VCS for each of them. We first confirmed the consistency of the different consensus methods on this library run, with respect to the results obtained on known variants. We chose the most represented barcode (901 reads) and computed the VCS success rate using Medaka and SINGLe for subsets of reads. As ground truth we used the consensus computed with all available reads, which is the same for both methods. As shown in Figure 3A, SINGLe converges using 5 reads, while Medaka needs 15. Similar results were obtained for the other 9 most frequent barcodes using various methods for VCS (Table S3 and Figure S6).

We computed VCS using SINGLe or Medaka for all the mutants in our library, provided that the identifying barcode is present at least four times in our dataset, and compared the number of mutations reported by both methods (figure 3B). When there are at least 10 reads, both methods report the same consensus for 89% of the variants (blue bar on 0 differences). This value lowers to 56% for variants with 6 to 10 reads (green bar on - differences), and to only 23% for variants with 4 or 5 reads (red bar). When few reads are available, Medaka predicts more mutations than SINGLe (negative values on x axis of Figure 3B). This is consistent with the observation in Figure 2D: Medaka tends to predict

12

more mutations than there actually are, rather than missing true mutations. Less than 1% of all computed consensuses have more mutations by SINGLe than by Medaka. This hints that SINGLe is detecting the true sequences.

We claim that SINGLe reduces the error by correcting for the systematic errors in the basecalling. If so, the mutations predicted by SINGLe for a randomly mutated gene library should be homogeneously distributed along the sequence. In figure 3C we show that this is the case. When there are more than 10 reads available in each cluster, SINGLe and Medaka predict mutations that are similarly distributed throughout the gene, as reflected by the skewness of the distribution of mutation counts (sk), around 0.8 in both cases. When there are only 6 to 10 reads available, Medaka tends to predict mutations on some preferred spots, increasing sk to 3.8. The effect is even larger for 5 or less reads: Medaka shows strong systematic errors (sk around 7), while SINGLe’s remains at 1.3.

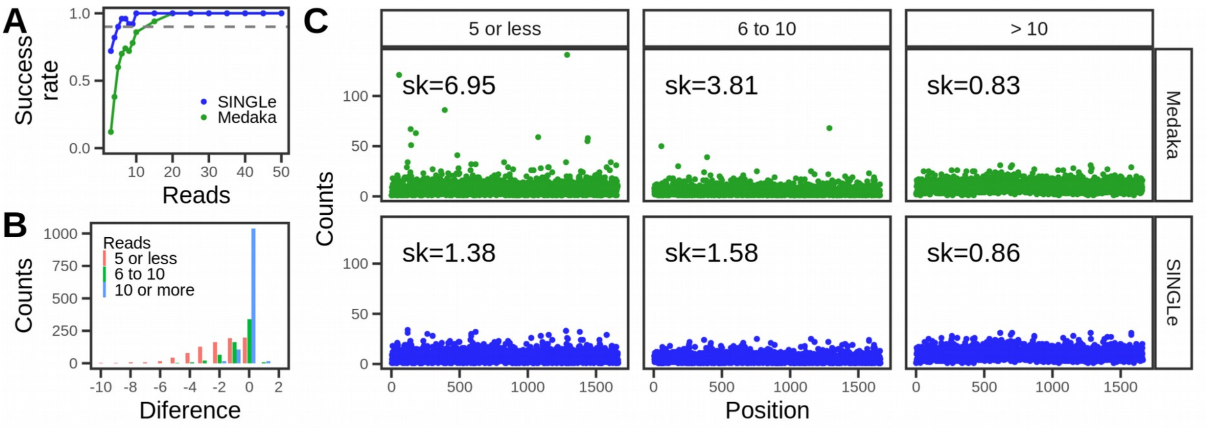

Figure 3: SINGLe improvement tested on a gene library. **A** For the most frequent variant in the library, success rate of the consensus computation using subsets of available reads. Convergence is similar to the seven variants tested before, with SINGLe converging with 5 reads and medaka with 15 reads. **B** Histogram of the difference of mismatches detected for each variant in the library between SINGLe and Medaka. A negative value on the x axis indicates that Medaka found more mismatches than SINGLe. **C** Mismatches to wild type on the consensus computed by Medaka (upper panels) and

13

SINGLe (lower panels) by position, and classified according to the number of reads available for the consensus computation. Medaka mismatches are systematic, with some positions overrepresented, especially when there are few available reads. To quantify the systematicity of the mismatches, we used the skewness (sk) of the distribution of counts.

Discussion

The relatively high error rate in single molecule nanopore sequencing limits some applications such as the analysis of libraries containing many different, but genetically similar, sequences. The approach we propose here, SINGLe, leverages the fact that the sequencing errors in this case are partly systematic, as previously noted [2,13]. We accumulate many reads from the reference gene to build a sequence-specific error model that can locally correct for the sequencing biases. Applying this procedure on a set of seven variants of the KlenTaq gene with an average mutation rate of 3 bases/kb, we showed that correcting the confidence values provides a large increase in the signal to noise ratio. Consequently, the consensus calling returns 90% of perfect results from typically 5-7 reads. This implies a faster convergence than other methods currently used: nanopolish requires 8 to 15 reads while Medaka needs at least 20 reads to achieve a similar performance. Therefore, a lower burden on the sequencing throughput is taken to obtain true consensus sequences. We observed that most methods tend to be confused by the sequencing errors and report more mutations than expected, rather than missing true substitutions. We also applied this procedure to compute consensus on a library of approximately ~1200 variants of KlenTaq, which are uniquely barcoded for clustering of the DNA strands. We observed that SINGLe predicts less mutations than Medaka, especially when there are available only a few reads of the same variant. Furthermore, when we plotted the location of the mutations detected by each method, we observed that the ones returned by SINGLe are spread along the strand, while Medaka presents

some hot spot positions. We interpret them as a consequence of the systematic errors produced by nanopore sequencing and that SINGLe helps to overcome.

An important ingredient of SINGLe is a correct prior for the number of mutations in the train and test set. Here, our reference sequence was assumed to be perfect, and we could evaluate precisely the average mutation rate in the test set, because it originated from a controlled experimental mutagenesis protocol. In other situations it would be possible to use short read sequencing, for example Illumina, to evaluate this number. If the full sequence is submitted to short read high quality sequencing, it would even be possible to obtain more precise priors, for example specific to each position and nucleotide. Our approach would then be used to phase these statistical mutations to single long reads. An underlying assumption of our method is that the distribution of Qscore observed at a particular position for the wild type base reflects appropriately the distribution of Qscore that would be observed for a variant base at that position. This approximation is necessary since the error model is built from a single sequence and hence has a single ‘‘true” base per position. Fortunately, the difference in Qscore distributions for ‘‘true” versus ‘‘error” seems large enough for our method to perform well within that approximation.

SINGLe needs to characterize the sequencing errors done on an appropriate reference sequence. As such it is limited to analyze variants which are close neighbors of the reference, and where mutations can be considered to be independent. We did not try to adapt the method to detect alterations beyond point replacements or deletions, which may require a more complex analysis pipeline. Encouragingly, variant 6 contained two contiguous mutations, and was properly analyzed by our consensus approach. Finally, to our knowledge SINGLe is the first tool fully focused in analyzing gene libraries sequenced by standard nanopore technology. Our approach provides a large improvement of signal to noise at very little experimental effort or throughput reduction. This is because only the reference DNA needs to be sequenced many times, compared to other methods where each library member requires multiple reads. In SINGLe, the reference DNA dataset can be obtained simultaneously with the libraries, using standard barcoding

 15

protocols. There are no other modifications to the experimental protocol, and the computational error correction process can be simply added to any analysis pipeline after basecalling. Therefore, in contrast to methods that increase accuracy at a high cost on throughput, SINGLe paves the way to a more accurate characterization of large libraries of long genetic elements.

The method is available as an R package that will fit the reads of a reference sequence and fit the p_{right} values on a library. It’s inputs are the sam files obtained after a minimap2 alignment and samtool nucleotides count, the prior mutational rate and the reference sequence.

Methods

Samples preparation and sequencing

KlenTaq wild type gene was amplified using a high fidelity PCR (Q5 polymerase from NEB) from a stored plasmid. See DNA sequence in supplementary table S1.

KlenTaq seven variants: Mutants were obtained via error prone PCR (ePCR) using Agilent’s kit GeneMorph II. We started from 1.1 nM of dam-methylated DNA. We used primers GGGATTATTCTTTGGCGCTCAGCCAAT and AC-CATGCGTCTGCTGCATGAAT. Thermocycling was performed as follows: 95°C for 2 min, followed by 25 cycles of [95°C for 30 sec + 65°C for 30 sec + 72°C for 2 min] and a final extension at 72°C for 10 min. We digested the product with DpnI (NEB) and purified it using columns (Macherey-Nagel). We put the mutagenized genes in a pIVEX vector via Gibson assembly (NEB Hi-Fi DNA assembly) using 125ng of gene DNA, 100 ng of vector in a 2:1

16

insert:vector molar ratio and incubated for 15 min at 50°C. We purified and concentrated DNA with a Zymo Research kit. We transformed the product into chemocompetent KRX bacteria. We spread them on a Petri dish with LB and Ampicillin. We incubated overnight and picked some clones randomly. We verified the presence of the plasmid via colony PCR (using DreamTaq polymerase from Thermofisher). Positive clones were grown overnight in liquid LB with antibiotics and mini-prepped to obtain the plasmid DNA. A fraction of the plasmid was used for high quality sequencing (Sanger sequencing), and another fraction used for minION sequencing. We used the plasmid DNA of each clone and the wild type gene for amplification by PCR with Q5 polymerase (NEB) in independent tubes. We used primers which included the minION barcodes adapters: ACTTGCCTGTCGCTC-TATCTTCAGTGTGCTGGAATTCGCCCTTTTA and TTTCTGTTGGTGCTGATATTGCAGACCA-CAACGGTTTCCCTCTAGAAATA. Thermocycling was performed as follows: 98C for 30sec, 23 cycles of [98C for 10sec + 59C for 30sec + 72C for 1min], final extension at 72C for 2min.

We digested the product with Dpn1 (NEB), gel purified it using Macherey-Nagel kit. We proceeded following standard Oxford Nanopore protocols for minION. We used one barcode for wild type and one for each of the mutants 1 to 7.We used kits EXP-PCB001 for barcoding, SQK-LSK108 for ligation, EXP-LLB001 for flow cell loading. minION flow cell version was R9.4/FLO-MIN106, thus the sequencing was 1D.

KlenTaq large library: Mutants were obtained via error prone PCR (ePCR) using Agilent's kit GeneMorph II. We started from 0.12ng of DNA (pIVEX vector containing KlenTaq gene). We used primers GCCAGTGTGCTGGAATTCGCCCTTTTATTAATG and CCCTCTAGAAATAATTTTGTTTAACTTTAAGAAGGAGATATACCATG. Thermocycling was performed as follows: 95°C for 2 min, followed by 30 cycles of [95°C for 30 sec + 58°C for 30 sec + 72°C for 2 min] and a final extension at 72°C for 10 min. We digested the product with DpnI (NEB) for 1 hour and purified it using columns (Macherey-Nagel). We put the mutagenized genes in a pIVEX vector, previously amplified with primers that incorporated a random barcode downstream the STOP codon (aattccagcacactggcDHVBDHVBDHVBDHVBDHVBDHVBDHVBDHVBaagcccgaaaggaagctgag and ttaaagttaaacaaaattatttctagagggaaaccgttg). Previously a SalI site was added into the pIVEX vector, upstream the T7 promoter. We used NEB Hi-Fi DNA assembly using 100ng of vector DNA, 103 ng of insert DNA, in a 2:1 insert:vector molar ratio and incubated for 15 min at 50°C. We purified and concentrated DNA with a Zymo Research kit. We transformed the product into chemocompetent T7 Express lysY/Iq bacteria (NEB C3013I) via heat shock and spread them on a Petri dish with LB and

 17

Ampicillin. We incubated overnight at 37C and obtained around 1000 CFU. We grew the library overnight at 37C in liquid LB with Ampicillin and extracted the plasmid using Macheray Nagel kit. We digested the library plasmid with EcoRI-HF and SAlI-HF from NEB, in rCutSmart buffer at 37C for 1h40min plus an inactivation step og 65C for 20’. We added proteinase K and incubated at 37C for 15 min. We gel purified the sample to keep the fragment containing the KlenTaq gene, using Zymo research kit for concentration and purification of the DNA. We dialysed our sample using millipore membranes for 1h. For nanopore oxford sequencing we used a flongle (version FLO-FLG001), and SQK-LSK110 kit for sample preparation. We started with 110 fmol and loaded 50 fmol into the flow cell.

minION reads pre-processing

minION raw data was basecalled using ONT Guppy version 5.1 using model dna_r9.4.1_450bps_sup.cfg. We kept reads which had a length of 1700 to 2100 nucleotides and a mean Qscore value larger than 10 (except for the large library, for which we used a Qscore cut-off of 15). For the wild type and the seven variants we also used guppy for demultiplexing. For the KlenTaq large library, we used a custom script made in the lab and grouped barcodes by exact match. Sequences were aligned to the reference wild type using minimap2 version 2.21 [15], using the options minimap2 -ax map-ont --sam-hit-only, and separately for forward and reverse reads by adding --for-only / --rev-only. We used samtools 1.7 [16] to count occurrences of nucleotides per position, using the three consecutive commands: samtools view -S -b , samtools sort , samtools mpileup -Q 0.

Consensus by Nanopolish

We used the scripts provided by Oxford Nanopore, multi_to_single_fast5 and single_to_multi_fast5 (version 4.0) to split fast5 files and reassemble them according to the associated barcode. We then used the commands nanopolish index ; samtools sort; samtools index; and nanopolish variants --consensus, according to the Nanopolish manual, to compute the VCS. Our nanopolish version is 0.13.3.

Consensus by Medaka

We used racon (version 1.4) to polish our sequences and used it as an input in the consensus computation by medaka (version 1.4) using the command line medaka_consensus with default

18

parameters.

Availability

Project name: SINGLe

Project home page: https://github.com/rocioespci/single.

Operating system: Linux

Programming language: R

License: MIT

Funding

This project has received funding from the European Union’s Horizon 2020 research and innovation program under the Marie Sklodowska-Curie grant agreement No 845976 and from the European Research Council (ERC, Consolidator Grant No. 647275 ProFF).

Competing interests

The authors declare that they have no competing interests.

List of abbreviations

ePCR: error-prone PCR

PCR: polymerase chain reaction

SINGLe: SNPs In Nanopore reads of Gene Libraries

SNP: single nucleotide polymorphisms

Qscore: Quality score

VCS: Variant consensus sequence

Sk: Skewness

Authors' contributions

RE and YR designed research and conceptualization. YR performed supervision of the

 19

research. RE, and ADM performed experimental research. RE, NK and YR perform methodology and formal analysis. RE wrote the software. RE and YR wrote the manuscript, ADM and NK review and edited the manuscript.

References

1. Oxford Nanopore Technologies. https://nanoporetech.com/

2. Wang Y, Zhao Y, Bollas A, Wang Y, Au KF. Nanopore sequencing technology, bioinformatics and applications. Nature Biotechnology. 2021. pp. 1348–1365. doi:10.1038/s41587-021-01108-x

3. Sedlazeck FJ, Rescheneder P, Smolka M, Fang H, Nattestad M, von Haeseler A, et al. Accurate detection of complex structural variations using single-molecule sequencing. Nat Methods. 2018;15: 461–468.

4. Gong L, Wong C-H, Cheng W-C, Tjong H, Menghi F, Ngan CY, et al. Picky comprehensively detects high-resolution structural variants in nanopore long reads. Nat Methods. 2018;15: 455–460.

5. Loman NJ, Quick J, Simpson JT. A complete bacterial genome assembled de novo using only nanopore sequencing data. Nat Methods. 2015;12: 733–735.

6. Vaser R, Sović I, Nagarajan N, Šikić M. Fast and accurate de novo genome assembly from long uncorrected reads. Genome Res. 2017;27: 737–746.

7. Medaka. https://nanoporetech.github.io/medaka

8. Chenhao Li, Kern Rei Chng, Esther Jia Hui Boey, Amanda Hui Qi Ng, Andreas Wilm, and Niranjan Nagara- jan. Incseq: accurate single molecule reads using nanopore sequencing. Gigascience. 2016;5: s13742–016.

9. Karst SM, Ziels RM, Kirkegaard RH, Sørensen EA, McDonald D, Zhu Q, et al. High-accuracy long-read amplicon sequences using unique molecular identifiers with Nanopore or PacBio sequencing. Nat Methods. 2021;18: 165–169.

10. Sze MA, Schloss PD. The Impact of DNA Polymerase and Number of Rounds of Amplification in PCR on 16S rRNA Gene Sequence Data. mSphere. 2019;4. doi:10.1128/mSphere.00163-19

11. Zurek PJ, Knyphausen P, Neufeld K, Pushpanath A, Hollfelder F. UMI-linked consensus sequencing enables phylogenetic analysis of directed evolution. Nat Commun. 2020;11: 6023.

12. Yang KK, Wu Z, Arnold FH. Machine-learning-guided directed evolution for protein engineering. Nat Methods. 2019;16: 687–694.

13. Krishnakumar R, Sinha A, Bird SW, Jayamohan H, Edwards HS, Schoeniger JS, et al. Systematic and stochastic influences on the performance of the MinION nanopore sequencer across a range of nucleotide bias. Sci Rep. 2018;8: 3159.

14. Huang Y-T, Liu P-Y, Shih P-W. Homopolish: a method for the removal of systematic errors in nanopore sequencing by homologous polishing. Genome Biol. 2021;22: 95.

15. Li H. Minimap2: pairwise alignment for nucleotide sequences. Bioinformatics. 2018;34: 3094–3100.

16. Li H, Handsaker B, Wysoker A, Fennell T, Ruan J, Homer N, et al. The Sequence Alignment/Map format and SAMtools. Bioinformatics. 2009;25: 2078–2079.

Click here to access/download
Supplementary Material
SINGLe_Supplementary.pdf
