# Supplementary material for: Accurate gene consensus at low nanopore coverage
Source: Gigascience. 2022 Nov 9;11:giac102. doi: 10.1093/gigascience/giac102 (PMC9646519; doi:10.1093/gigascience/giac102)
Supplement: giac102_GIGA-D-22-00024_Revision_1 [file giac102_giga-d-22-00024_revision_1.pdf]

GigaScience

Accurate gene consensus at low nanopore coverage

--Manuscript Draft--

**Manuscript Number:**	GIGA-D-22-00024R1
**Full Title:**	Accurate gene consensus at low nanopore coverage
**Article Type:**	Technical Note
**Funding Information:**	H2020 European Research Council (845976) — PhD Rocio Espada<br>H2020 European Research Council (647275) — PhD Rondelez Yannick
**Abstract:**	Background Nanopore technologies allow high throughput sequencing of long strands of DNA at the cost of a relatively large error rate. This limits its use in the reading of amplicon libraries in which there are only a few mutations per variant and therefore they are easily confused with the sequencing noise. Consensus calling strategies reduce the error but sacrifice part of the throughput on reading typically 30 to 100 times each member of the library.<br>Findings In this work, we introduce SINGLe (SNPs In Nanopore reads of Gene Libraries), an error correction method to reduce the noise in nanopore reads of amplicons containing point variations. SINGLe exploits that in an amplicon library, all reads are very similar to a wild type sequence from which it is possible to experimentally characterise the position-specific systematic sequencing error pattern. Then, it uses this information to reweight the confidence given to nucleotides that do not match the wild type in individual variant reads, and incorporates it on the consensus calculation.<br>Conclusions We tested SINGLe in a mutagenic library of the KlenTaq polymerase gene, where the true mutation rate was below the sequencing noise. We observed that contrary to other methods, SINGLe compensates for the systematic errors made by the basecallers. Consequently, SINGLe converges to the true sequence using as little as 5 reads per variant, fewer than the other available methods.
**Corresponding Author:**	Rondelez Yannick, PhD<br>ESPCI Paris/CNRS/PSL<br>Paris, FRANCE
**Corresponding Author Secondary Information:**	
**Corresponding Author's Institution:**	ESPCI Paris/CNRS/PSL
**Corresponding Author's Secondary Institution:**	
**First Author:**	Rocio Espada
**First Author Secondary Information:**	
**Order of Authors:**	Rocio Espada<br>Adèle Dramé-Maigné<br>Nikola Zarevski<br>Rondelez Yannick, PhD
**Order of Authors Secondary Information:**	
**Response to Reviewers:**	Dear Dr Zauner,<br><br>I submit a corrected version of our manuscript, in which we have incorporated all the suggestions done by the reviewers. In particular, as requested, we have extended the explanation of the method, clarified the accessibility to the raw data and included the full code to reproduce the analysis in our git hub. Below, we address one by one the points raised by the reviewers.

Powered by Editorial Manager® and ProduXion Manager® from Aries Systems Corporation

Best wishes,
Yannick

We thank the reviewers for taking their time to carefully read our manuscript and provide valuable feedback. They have raised a number of interesting questions that we address below and that have been included in the reviewed version of our manuscript.

#-----------#

Reviewer #1: The paper "Accurate gene consensus at low nanopore coverage" describes a method SINGLe, which characterizes the position-specific systematic sequencing error pattern with logistic regression, and produces higher quality consensus calls with low coverage over a small region of highly covered area. SINGLe focuses on mutation calling using nanopore sequencing in a small region of interest on a genome. This type of tool is novel and can be useful for development for e.g. clinical tests or any other amplicon sequencing application. The coverage required to converge at a mutation call is smaller than other tools previously reported, which saves sequencing cost and/or gives more reliable results. Previous tools focus on genome assembly, while SINGle focused on mutation calling in small regions of interest on a genome.

Major feedback:
1 - The manuscript only demonstrates that the method works on one specific gene region. The authors should at least include an extra gene region to showcase that the method is robust regardless of the composition of the bases and motifs in that target region. At the moment, it is unclear if it is applicable to other regions as the authors suggested.

For this short manuscript, we limited the discussion to the KlenTaq gene used as an example This is to avoid overloading the manuscript and keep it at a reasonable length. However, we have used SINGLe in the NBI nicking enzyme gene (33% GC content) in our laboratory and the results are similar. In fact, since SINGLe applies a probabilistic approach on the erroneous nanopore reads, irrespective of what mechanism actually causes the systematic errors, we do not expect a strong dependence on gene properties. We have included this point in the discussion: “SINGLe needs to characterize the sequencing errors done on an appropriate reference sequence. Because it applies a probabilistic approach on the erroneous nanopore reads, irrespective of what mechanism actually causes the systematic errors, it should not depend on the genes’ properties. “

2 - The method could be more clearly described. For instance, there is mention of reweighting based on priors, but this is not further explained. The method is not very complex, but nevertheless it would be good to be explicit about these things.

We thank the reviewer for this suggestion and have extended the explanation of the method: “To adapt the classifier, an a priori expectation of mutations (p_{prior−right}) is needed, which must come from independent information. In the presented case, the variant sequences originated from an epPCR, for which we possessed an estimate of the mutation rate m_n→n’ given by the manufacturer [14]. We computed the a priori probability of observing nucleotide n’ as p_{prior−right} (n→n’)= m_n→n’ <m>/[Σn counts_reference(n) Σn’ m_n→n’], where <m> is the mean number of mutations expected in the library (reported by the epPCR kit manufacturer), and counts_reference(n) is how many times the nucleotide n is present in the wild type DNA strand. We also obtained the a priori expectation of an observed mismatch to be a sequencing error (p^{p,n}_prior−error) as the sequencing error rate at that position in the wild type set, independently of the Qscore. Using these values we computed for each strand, position (p) and nucleotide (n), the probability of observing a mutation with Qscore=Q as P^{p,n}_mutation (Q)= [counts^{p,n}(Q) /ΣQ’ counts^{p,n}(Q’)]p^{p,n}_prior−error where counts^{p,n} is the number of times the nucleotide n appears in position n across all the reads. Similarly, we computed the probability of observing a wild type nucleotide with Qscore=Q as P^{p,n}_wildtype(Q)=[counts^{p,wildtype}(Q)/ ΣQ’ counts^{p,wildtype}(Q’)]

Powered by Editorial Manager® and ProduXion Manager® from Aries Systems Corporation

p_prior−right(wildtype→n). Finally, we normalised N^{p,n}_correct (Q)= P^{p,n}_wildtype/[P^{p,n}_wildtype(Q)+ P^{p,n}_mutation(Q)]. This process shifts the logistic regression towards the higher Qscore, allowing the classifier to accept a number of observed mismatches consistent with the prior expectation (Figure 1D). The fits were done independently for each position and possible mismatched nucleotides.“

Moreover, the depth of the number of wildtype sequences needed for training is not clearly stated in the main text. It is crucial to know if other people want to apply the method to larger regions in the genome. (The author mentioned they used 5847 full length coverage of the wildtype but no discussion/ analysis was provided on whether this can be lower or what the lower limit is.)

We have added a discussion on this topic on the Supplementary Material (page S12), and refer to it in the main text: “ Please refer to the supplementary material for a brief discussion on ‘How many Nanopore reads are needed to fit SINGLe?’.”

3 - While the regressions are done strand specific, no analysis is done on e.g. strand bias, while it is known to be very important for accurate consensus calling. Are the results indeed different for the different strands?

Indeed, the patterns of errors in each position are different on the forward and reverse reads. We added a discussion on the supplementary material (page S13), and referred to it in the main text: “We also separated the fits for forward and reverse strands as the error rate per position is different in each case (see ‘Are reverse and forward reads different in Nanopore Sequencing’ in Supplementary material). ”. It is true that a better consensus is obtained by combining nanopore reads from both directions, but this is independent of SINGLe.

4 - Have the authors considered taking into account the observed neighbour correlation (Fig S2) in the prediction (i.e. by taking more than 1 feature into account in the logistic regression).

(Note that Fig S2 is now Fig S3) This is an interesting suggestion. This is one option that we have considered at the beginning of the project and that would be interesting to consider as an extension of our approach for higher mutational loads. In the current approach we assume that true mutations are scarce, and therefore that the neighbor pattern is always the wild type. It is under this assumption that we build one independent logistic regression for every position and every possible mismatch.

5 - It would make the work more valuable if more discussion around the potential applications of this method is provided. It is mentioned in the introduction Line: 72 that it can be used in an evolution experiment. The case demonstrated is ePCR introduced mutations on a single gene (KlenTaq Polymerase gene). Can the authors clarify the link between the two, or link to other clinical applications such as tumor gene sequencing?

Thanks for the suggestion. We have clarified that error prone PCR is the method used for generating gene libraries in directed evolution experiments: “For example, in directed evolution experiments the genetic libraries typically originate from a single ancestral sequence (the wild type) that has been submitted to limited randomization, for example using error-prone PCR (epPCR) [2]. ” We have also included as potential applications structural variants detection in cancer, with a reference: “Another example is the detection of structural variants in cancer cells [3].” in the introduction.

Minor feedback:
6 - Some figures can be enlarged. (e.g. Fig 3B).

We have increased the size and quality of our figures. In particular, we have changed the presentation of data in Fig. 3B to make it easier to interpret.

Powered by Editorial Manager® and ProduXion Manager® from Aries Systems Corporation

7 - The manuscript is submitted as a technical note type of paper. According to the reporting standards, the manuscript should contain a section on data availability and testing to enable readers to easily reproduce the results. However, the access to data is not clearly described in the main text. Testing procedures are also not provided in github as far as this reviewer can deduce.

Previously, this information was included in the availability section, but now it has been moved to "data availability" following GigaScience guidelines and the reviewer's suggestion. We have uploaded an R notebook including all the codes used for this study to our git-hub repository.

8 - Typo:Fig 3B xlabel: diference

We have corrected the typo.

9 - Typo: Line 266: "f"igure should be capital

We have replaced 'figure' by 'Figure'.

10 - Clarify Availability, Operating system (If it is a R package, I expected it to be cross platform. Or please report precise operation system (e.g. x86_64-pc-linux-gnu (64-bit)), Programming language: R (version x.x.x), Other requirements (missing). The instructions to use the program are minimal on github. Consider copying the vignettes to Github README:
https://github.com/rocioespci/single/blob/master/vignettes/single.Rmd

We have clarified the information on the availability requested by the reviewer and included the vignette to the git-hub readme file. SINGLe is now available in bioconductor, a repository for R packages related to biological assays. Therefore, it has been tested in different platforms (linux, windows, macOS), and this information has been included in the manuscript (availability section). The Github readme has been extended, including the information on the vignette, as suggested by the reviewer.

#-----------#

Reviewer #2: In the manuscript submitted by Rocio et al. the authors present a new method, SINGLe, to reduce noise in nanopore sequencing data in the scope of direct evolution experiments. In essence, SINGLe is a machine learning approach utilizing logistic regression trained on reference sequences to model the probability of true variants in random mutagenesis libraries (by error-prone PCR) sequenced using Oxford nanopore technology. In the results, the authors show that SINGLe shows a better performance than other approaches (like nanopolish or racon/medaka) with respect to the discrimination between correctly and incorrectly called SNPs and the number of reads necessary for building a correct consensus sequence of one variant. In summary, the submitted manuscript is a technical note that is relatively long but incomplete in terms of details on methodology and execution. Consequently, I have several major issues that will be listed in the following.

1) The structure of the manuscript could be enhanced from my point of perspective in several ways. First, the authors are not clearly separating descriptive methodology from the presented results. For instance, details about the applied library preparation, sequencing, and analysis protocols and pipelines are somewhat distributed over several sections, making it hard to follow the authors without hopping through the manuscript.
Also, the manuscript has in essence just two main sections, i.e., Findings and Methods. However, the Findings section contains also a Method subsection but at the same time also contains a Background subsection, which is clearly not a finding.

Powered by Editorial Manager® and ProduXion Manager® from Aries Systems Corporation

This article is submitted as a technical note and it follows GigaScience editorial guidelines. The Findings section includes the background, the methodology related to SINGLe (the new methods we are presenting in this work), and the results. On the other hand, the Methods section includes previously available methodologies that were used. To clarify this, we have changed the title to 'SINGLe method' inside the Findings section.

2) The writing could be enhanced. There are quite a lot of occasions of grammar errors or misspellings.

We have thoroughly reviewed the grammar and the spelling of the manuscript.

3; line 81 ff.) It is not clear what the data basis for the following observations are. Even though the overall statement of the correlation of quality scores and errors and the fact that nanopore errors are not evenly distributed is not to be questioned, from a strict scientific point of view it should be made clear how this was observed, precisely on what data.

We specified the sample we used for this analysis "We used a nanopore sequencer to read 5847 strands of the wild type KlenTaq gene (length 1662 nucleotides), for which we have a ground truth sequence obtained by Sanger sequencing (supplementary Sequence S1). In this data set, we can confidently attribute mismatches between the read sequences and the known wild type as sequencing errors, and matches as correct reads."

4; line 82 f. and Figure 1A) As pointed out in the manuscript, the q-scores are directly related to the error rate, i.e. a low q-score indicates an erroneous base call. However, (the reference to) Figure 1A does not make this so obvious as it sounds in the text, as the figure is not normalised with respect to total counts. In fact, the total numbers of correct/false base calls is irrelevant to make this point, it is more about the reported error rate (the q-scores) vs the observed error rate (also in terms of q-scores). Something like a qq-plot would be more appropriate to make this point.

The first affirmation is only partially true. While it is true that errors tend to have a low Qscore, there are also plenty of correct reads that have a low Qscore, as shown by the lower peak of the green distribution on figure 1A. Thus, a low q-score does not necessarily indicate an erroneous base call. The aim of figure 1A is to make this point clear. So we have clarified: "The Qscore assigned to each nucleotide tends to be low when a wrong nucleotide is assigned, as expected. Notice that the inverse is not true: some low Qscores correspond to correctly basecalled bases, so both distributions overlap. Thus, a simple classification based on the Qscores is not possible to distinguish signal from noise. "
We chose not to show the normalized distributions in the main text because the peak on the errors distribution is higher than the correct reads. To the distracted reader, it could look that nanopore sequencers make a higher proportion of errors than correct reads and this is not true. We have included the normalized distribution in the supplementary material (Figure S1).

5; line 96 ff. and line 110) Where were the 6000 KlenTaq reference sequences obtained from? The later Methods section only details about the small and large variant library, not about the wild type library.

We agree that the information was not properly stated. The reference KlenTaq was measured together with the seven clones. The overall experimental procedure is similar, and it has now been specified in the Methods.

Is there any kind of PCR involved here, and if so, how do the authors separate between sequencing errors or other errors sources (because, for instance, an error introduced during PCR when amplifying a specific gene is - if sequenced correctly - not a sequencing error even if the generated read mismatches with the reference)?

Powered by Editorial Manager® and ProduXion Manager® from Aries Systems Corporation

This is indeed true: if there was a base substitution during PCR a mismatch with the reference would not mean a sequencing error. To reduce the possibility of mutations during PCR, we used Q5 high fidelity polymerase (NEB) for amplification of the gene prior to nanopore sequencing, following the suggested conditions. This method has an extremely low error rate during replication: 10-7 substitutions per base per doubling [Potapov V, Ong JL (2017) https://doi.org/10.1371/journal.pone.0169774], which is 6 order of magnitudes lower than the Nanopore sequencing error rate. Taking into account these considerations, we neglected the substitution occuring during the PCR amplification. In addition one may note that if the library to be SINGLed is prepared following the same library preparation protocol as the reference (for example, as a pool), then the error introduced at the molecular level, if systematic, would be included in the SINGLe correction.

6; line 105 and ff.) The abbreviation ePCR for error-prone PCR is very unusual. To the best of my knowledge, epPCR is the established short term for error-prone PCR and I would advise in using it instead of ePCR.

We replaced “ePCR” by “epPCR” in the manuscript.

7; line 106 ff.) The two sentences beginning with 'In this range …' are difficult to read and should be revised. Also, the sequencer does not report a mismatch nor a wild type or a mutation, it just reports a nucleotide that can either be correct or not in comparison to a specified reference.

The text has been modified and now it reads: ”For nucleotides read as wild type their Qscore are directly transformed into a p_right according to the Q values reported by Guppy: p_Guppy=1-10-Qscore/10.”. We have also removed the expression “the sequencer reports a mismatch/wildtype”.

8; line 123 ff.) What type of logistic regression was used, what is the underlying distribution? How was the model evaluated, for goodness of fit (over- underfit)?

We used a binomial model for the logistic regression. We have included this information in the main text: “We fitted this relation by a logistic regression using a binomial model”. We have also added in the Supplementary material an evaluation of the goodness of fit calculated as 1- deviance of null model / deviance of model and discussed the implications on SINGLe fits (page S12).

9; line 130 ff.) How well does the 'estimation of the mutation frequency' of the ePCR that the authors possess reflect the actual mutation rate (see also later comments)? Is there other literature on that issue?

We verified the mean number of mutations in the consensus sequences obtained after analysis of the large library: 8.4 mutations / kb. This value is close to the estimated value by the Mutenzyme II kit (8 mutations/kb), the commercial kit we used for error prone PCR.

10; line 131 ff.) How is the a priori sequencing error rate defined and what is it based on? And how was the re-weighting of the wild type fit carried out in detail?

We have extended the description of the method on the main text to clarify these points: “To adapt the classifier, an a priori expectation of mutations (p_{prior−right}) is needed, which must come from independent information. In the presented case, the variant sequences originated from an epPCR, for which we possessed an estimate of the mutation rate m_n→n’ given by the manufacturer [14]. We computed the a priori probability of observing nucleotide n’ as p_{prior−right} (n→n’)= m_n→n’ <m>/[Σn counts_reference(n) Σn’ m_n→n’], where <m> is the mean number of mutations expected in the library (reported by the epPCR kit manufacturer), and

counts_reference(n) is how many times the nucleotide n is present in the wild type DNA strand. We also obtained the a priori expectation of an observed mismatch to be a sequencing error ($p^{p,n}_{prior-error}$) as the sequencing error rate at that position in the wild type set, independently of the Qscore. Using these values we computed for each strand, position (p) and nucleotide (n), the probability of observing a mutation with Qscore=Q as $P^{p,n}_{mutation}(Q) = [counts^{p,n}(Q) / \Sigma_{Q'} counts^{p,n}(Q')] p^{p,n}_{prior-error}$ where $counts^{p,n}$ is the number of times the nucleotide n appears in position n across all the reads. Similarly, we computed the probability of observing a wild type nucleotide with Qscore=Q as $P^{p,n}_{wildtype}(Q)=[counts^{p,wildtype}(Q) / \Sigma_{Q'} counts^{p,wildtype}(Q')] p_{prior-right}(wildtype \rightarrow n)$. Finally, we normalised $N^{p,n}_{correct}(Q) = P^{p,n}_{wildtype} / [P^{p,n}_{wildtype}(Q) + P^{p,n}_{mutation}(Q)]$. This process shifts the logistic regression towards the higher Qscore, allowing the classifier to accept a number of observed mismatches consistent with the prior expectation (Figure 1D). The fits were done independently for each position and possible mismatched nucleotides. ”

11; line 137 ff.) In this paragraph, the authors explain how they have inferred q-scores for nucleotide positions that were deleted in the mutant type read set. First of all, what does 'fixed to the minimum Qscores of their nearest neighbors' mean precisely? To the minimum of the direct two 5' and 3' neighboring nucleotides or over a window?

To the minimum of the direct two 5' and 3' neighboring nucleotides, not over a window. We have clarified in the main text: “we fixed their confidence value as the minimum of the Qscore of their direct nearest neighbors in the nucleotide sequence.”

Second, while there is indeed an obvious correlation of q-scores of neighboring nucleotides in a sequencing read, in my opinion this is for this application an oversimplification, as it is biased towards correctly called bases (which clearly make up the majority). As the authors have already pointed out, erroneous base calls tend to have lower quality scores and they do occur all over the read. Then, how well do q-scores of these erroneous positions correlate with their neighbors? A deletion event introduced during sequencing is also an error and should be in line with or within range of q-scores of erroneous base calls.

We have included panel C in figure S3 (previously Fig S2) to address this question. We plotted the distribution of difference in the Qscore (reported by guppy basecaller) for a nucleotide and its closest neighbor on the 3’ side, classified by being an error or a correct read. In both cases, most nucleotides have a small difference of Qscore with its neighbor, and the difference is smaller for errors. As a control, we showed this is not the case for random pairs of nucleotides. This supports that for any read, and in particular for errors, there is some correlation between the Qscore of one nucleotide and its neighbors.

And why were insertions ignored altogether?

It is mainly a methodological constraint. Insertions can be very diverse (they do not have a defined length), and it is hard to accumulate enough data to fit them. For each position and possible insertion, we would need a few appearances by error in the wild type sequence to fit them. While the current version of SINGLe ignores them all together, the corrected confidence values for no-insertion positions and the original Qscores assigned to the insertions could be used in other methods to compute the consensus (for example, samtools) and decide whether a insertion is true or is a sequencing error.

Finally, we conceived SINGLe for gene libraries using error prone PCR. In these cases, insertions are rare events (less than 1% of the mutations according to the kit’s manufacturer).

We have added to the text: “ Insertions were ignored as very few are expected (<1%) and it is not possible to obtain enough reads to fit all insertion possibilities. In applications where SINGLe is used to compute a high quality consensus, the original score of the inserted bases can be carried over. “

12; line 173) How many variants were sequenced exactly? This should be known as each library was tagged with an individual barcode.

We have included this information in the section Samples preparation and sequencing:

Powered by Editorial Manager® and ProduXion Manager® from Aries Systems Corporation

“After data pre-processing (see section minION reads pre-processing), we had the following reads per variant: 716 (variant 1), 453 (variant 2), 645 (variant 3), 428 (variant 4), 422 (variant 5), 369 (variant 6), 358 (variant 7).”

13; line 189 ff.) If the threshold is zero, i.e. the cutoff on p_right and p_guppy is zero, signal is defined as mismatches of know mutations, no matter the of actual q-score or p_right, and noise as mismatches of non-mutated positions. The actual nucleotide sequence is equal for both cases. How can it be that the signal to noise ratio is different for guppy and SINGLe?

The difference arises in the method for counting. The weighted count of true positives (signal) is not the number of correct nucleotides, but the sum of the p_right/p_guppy for those nucleotides. Because SINGLe reduces the p_right for the errors (false positives) in comparison to the scores assigned by Guppy, the ratio signal to noise is higher, even when a cut-off 0 is used.
In figure S5 (previously fig S3), in which the counts are not weighted, the ratio signal to noise for SINGLe and Guppy matches at cut off zero, as the reviewer points out.
We have updated the paragraph to clarify this point: “Notice that the ratio is different for both methods at cutoff zero because the counts are weighted by p_right.”

14; line 191 ff.) How were the counts weighted by p_right exactly. In this regard, the signal to noise ratio seems to be equal despite weighting being applied or not (comparison of Fig2 and supplementary Fig3), so what is the actual methodological difference here?

(Note that Fig S3 is now Fig S5).
This observation was answered in the previous comment and we have also clarified how the counts are weighted by p_right in the main text: “The counts are weighted by p_right for each nucleotide, i.e. instead of summing one for each occurrence, we summed the p_right associated with the nucleotide.”
While the general shape of the figure is similar, as pointed out by the reviewer in the previous comment (#13), when using the weighted values both methods differ, while this is not true for the regular counts. We decided to show Figure 2 with weighted counts on the main text because it shows how the weights increase the scores (i.e. the p_right values) of the correct reads and reduce those of the incorrect reads.

15; line 194 ff.) This paragraph is somewhat imprecise and lacks scientific references on how consensus calling and polishing of nanopore reads is done on a broad scale. For instance, nanopolish uses the raw signals of the nanopore sequencer (fast5) in combination with HMMs to detect base variations, which are then filtered and eventually combined.

We modified the text to explain how consensus is done by other methods, including references: “We also computed the consensus using Nanopolish, which works directly on the raw electrical signal instead of the basecalled sequences in combination with HMMs to detect base variations [7], Medaka that counts nucleotides and uses a neural network to compare to a draft assembly and define mutations [9], and NextPolish which takes into account the neighbours of the nucleotide [15].“

16; line 197 ff.) As far as I understood SINGLe, it essentially transforms the q-scores into a probability score. But how is that now incorporated into a consensus calling process? All the methodological details on this are missing in the manuscript. This also applies to the p_guppy and 'no weights' consensus pipeline. And what is the difference between p_guppy and 'no weights'?

The reviewer has correctly understood that SINGLe transforms the q-scores to a probability score. These probabilities are then used to weight the counts of occurrence of nucleotides used in consensus calling. We have clarified this point: “We compared the consensus sequences of the 7 KlenTaq variants obtained by SINGLe and by other methods. Given several aligned nanopore reads of the same variant, the consensus

Powered by Editorial Manager® and ProduXion Manager® from Aries Systems Corporation

can be computed by simple majority counting how many times each nucleotide was read in one position and keeping the most frequent. We call this method ‘no weights’. The counts can also be weighted by p_Guppy and choose the nucleotide with larger weighted count. We call this method ‘Guppy’. Or the counts can be weighted by p_SINGLe (‘SINGLe’ method). “

17; line 203 ff.) The consensus calling was done for each of the seven variants independently, so seven times 47 times 50 sets. I would recommend to be precise here in the manuscript.

We have clarified: “We computed the consensus independently for each variant using subsets of 3 to 50 sequences drawn randomly from all available reads, and repeated 50 times for each subset size.”.

18; line 208 ff. and Fig 2B) Why did nanopolish failed on the #1 dataset? Given the results on the other variants this seems to be highly unlikely.

Variant #1 has two mutations: G23A and C245T. Nanopolish fails systematically to find G23A.

19; line 223 ff. And Fig 2D) Based on the figure it can not be concluded that nanopolish misses mutations instead of reporting false positives, as false negatives are not considered or measured in this manuscript. This even more highlights that sensitivity (recall) is an important aspect, especially in the field of variant detection. So what is the false negative rate or the sensitivity? The whole evaluation is more about precision, but precision often comes at the price of sensitivity and finding low abundant / rare variants is of high interest for many applications. Why not defining TP=mismatches of known mutations above p_right, FP=incorrect mismatches above p_right, TN=incorrect mismatches below p_right, and FN=mismatches of known mutation below p_right and presenting also incorporating evaluation statistics like F1 scores?

Following the reviewer's suggestion, we updated Figure S6 (previously, Fig S4) to show not only all detected mutations but also TP (true mutations), FP (false mutations), TN (true wild types) and FN (false wild type) nucleotides in the consensus obtained from subsets of nanopore reads, plus the sensitivity and the specificity.
We have also updated this paragraph in the main text to make it more accurate: “Figure 2C shows the total number of mismatches (averaged over the 50 trials) reported by each method for variant #3, according to the number of reads used to compute the consensus. For any set size, SINGLe reports the closest number of true mutations compared to the other methods. In Figure S6 this is analyzed in more detail: we classified the nucleotides in each consensus sequence according to true/false mutations and true/false wild types and observed that actually SINGLe detects true mutations with the fewest number of reads while keeping the lowest rate of false mutations. Nanopolish has a high rate of false wild types and underestimates the true mutations. Medaka has a similar behavior and on top adds false mutations. Consensus using Guppy scores or no weight report a high number of false mutations.”

20; paragraph line 242) The way the authors have compared the results of the large library with that of the seven known variants is, at its best, only partially confirming the results of the latter one. First, why was medaka favoured over nanopolish if nanopolish showed clearly the better performance on the seven variants dataset?

We compared our method against Medaka in the main text because it is the method suggested by Oxford Nanopore and the most widely used. Nevertheless, comparison to other methods, including Nanopolish, are also reported in the supplementary material.

Given the related results in the supplement, nanopolish failed this time on the majority of the shown datasets. Why? What are these variants and how many actual mutations do they have, is there a pattern?

Powered by Editorial Manager® and ProduXion Manager® from Aries Systems Corporation

	The reviewer is right and actually we found a mistake on the parsing of the mutations output by Nanopolish. We have now corrected (and double checked) this and the results have improved. Nevertheless, Nanopolish has a poor performance in some examples. The sequences of these variants are listed in table S2. In most cases, Nanopolish makes one or two mistakes in the sequence, but we do not find any pattern that relates them. Here we enumerate the errors:<br>For barcode AAATTACGAAACTTAGGTGGAAATGCAGATATTAG, Nanopolish predicts two extra substitutions (A1548G and G633A) in the consensus done by subsets of reads compared to consensus computed with all available sequences.<br>For barcode ACGCGCACGTAGACAGATACGAAGGCCGACAGGAG, predicts an extra substitution (C592T) in the consensus done by subsets of reads compared to consensus computed with all available sequences.<br>For barcode GAGTTAAGGTGCAAGGGTATATGTTCCCATGTG the deletion A1152- is missed in the consensus done by subsets of reads compared to consensus computed with all available sequences.<br>For barcode GTCCATGTAAGCACAGGTCCTACTTAAGATGTTAG, it predicts an extra substitution A957G in the consensus done by subsets of reads compared to consensus computed with all available sequences.<br><br>Second and above all, the way the ground truth is calculated is unclear. Was this done for each of the barcodes sets or over all reads?<br><br>The consensus was computed for the reads associated to each barcode independently. We clarified this point in the main text: "We sequenced the library using nanopore, grouped the reads according to the barcode and computed the consensus sequence for the reads associated to each barcode.".<br>If the former was the case, how does it comes that raw reads have an inferior performance (see supplemental Figure S6).<br>In figure S6 (now S7) 'raw' was accounting for the consensus computed with 'no weights'. We have corrected the legend of this figure. The worse performance is consistent with the observations of the first 7 mutants analyzed in the previous section.<br>If the latter was the case, a consensus over all of the 1200 variants would most probably be skewed towards the most present variants or towards the wild type, given the fact that epPCR should introduce errors evenly and at a random rate. In this sense, this read based consensus ground truth is biased towards high precision but low recall if the consensus ground truth harbors less mutations than the actual ground truth, as this would result in not calling a mutation (high precision but low recall) being better than calling a wrong mutation (low precision but high recall).<br>This is correct, that is why we computed the consensus for the reads associated to each barcode independently.<br>Finally, in this case SINGLe does not converge after 5 reads but after 10 reads, as there is a significant drop right after 5 reads.<br>We have re-written this sentence in the main text: "As shown in Figure 3A, SINGLe returns the correct consensus sequence over 90% of the times when using at least 5 reads, while Medaka needs 15."<br><br><br>21; line 253 ff. and Fig 3B) This is hard to read and understand, as the figure shows no percentages but they are discussed likewise in the manuscript. Also, the figure legend is missing essential information (e.g. what the color coding exactly means).<br><br>We have edited Figure 3B, and the text: "We computed consensus sequences using SINGLe or Medaka for all the mutants in our library, provided that the identifying barcode is present at least four times in our dataset, and compared the number of mutations reported by both methods (Figure 3B). Out of the 1174 variants for which we had at least 10 reads, both methods report the same consensus in 1039 cases (89%). Among the 610 variants for which we had 6 to 10 reads, we obtained the same consensus sequence using Medaka or SINGLe only in 339 variants (56%), and for the 839 variants with less than 5 reads only 23% of the variants obtained the same consensus with both methods. For the other variants, independently of the number of available reads, Medaka returns more mutations than SINGLe in the wide majority of cases. This is consistent with the observation in the previous section: Medaka tends to predict more mutations than there actually are. Less than 1% of all computed consensuses have more mutations by SINGLe than by Medaka."

Powered by Editorial Manager® and ProduXion Manager® from Aries Systems Corporation

22; line 264) I tend to disagree, but SINGLe does not correct systematic errors. It may reduce systematic errors, but it does not, as far as it is described, specifically correct (newly) known systematic errors, as otherwise the type and nature of the systematic error along with the appropriate measurements would have been presented.

We replaced this line by: “If SINGLe improves the consensus sequences by reducing systematic errors from the basecalling, then the mutations predicted for a randomly mutated gene library should be homogeneously distributed along the sequence. ”

23; line 266 ff.) What is the exact data basis here? How many datasets are below five, between 6 and ten, and above ten read counts, and how are they distributed?

There are 1174 variants with more than 10 reads, 610 with 6 to 10 reads, and 839 with less than five reads. We have incorporated this information, as cited in the response of comment #21.

24; line 311 ff.) SINGLe needs optimally the true reference sequence and the expected mutation rate, which both could also be obtained by other means, e.g. using high accuracy short read sequencing, as the authors have pointed out. However, this is in contradiction to the highlighted advantages of this approach presented here, i.e., low laboratory effort at high sequencing throughput. Have such applications being conceived or carried out to give a better understanding of the practicability of using SINGLe for such cases?

One example in which SINGLe can be helpful is directed evolution experiments. Generally, these start from a known wild type DNA sequence coding for a protein whose activity is to be enhanced. This wild type sequence is obtained by Sanger sequencing and it is used for the general experimental design, so it is not an extra constraint added by SINGLe. To proceed, copies of this gene are made by PCR adding random mutations on the gene. Today, commercial kits are available and the mean number of mutations on each variant is provided (and adjusted by the experimental conditions). Finally, SINGLe requires several reads of the wild type gene to characterize the error rate. This is the unique experimental effort that SINGLe implies. Because it is a unique wildtype gene, enough DNA material can be obtained by PCR and purification, and added to the sequencing run using Oxford Nanopore’s pipeline for multiplexing of samples. In return, as SINGLe lowers the number of duplicated reads for each variant from ~20 to ~5, the number of variants in a single sequencing run is multiplied by four, allowing to study larger gene libraries.

25) If I understand the methods correct, the same epPCR protocol was applied to the small and large library, but with a different cycle number (25 vs. 30), resulting in different mutation rates (3 muts/kb for the small and 8muts/kb for the large library). Why was a different rate chosen to begin with?

There is no particular reason for the different mutation rate. They are two independent experiments. Nevertheless, the high mutation rate for the large library (given by more cycles of PCR in error prone conditions) reduces the possibility of having wild type genes in the library for the downstream protocol. This is convenient as we aimed to find mutations in the gene. On the other hand, that is not a problem for the small library because the clones are individually chosen and their (non-wild type) sequence confirmed by Sanger sequencing.

Supplemental Table S2 shows the actual variants of the small library. Here, the first variant only shows two substitutions, which corresponds to a rate of 1.2muts/kb, whereas on the high end the variants show 4.7muts/kb. Is this the expected deviation (here about 60%), and if so, does this also apply to the large library, and if so, how well is this captured in the different datasets of the large library?

The consensus sequences obtained with SINGLe in the large library, for variants with more than 5 reads, contain between 1 and 30 mutations (thus 0.6 to 18 mutations/kb).

Powered by Editorial Manager® and ProduXion Manager® from Aries Systems Corporation

This deviation is in line with previous studies on the mutational profile of error prone PCR libraries (see for example Drummond, D. A., Iverson, B. L., Georgiou, G., & Arnold, F. H. (2005). Why high-error-rate random mutagenesis libraries are enriched in functional and improved proteins. Journal of molecular biology, 350(4), 806-816. doi:10.1016/j.jmb.2005.05.023).
How well do the different tools perform for the different types of variants (in terms of number of mutations but also substitutions versus indels)?
We compared the percentage of each type of mutation detected in the consensus sequences vs the values reported by the manufacturer's kit. We splitted the analyses for the variants according to the number of available reads (up to 5 reads, 6 to 10, more than 10), and by the method used to compute the consensus. We plotted the results in Fig S9, and computed the correlation between the observed values and the reported ones.
For all methods except Nanopolish, the correlation is the highest (.94) when looking at the variants with more than 10 reads. But when lowering the number of available reads, SINGLe outperforms the other methods.

This is important, as epPCR also have shown to be biased to some extent, favoring specific mutations over others, so introducing systematic errors of its own (see, for instance, Vanhercke et. al 2005).

Yes, it is well known that error prone PCR mutations are biased and these biases are indicated in the manual of the kit used (available in https://www.agilent.com/cs/library/usermanuals/Public/200550.pdf).

26) What was the decision of the authors to only include nanopolish racon/medaka for their evaluation? These tools have originally been developed to correct genome assemblies based on signal or read information, with nanopolish being considered as almost outdated by now. Other methods have been proposed to specifically correct for systematic errors in nanopore data (e.g. Homopolish, Nextpolish, NanoReviser). Why those newer tools have not been included for the evaluation?

As we discuss in the main text, most tools for nanopore sequencing are intended for de novo genome assembly rather than detecting few mutations in a well known sequence. Originally we had included Medaka because currently it is the standard for detecting mutations in nanopore sequencing reads and it is the software suggested by Oxford Nanopore, the company that provides the sequencer. We had also included Nanopolish which is less used currently because it takes a longer computation time, but it has been reported to be a little more accurate than Medaka (see for example Lee, Jin Young, et al. "Comparative evaluation of Nanopore polishing tools for microbial genome assembly and polishing strategies for downstream analysis." Scientific Reports 11.1 (2021): 1-11.).
Homopolish is designed for polishing genomes, a different use of nanopore sequencing that the one we propose in this article. Homopolish makes use of the available homologs sequences for correcting the reads, forcing natural occurring sequences. This is not the aim for reading DNA strands with synthetic mutations added in the lab.
NanoReviser is a method to improve basecalling using the raw electrical signal plus the predictions already done by other basecallers. This software depends on Albacore, an old basecaller from Oxford Nanopore which is not accessible anymore and that has been replaced by Guppy. We could not install NanoReviser to compare.
We have included in our manuscript NextPolish among the methods tested using KlenTaq's seven variants. Results are included in figures 2 and S6, and in the main text: "We also computed the consensus using (...) NextPolish which takes into account the neighbors of the nucleotide [15]." Also: "Consensus using Guppy scores or no weight report a high number of false mutations, and so does NextPolish (though with a lower error rate)." In the Discussion: "This implies a faster convergence than other methods currently used: nanopolish requires 8 to 15 reads, Medaka needs at least 20-50 reads and NextPolish between 15 and 50 reads to achieve a similar performance. " In Methods: "Consensus by NextPolish. We used nextPolish (version 1.4.1) with options task=best, rerun=3, genome= KlenTaq wild type sequence, and lgs_options -min_read_len 1k -max_depth 100."

Powered by Editorial Manager® and ProduXion Manager® from Aries Systems Corporation

	Figures:<br>27; Figure1B) It should be made clear that only a specific window of the whole gene was selected here (in contrast to the supplement).<br><br>We have clarified in the legend of figure 1 “Only positions 100 to 150 are shown here. An equivalent plot for all positions is available in Figure S2.”<br><br>28; Figure1C/D) The legend states that a black dot is the proportion of correct reads against the q-scores. A sequencing read does have as many q-scores as it has nucleotide positions. How was this averaged or is a black dot actually the proportion over a single base position?<br><br>It is the proportion over a single base position. We have clarified it in the text: “Black dots are the proportion of correct nucleotides with a given Qscore in one position and in comparison to one possible error.”<br><br>29; Figure 2 and 3) The legends are too long. A legend should not contain any interpretation of the shown data but only all information necessary to fully understand the figure.<br><br>We have reduced the length of the legends.<br><br>30; Supplementary Table S1) This is not a table.<br><br>We changed the name “Table S1” to “Sequence S1”.<br><br>31; all Figures) I would suggest to provide higher resolution graphics. Overall, the depicted information is hard to grasp visually. I would rather separate some of the sub-graphics into individual figures but make them larger in size instead.<br><br>We have increased the figures resolution.
**Additional Information:**	
**Question**	**Response**
Are you submitting this manuscript to a special series or article collection?	No
**Experimental design and statistics**<br><br>Full details of the experimental design and statistical methods used should be given in the Methods section, as detailed in our Minimum Standards Reporting Checklist. Information essential to interpreting the data presented should be made available in the figure legends.<br><br>Have you included all the information requested in your manuscript?	Yes
**Resources**	Yes

Powered by Editorial Manager® and ProduXion Manager® from Aries Systems Corporation

A description of all resources used, including antibodies, cell lines, animals and software tools, with enough information to allow them to be uniquely identified, should be included in the Methods section. Authors are strongly encouraged to cite Research Resource Identifiers (RRIDs) for antibodies, model organisms and tools, where possible.<br><br>Have you included the information requested as detailed in our Minimum Standards Reporting Checklist?	
**Availability of data and materials**<br><br>All datasets and code on which the conclusions of the paper rely must be either included in your submission or deposited in publicly available repositories (where available and ethically appropriate), referencing such data using a unique identifier in the references and in the "Availability of Data and Materials" section of your manuscript.<br><br>Have you have met the above requirement as detailed in our Minimum Standards Reporting Checklist?	Yes

Powered by Editorial Manager® and ProduXion Manager® from Aries Systems Corporation

Manuscript

Click here to access/download;Manuscript;SINGLe_Main_reviewed.pdf

Title page

Title:

Accurate gene consensus at low nanopore coverage

Authors:

Espada Rocío.

Gulliver Lab, ESPCI Paris, PSL University, CNRS, 75005 Paris, France.

Contact: rocio.espada@espci.fr.

ORCID 0000-0003-3829-473X

Zarevski Nikola. Gulliver,

Gulliver Lab, ESPCI Paris, PSL University, CNRS, 75005 Paris, France.

Contact: niko.zarevski@gmail.com

ORCID 0000-0001-5045-2425

Dramé-Maigné Adèle.

Gulliver Lab, ESPCI Paris, PSL University, CNRS, 75005 Paris, France.

Contact: docdeldou@gmail.com

ORCID 0000-0003-3586-0361

Rondelez Yannick.

Gulliver Lab, ESPCI Paris, PSL University, CNRS, 75005 Paris, France.

Contact: yannick.rondelez@espci.fr.

ORCID 0000-0002-2565-476X

Corresponding author

Abstract

Background Nanopore technologies allow high throughput sequencing of long strands of DNA at the cost of a relatively large error rate. This limits its use in the reading of amplicon libraries in which there are only a few mutations per variant and therefore they are easily confused with the sequencing noise. Consensus calling strategies reduce the error but sacrifice part of the throughput on reading typically 30 to 100 times each member of the library.

Findings In this work, we introduce SINGLe (SNPs In Nanopore reads of Gene Libraries), an error correction method to reduce the noise in nanopore reads of amplicons containing point variations. SINGLe exploits that in an amplicon library, all reads are very similar to a wild type sequence from which it is possible to experimentally characterise the position-specific systematic sequencing error pattern. Then, it uses this information to reweight the confidence given to nucleotides that do not match the wild type in individual variant reads, and incorporates it on the consensus calculation.

Conclusions We tested SINGLe in a mutagenic library of the KlenTaq polymerase gene, where the true mutation rate was below the sequencing noise. We observed that contrary to other methods, SINGLe compensates for the systematic errors made by the basecallers. Consequently, SINGLe converges to the true sequence using as little as 5 reads per variant, fewer than the other available methods.

Keywords:

Nanopore sequencing; consensus sequence; low coverage; gene library

Findings

Background

Nanopore is a powerful technology for high throughput DNA sequencing, currently commercialised by Oxford Nanopore Technologies [1]. It provides sequence base calls reconstructed from conductivity records during the translocation of a single DNA molecule through a protein pore. This approach offers portability and real time sequencing, using simple experimental protocols, for a relatively low cost. A minION device can read DNA strands of various lengths, from PCR products up to megabase genomic fragments, and current versions return at least 5×10^9 bases in one run. Therefore, it is an attractive device for sequencing libraries of amplicons that are too long for other next generation sequencing technologies. There is an increasing interest in using next generation sequencing technologies for analysing gene libraries that are highly diverse but have low variability, i.e. containing many different sequences differing from each other by only a few point mutations and for which a reference is available. This is the case in directed evolution experiments the genetic libraries typically originate from a single ancestral sequence (the wild type) that has been submitted to limited randomization, for example using error-prone PCR (epPCR) [2]. Another application is the detection of structural variants in cancer cells [3].

Unfortunately, nanopore's relatively high error rate (≈ 6-15%) prevents the accurate detection of point genetic variation directly from individual reads [4][5][6] and specific tools are not yet available. Previous work aiming at high quality sequencing from nanopore data has concentrated on polishing tools such as Nanopolish [7], or Racon [8] combined with Medaka [9]. These approaches start from a draft assembly and use the coverage depth to compute an averaged consensus at each position, via various computational approaches. Nanopolish reports an accuracy over 99.5% for a 29x sequencing coverage, and Medaka 98% in detection of single nucleotide polymorphisms (SNP) with a coverage of 100x. While these tools primarily apply to genome assembly, a number of experimental protocols were developed in order to apply these pipelines in the specific case of amplicon library sequencing. These strategies aim to read and associate several replicates of the same molecule. This has been achieved by creating sequence concatenates using rolling circular amplification [10], which retrieved an accuracy of 99.5% for coverage of 150x, and via gene barcoding prior to amplification [11] with a reported accuracy over 99.9% for 25x coverage. Inconveniently, these methods reduce the number of

different variants that can be studied, because a part of the sequencing throughput is invested in reading each sequence multiple times.

In this work, we introduce SINGLe (SNPs In Nanopore reads of Gene Libraries), a method which improves detection of single mutations via consensus calling in reads of libraries for which a reference sequence is known. SINGLe is first trained on a set of reads of the reference by a nanopore sequencer, and then it is applied to the reads of the actual library to correct their quality scores (Qscore). Finally, these values can be used in the consensus calling of individual variants.

Here, we applied SINGLe to the gene of KlenTaq, a truncated variant of the well-known Taq polymerase, of approximately 1.7 kb in length. We first tested it on a small set of seven known mutants containing 2 to 9 point mutations, and later a larger library of approximately 1200 variants. SINGLe reduced the sequencing noise, allowing a better identification of true point mutations. Therefore, as few as 5-7 reads return a trustable consensus sequence, outperforming the state-of-the-art tools for consensus computation of nanopore sequencing, Medaka and Nanopolish. This translates into a more efficient exploitation of the sequencing throughput.

SINGLe method

We used a nanopore sequencer to read 5847 strands of the wild type KlenTaq gene (length 1662 nucleotides), for which we have a ground truth sequence obtained by Sanger sequencing (supplementary Sequence S1). In this data set, we can confidently attribute mismatches between the read sequences and the known wild type as sequencing errors, and matches as correct reads. In Figure 1A (and a normalised version in Figure S1), we plotted the distribution of correct/error nucleotides according to the Qscore returned by Oxford nanopore's basecaller, Guppy. The Qscore assigned to each nucleotide tends to be low when a wrong nucleotide is assigned, as expected. Notice that the inverse is not true: some low Qscores correspond to correctly basecalled bases, so both distributions overlap. Thus, a simple classification based on the Qscores is not possible to distinguish signal from noise. We used this same dataset to plot the counts of errors by position and nucleotide (Figures 1B and S2). The errors are not homogeneously distributed, and they are more frequent in some positions of the DNA sequence than others. Previous work has also shown that nanopore sequencing produces some systematic errors [4,12][13], even for high accuracy basecalling. These two observations inspired SINGLe

as a procedure to reduce the non-random part of the sequencing errors, using the information contained in the Qscore.

The first step in SINGLe is to fit the probability of being a correct read on these wild type reads. We counted the errors in each position and nucleotide and plotted it against the Qscore (Figure 1C). We fitted this relation by a logistic regression using a binomial model, which provided a classifier able to convert the reported Qscore to the probability that this read is indeed correct. Nevertheless, as it was computed over wild type reads which do not contain true mutations, the model was heavily biassed against mutations. This is not representative of the actual proportion of errors/correct reads present in the mismatches of a set of mutants. To adapt the classifier, an *a priori* expectation of mutations ($p_{prior-right}$) is needed, which must come from independent information. In the presented case, the variant sequences originated from an epPCR, for which we possessed an estimate of the mutation rate $m_{n\rightarrow n'}$ given by the manufacturer [14]. We computed the *a priori* probability of observing nucleotide n' as $p_{prior-right}$ $(n\rightarrow n')= m_{n\rightarrow n'} \cdot \langle m\rangle/[\Sigma_n \text{counts\_reference}(n)\ \Sigma_{n'} m_{n\rightarrow n'}]$, where <m> is the mean number of mutations expected in the library (reported by the epPCR kit manufacturer), and counts_reference(n) is how many times the nucleotide n is present in the wild type DNA strand. We also set the *a priori* expectation of an observed mismatch to be a sequencing error ($p^{p,n}_{prior-error}$) to the sequencing error rate at that position in the wild type set, independently of the Qscore. Using these values we computed for each strand, position (p) and nucleotide (n), the probability of observing a mutation with Qscore=Q as $P^{p,n}_{mutation}(Q)= [\text{counts}^{p,n}(Q) / \Sigma_{Q'} \text{counts}^{p,n}(Q')] \cdot p^{p,n}_{prior-error}$ where $\text{counts}^{p,n}$ is the number of times the nucleotide n appears in position n across all the reads. Similarly, we computed the probability of observing a wild type nucleotide with Qscore=Q as $P^{p,n}_{wildtype}(Q)=[\text{counts}^{p,wildtype}(Q)/ \Sigma_{Q'} \text{counts}^{p,wildtype}(Q')] \cdot p_{prior-right}(\text{wildtype}\rightarrow n)$. Finally, we normalised $N^{p,n}_{correct}(Q)=P^{p,n}_{wildtype}/[P^{p,n}_{wildtype}(Q)+P^{p,n}_{mutation}(Q)]$. This process shifts the logistic regression towards the higher Qscore, allowing the classifier to accept a number of observed mismatches consistent with the prior expectation (Figure 1D). The fits were done independently for each position and possible mismatched nucleotides. To include deletions in this analysis (which do not have a Qscore assigned by the basecaller), we fixed their confidence value as the minimum of the Qscore of their direct nearest neighbors in the nucleotide sequence. This decision was inspired by the observation that the Qscore is correlated between consecutive nucleotides (Figure S3). Insertions were ignored as very few are expected (<1%) and it is not possible to obtain enough reads to fit all insertion possibilities. In applications where SINGLe is used to compute a high quality

consensus, the original score of the inserted bases can be carried over. We also separated the fits for forward and reverse strands as the error rate per position is different in each case (see 'Are reverse and forward reads different in Nanopore Sequencing' in Supplementary material). All together, we obtained 13296=1662×4×2 regressions, one for each position of the gene (1662 bp), for each non-wild type nucleotide or deletion (4 possibilities in total for each position) and for the forward and reverse sense of sequencing. Please refer to the supplementary material for a brief discussion on 'How many Nanopore reads are needed to fit SINGLe?'.

The regressions were then used to re-score the mismatches in the mutant library: for each non wild type nucleotide aligned to the reference, we evaluated the Qscore reported by Guppy in the SINGLe fit obtained for that particular position and nucleotide and defined this value (p_{SINGLe}) as its probability of being correct p_{right}. For nucleotides read as wild type their Qscore are directly transformed into a p_{right} according to the Q values reported by Guppy: $p_{Guppy}=1\text{-}10^{-Qscore/10}$. Finally, to compute a consensus sequence we performed a weighted count of each nucleotide (and deletion) in each position (by summing p_{SINGLe} values instead of ones), and defined the consensus nucleotide as the one with higher value. Homopolymers regions were sorted so that the deletions are always at the 3' side on the forward strand. To compare, we also computed the variant consensus sequence using p_{Guppy} instead of p_{SINGLe} for nucleotides that do not match the wildtype, or by unweighted majority vote. In these cases, we did not sort the homopolymers region as it had a detrimental effect (see Figure S4).

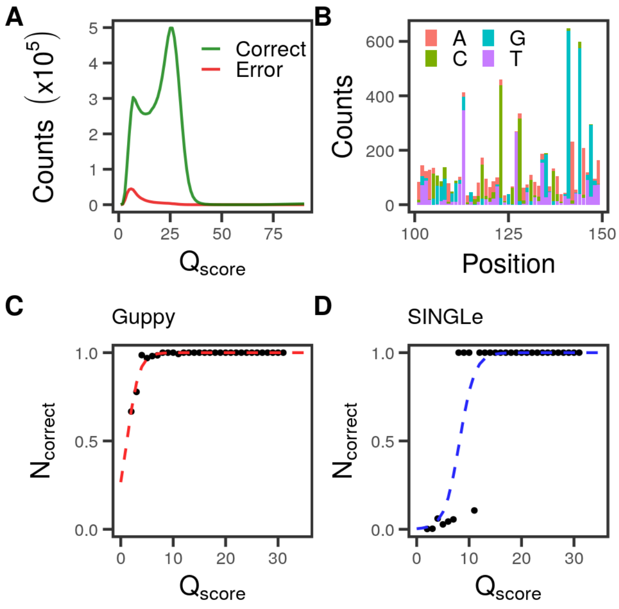

Figure 1: **A** Distribution of Qscore returned by Guppy basecaller for each nucleotide on the sequencing of the wild type KlenTaq gene, classified as correct reads (green) and errors (red). **B** Nanopore sequencing errors per position on reads of the wild type KlenTaq gene. Colours indicate the nucleotide reported. Wild type (correct) nucleotides are not plotted. Only positions 100 to 150 are shown here. An equivalent plot for all positions is available in Figure S2. **C** Example of logistic regression over reads of a known wild type sequence. Black dots are the proportion of correct nucleotides with a given Qscore in one position and in comparison to one possible error. Dashed red line is the logistic regression performed. **D** Same plots as C after data was weighted according to the prior probabilities. Blue line is p_{SINGLe}.

Analysis

We tested SINGLe in a small set composed of seven variants of KlenTaq (named #1 to #7) which we obtained from independent bacterial clones and barcoded them using the nanopore barcoding kit before sequencing. Their true sequence was obtained by Sanger. Each contains 2-9 point mutations (supplementary table S1). Variants #1 to #5 only present nucleotide substitutions. Variant #6 has 7 substitutions, two of them in consecutive positions, and one deletion in a non homopolymer region. Variant #7 has 5 substitution and a deletion in a homopolymer ('GG' to 'G-').

Signal to noise ratio

A straightforward procedure to filter errors is to only trust read nucleotides which have a probability of being correct p_{right} higher than a threshold. In this section, we compared how this process performs over the seven KlenTaq variants when using either p_{right}=p_{Guppy} or p_{right}=p_{SINGLe}.

We defined *signal* as the number of mismatches to wild type known to be actual mutations with p_{right} higher than the threshold (true positives), and *noise* as the number of those mismatches known to be a wild type nucleotide (false positives). The counts are also weighted by p_{right} for each nucleotide, i.e. instead of summing one for each occurrence, we summed the p_{right} associated with the nucleotide. Results are shown in Figure 2A (ROC curve), Figure 2B (signal-to-noise ratio) and Figure S5 (signal-to-noise ratio without weighting counts by p_{right}). For all thresholds, SINGLe has a higher signal to noise ratio (up to 6 times higher, depending on the cut off), thus facilitating the identification of actual mutations. This remains true when no cut off is applied (cut off = 0). Notice that the ratio is different for both methods at cutoff zero because the counts are weighted by p_{right}.

Consensus sequences

We compared the consensus sequences of the 7 KlenTaq variants obtained by SINGLe and by other methods. Given several aligned nanopore reads of the same variant, the consensus can be computed by simple majority counting how many times each nucleotide was read in one position and keeping the most frequent. We call this method 'no weights'. The counts can be weighted by p_{Guppy} and choose the nucleotide with larger weighted count. We call this method 'Guppy'. Or the counts can be weighted by p_{SINGLe} ('SINGLe' method). We also computed the consensus using Nanopolish, which works directly on the raw electrical signal instead of the basecalled sequences in combination with HMMs to detect base variations [7], Medaka that counts nucleotides and uses a neural network to compare to a draft assembly and define mutations [9], and NextPolish which takes into account the neighbours of the nucleotide [15].

For each method, we computed the consensus independently for each variant using subsets of 3 to 50 sequences drawn randomly from all available reads, and repeated 50 times for each subset size. In Figure 2D, we plotted the success rate on the consensus computation, i.e. how many times the obtained consensus matches exactly the true sequence. For variants #1 to #6, the convergence is faster when using SINGLe weights: perfect consensuses are obtained for more than 90% of attempts starting from 5-7 sequences. Nanopolish reaches a 90% of success rate in 8 to 15 reads but fails for variant #1 (it systematically misses mutation G23A). Medaka requires more than 20 reads to reach 90% of success, and it does not converge for variants #4 and #5. NextPolish needs between 15 and 50 reads depending on the variant. Finally, using p_{guppy} or no weights has a poorer performance, not reaching 90% of success for 50 reads for any of the variants #1-#6. Notice that variant #6 has two consecutive mutations and they are properly detected by SINGLe. Variant #7 has a deletion in a homopolymer which is a challenging mutation to detect. In this case, SINGLe needs 35 reads to converge to the true sequence, still outperforming Medaka, Guppy and no weights (they need 45 reads). Only Nanopolish converges faster, with 15 reads.

Figure 2C shows the total number of mismatches (averaged over the 50 trials) reported by each method for variant #3, according to the number of reads used to compute the consensus. For any set size, SINGLe reports the closest number of true mutations compared to the other methods. In Figure S6 this is analysed in more detail: we classified the nucleotides in each consensus sequence according to true/false mutations and true/false wild types and observed that actually SINGLe detects true mutations with the fewest number of

reads while keeping the lowest rate of false mutations. Nanopolish has a high rate of false wild types and underestimates the true mutations. Medaka has a similar behaviour and on top adds false mutations. Consensus using Guppy scores or no weight report a high number of false mutations, and so does NextPolish (though with a lower error rate).

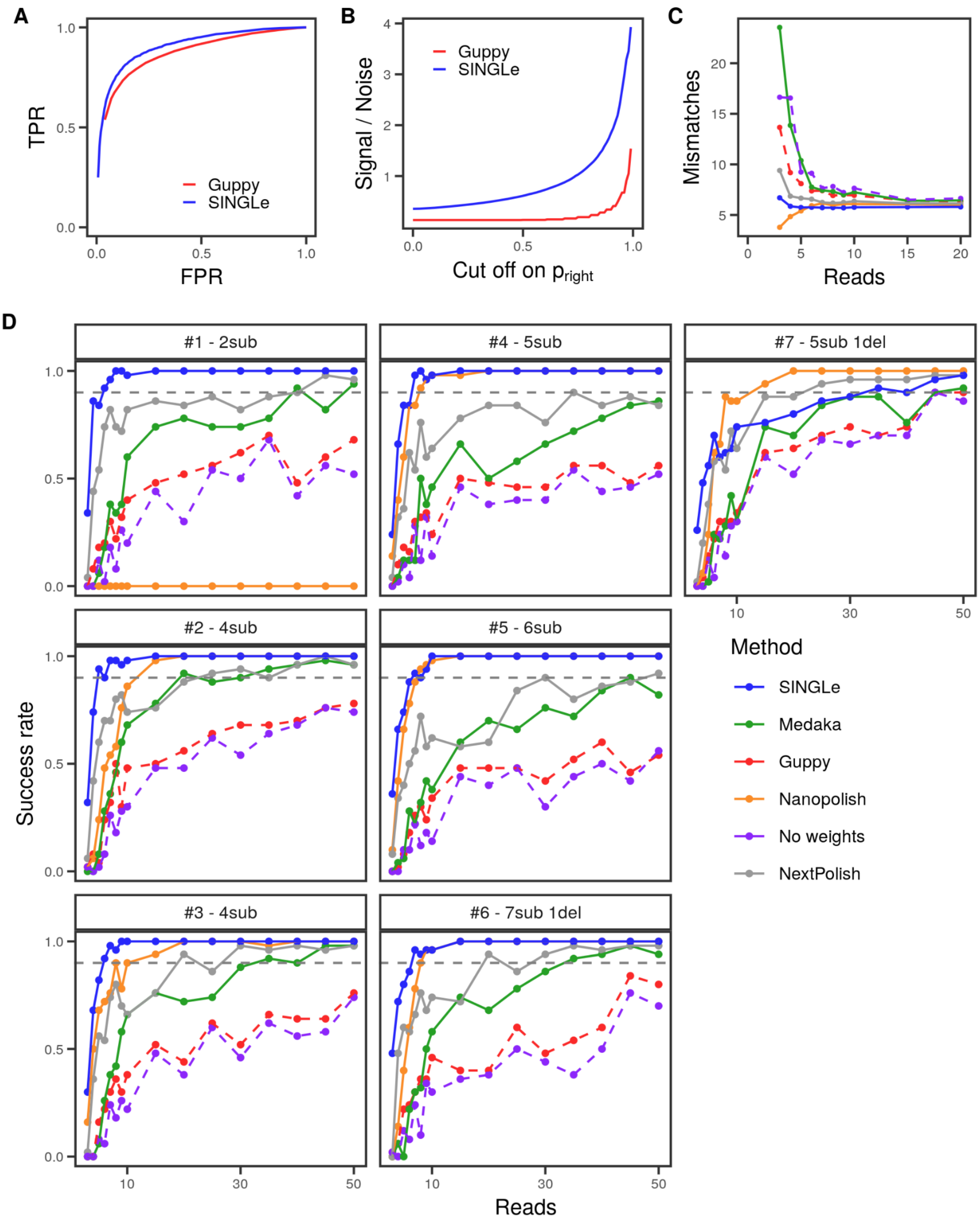

Figure 2: A True positive rate (TPR) vs false positive rate (FPR) (or ROC curve) for SINGLe (blue) and Guppy (red). **B** Signal to noise ratio when classifying mismatches as mutations when they reach p_{right} indicated on the x axis, for SINGLe (blue) and Guppy (red). **C** Mean number of mismatches between the consensus of variant #3 (4 sub) and the wild type sequence when using a subset of sequences (x axis) to compute the consensus. Colours indicate the method used for computing the consensus. **D** Success rate on obtaining the true sequence when computing the consensus with a subset of reads. The size of the subset used is indicated on the x axis. The success rate is computed over 50 different consensus on independent subsets of reads. The colours indicate the method used for the consensus calling. Each panel has one of the 7 variants analysed, and the number of substitutions (sub) and deletions (del) are indicated on top.

Consensus in a large gene library

We also tested SINGLe on a large library of mutants of the Klen Taq gene obtained by epPCR, containing approximately 1200 variants with a mean mutation rate of 8muts/kB. The variants are unknown, but associated with a barcode of 36 nucleotides downstream the STOP codon. We sequenced the library using nanopore, grouped the reads according to the barcode and computed the consensus sequence for the reads associated to each barcode. We first confirmed the consistency of the different consensus methods on this library, with respect to the results obtained in previous sections of this article. We chose the most represented barcode (901 reads) and computed the success rate using Medaka and SINGLe for subsets of reads. As ground truth we used the consensus computed with all available reads, which is the same for all methods except Nanopolish (Table S2). As shown in Figure 3A, SINGLe returns the correct consensus sequence over 90% of the times when using at least 5 reads, while Medaka needs 15. Similar results were obtained for the other 9 most frequent barcodes using various methods for computing the consensus sequence (Table S2 and Figure S7).

We computed consensus sequences using SINGLe or Medaka for all the mutants in our library, provided that the identifying barcode is present at least four times in our dataset, and compared the number of mutations reported by both methods (Figure 3B). Out of the 1174 variants for which we had at least 10 reads, both methods report the same consensus in 1039 cases (89%). Among the 610 variants for which we had 6 to 10 reads, we obtained the same consensus sequence using Medaka or SINGLe only in 339 variants (56%), and for the 839 variants with less than 5 reads only 23% of the variants obtained the same consensus with both

methods. For the other variants, independently of the number of available reads, Medaka returns more mutations than SINGLe in the wide majority of cases. This is consistent with the observation in the previous section: Medaka tends to predict more mutations than there actually are. Less than 1% of all computed consensuses have more mutations by SINGLe than by Medaka.

If SINGLe improves the consensus sequences by reducing systematic errors from the basecalling, then the mutations predicted for a randomly mutated gene library should be homogeneously distributed along the sequence. In Figure 3C we show that this is the case. When there are more than 10 reads available in each cluster, SINGLe and Medaka predict mutations that are similarly distributed throughout the gene, as reflected by the skewness of the distribution of mutation counts (sk), around 0.8 in both cases. When there are only 6 to 10 reads available, Medaka tends to predict mutations on some preferred spots, increasing sk to 3.8. The effect is even larger for 5 or less reads: Medaka shows strong systematic errors (sk around 7), while SINGLe's reaches sk=1.3. In Figure S8, this same analysis is performed for other consensus calling methods and they all have a higher sk than SINGLe. Finally, we also compared the bias of the mutations in our library to the one reported by the manufacturer (Figure S9). All methods (except Nanopolish) had a correlation of .94 when sequences with more than 10 reads are used. Only SINGLe reaches this value for the sequences with 4 or 5 reads available.

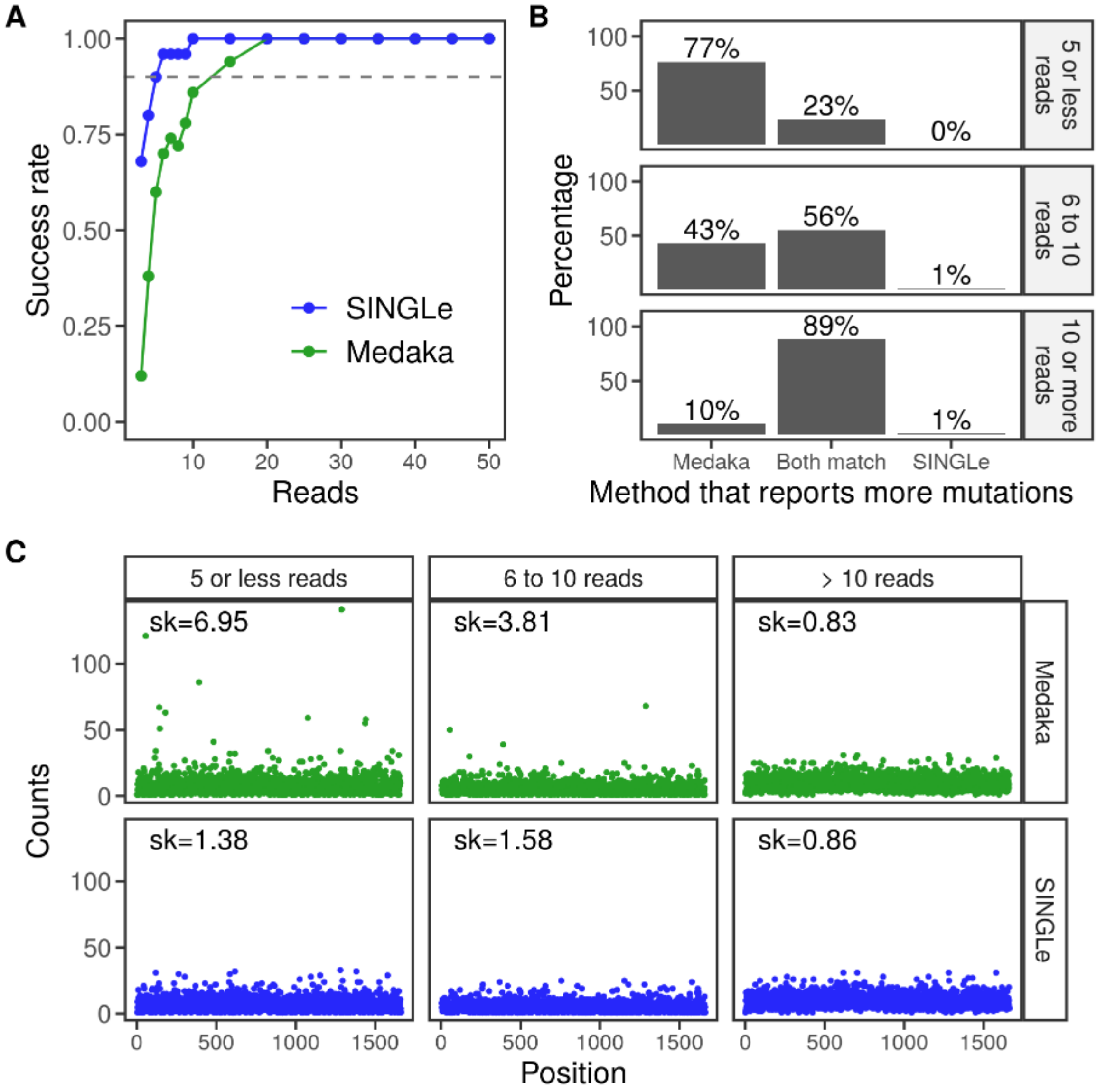

Figure 3: A For the most frequent variant in the library, success rate of the consensus computed from subsets of available reads using Medaka (green) or SINGLe (blue). **B** Comparison of the consensus sequences returned by Medaka and SINGLe. We splitted the results by the number of reads available for each variant (less than 5, 6 to 10, and more than 10). The bars on the middle indicate the proportion of variants whose consensus sequence is the same either computed by Medaka or by SINGLe. The left bar indicates the proportion of variants whose consensus sequences have more mutations when computed with Medaka than when computed by SINGLe, and the right bar the opposite. **C** Mismatches to wild type on the consensus computed by Medaka (upper panels) and SINGLe (lower panels) by position, and classified according to the number of reads

available for the consensus computation (label on top). To quantify the systematicity of the mismatches, we used the skewness (sk) of the distribution of counts.

Discussion

The relatively high error rate in single molecule nanopore sequencing limits some applications such as the analysis of libraries containing many different, but genetically similar, sequences. The approach we introduce here, SINGLe, leverages the fact that the sequencing errors in this case are partly systematic, as previously noted [4,12]. We accumulate many reads from the reference gene to build a sequence-specific error model that locally corrects for the sequencing biases. Applying this procedure on a set of seven variants of the KlenTaq gene with an average mutation rate of 3 bases/kb, we showed that correcting the confidence values provides a large increase in the signal to noise ratio. Consequently, the consensus calling returns 90% of perfect results from typically 5-7 reads. This implies a faster convergence than other methods currently used: nanopolish requires 8 to 15 reads, Medaka needs at least 20-50 reads and NextPolish between 15 and 50 reads to achieve a similar performance. Therefore, with SINGLe a lower burden on the sequencing throughput is taken to obtain true consensus sequences. Even when using very few reads to compute a consensus, SINGLe detects the true mutations (high sensibility) without increasing the false mutations as much as other methods do (specificity). We also tested SINGLe in a library of approximately 1200 variants of KlenTaq, which are uniquely barcoded for clustering of the DNA strands. When many reads are available, Medaka and SINGLe report a similar number of mutations. But for variants with few reads, only 23% of the times both methods match and Medaka predicts more mutations in the remaining 77% of the cases. Furthermore, when we plotted the location of the mutations detected by each method, we observed that the ones returned by SINGLe are spread along the strand, while other methods present some hot spot positions. We interpret them as a consequence of the systematic errors produced by nanopore sequencing and that SINGLe overcomes.

SINGLe needs an expected average number of mutations in the test set. Here, our reference sequence was assumed to be perfect, and we could evaluate precisely the average mutation rate in the test set, because it originated from a controlled experimental mutagenesis protocol. In other situations it would be possible to use short read sequencing, for example Illumina, to evaluate this number. If the full sequence is submitted to short read high quality sequencing, it would even be possible to obtain more precise priors, for example specific to

each position and nucleotide. Our approach would then be used to phase these statistical mutations to single long reads. An underlying assumption of our method is that the distribution of Qscore observed at a particular position for the wild type base reflects appropriately the distribution of Qscore that would be observed for a variant base at that position. This approximation is necessary since the error model is built from a single sequence and hence has a single ‘‘true” base per position. Fortunately, the difference in Qscore distributions for ‘‘true” versus ‘‘error” seems large enough for our method to perform well within that approximation.

SINGLe needs to characterise the sequencing errors done on an appropriate reference sequence. Because it applies a probabilistic approach on the erroneous nanopore reads, irrespective of what mechanism actually causes the systematic errors, it should not depend on the genes’ properties. On the other hand, SINGLe is limited to analyse variants which are close neighbours of the reference, and where mutations can be considered to be independent. We did not try to adapt the method to detect alterations beyond point replacements or deletions, which may require a more complex analysis pipeline. Encouragingly, variant 6 contained two contiguous mutations, and was properly analysed by our consensus approach. Finally, to our knowledge SINGLe is the first tool fully focused in analysing gene libraries sequenced by standard nanopore technology. Our approach provides a large improvement of signal to noise at very little experimental effort or throughput reduction. The reads of the reference sequences, needed to train SINGLe, can be obtained simultaneously with the libraries using standard barcoding protocols and only use a small fraction of the sequencing throughput. There are no other modifications to the experimental protocol, and the computational error correction process can be simply added to any analysis pipeline after basecalling. SINGLe allows an exploitation of the sequencing throughput 10 to 20 times better than Medaka, and 4 times better than Nanopolish but with a better performance as we found that Nanopolish systematically misses some mutations. Therefore, SINGLe can be combined with experimental methods to obtain more accurate consensus sequences of large libraries of long genetic elements.

SINGLe is available as an R package that fits the errors on reads of a reference sequence, it then assigns p_{SINGLe} values on a library and a consensus sequence can be computed if a table with the barcodes in each read is provided. Its inputs are the .sam files obtained after a minimap2 alignment and samtool nucleotides count, the prior mutational rate and the reference sequence.

Methods

Samples preparation and sequencing

KlenTaq wildtype and seven variants: the wild type gene was amplified using a high fidelity PCR (Q5 polymerase from NEB) from a stored plasmid following NEB recommendations with primers GGGATTATTCTTTGGCGCTCAGCCAAT and ACCATGCGTCTGCTGCATGAAT. The mutants were obtained via epPCR using Agilent's kit GeneMorph II. We started from 1.1 nM of dam-methylated DNA, and we used the same primers as for the wild type. Thermocycling was performed as follows: 95°C for 2 min, followed by 25 cycles of [95°C for 30 sec + 65°C for 30 sec + 72°C for 2 min] and a final extension at 72°C for 10 min. In both cases, we digested the PCR product with DpnI (NEB) and purified it using columns (Macherey-Nagel). We put the genes in a pIVEX vector via Gibson assembly (NEB Hi-Fi DNA assembly) using 125ng of gene DNA (93ng for wild type gene), 100 ng of vector in an approximately 2:1 insert:vector molar ratio and incubated for 15 min at 50°C. We purified and concentrated DNA with a Zymo Research kit. We transformed the product into chemocompetent KRX bacteria. We spread them on a Petri dish with LB and Ampicillin (one plate for the library, another for the wild type). We incubated overnight at 37°C and the next day we randomly picked some clones from the library and from the wild type. We verified the presence of the plasmid via colony PCR using DreamTaq polymerase (Thermofisher). Positive clones were grown overnight in liquid LB with antibiotics and mini-prepped to obtain plasmid DNA. A fraction of the plasmid was used for high quality sequencing (Sanger sequencing). Another fraction of each clone was used for amplification by PCR with Q5 polymerase (NEB) in independent tubes. We used primers which included the minION barcodes adapters: ACTTGCCTGTCGCTCTATCTTCAGTGTGCTGGAATTCGCCCTTTTA and TTTCTGTTGGTGCTGATATTGCAGACCACAACGGTTTCCCTCTAGAAATA. Thermocycling was performed as follows: 98°C for 30sec, 23 cycles of [98°C for 10sec + 59°C for 30sec + 72°C for 1min], final extension at 72°C for 2min. We digested the product with Dpn1 (NEB), gel purified it using Macherey-Nagel kit. We proceeded to sequencing following standard Oxford Nanopore protocols. We used one barcode for wild type and one for each of the mutants 1 to 7. We used kits EXP-PCB001 for barcoding, SQK-LSK108 for ligation, EXP-LLB001 for flow cell loading. minION flow cell version was R9.4/FLO-MIN106, thus the sequencing was 1D. After data pre-processing (see section minION reads pre-processing), we had the following reads per variant: 716 (variant 1), 453 (variant 2), 645 (variant 3), 428 (variant 4), 422 (variant 5), 369 (variant 6), 358 (variant 7).

KlenTaq large library: Mutants were obtained via epPCR using Agilent's kit GeneMorph II. We started from 0.12ng of DNA (pIVEX vector containing KlenTaq gene). We used primers GCCAGTGTGCTGGAATTCGCCCTTTTATTAATG and CCCTCTAGAAATAATTTTGTTTAACTTTAAGAAGGAGATATACCATG. Thermocycling was performed as follows: 95°C for 2 min, followed by 30 cycles of [95°C for 30 sec + 58°C for 30 sec + 72°C for 2 min] and final extension at 72°C for 10 min. We

digested the product with DpnI (NEB) for 1 hour and purified it using columns (Macherey-Nagel). We put the mutagenized genes in a pIVEX vector, previously amplified with primers that incorporated a random barcode downstream the STOP codon (aattccagcacactggcDHVBDHVBDHVBDHVBDHVBDHVBDHVBDHVBDHVBaagcccgaaaggaagctgag and ttaaagttaaacaaaattatttctagagggaaaccgttg). Previously a SalI site was added into the pIVEX vector, upstream the T7 promoter. We used NEB Hi-Fi DNA assembly using 100ng of vector DNA, 103 ng of insert DNA, in a 2:1 insert:vector molar ratio and incubated for 15 min at 50°C. We purified and concentrated DNA with a Zymo Research kit. We transformed the product into chemocompetent T7 Express lysY/Iq bacteria (NEB C3013I) via heat shock and spread them on a Petri dish with LB and Ampicillin. We incubated overnight at 37°C and obtained around 1000 CFU. We grew the library overnight at 37°C in liquid LB with Ampicillin and extracted the plasmid using Macheray Nagel kit. We digested the library plasmid with EcoRI-HF and SAlI-HF from NEB, in rCutSmart buffer at 37°C for 1h40min plus an inactivation step at 65°C for 20'. We added proteinase K and incubated at 37°C for 15 min. We gel purified the sample to keep the fragment containing the KlenTaq gene, and used Zymo research kit for concentration and purification of the DNA. We dialysed our sample using millipore membranes for 1h. For nanopore oxford sequencing we used a flongle (version FLO-FLG001), and SQK-LSK110 kit for sample preparation. We started with 110 fmol and loaded 50 fmol into the flow cell.

minION reads pre-processing

minION raw data was basecalled using ONT Guppy version 5.1 using model dna_r9.4.1_450bps_sup.cfg. We kept reads which had a length of 1700 to 2100 nucleotides and a mean Qscore larger than 10 (except for the large library, for which we used a Qscore cut-off of 15). For the wild type and the seven variants we also used Guppy for demultiplexing. For the KlenTaq large library, we used a custom script made in the lab to group barcodes by exact match. Sequences were aligned to the reference wild type using minimap2 version 2.21 [16], using the options minimap2 -ax map-ont --sam-hit-only. We used samtools 1.7 [17] to create sorted bam files via the commands samtools view -S -b and samtools sort.

Consensus by Nanopolish

We used the scripts provided by Oxford Nanopore, multi_to_single_fast5 and single_to_multi_fast5 (version 4.0) to split fast5 files and reassemble them according to the associated barcode. We then used the commands nanopolish index ; samtools sort; samtools index; and nanopolish variants --consensus, according to the Nanopolish manual, to compute the consensus. Nanopolish version is 0.13.3.

Consensus by Medaka

We used racon (version 1.4) to polish our sequences and used it as an input in the consensus computation by medaka (version 1.4) which we run by the command line medaka_consensus with default parameters.

Consensus by NextPolish

We used NextPolish (version 1.4.1) with options task=best, rerun=3, genome= KlenTaq wild type sequence, and lgs_options -min_read_len 1k -max_depth 100.

Availability

Project name: SINGLe

Project home page: https://github.com/rocioespci/single, also available in bioconductor 10.18129/B9.bioc.single

Operating system: Platform independent

Programming language: R (>= 4.1).

Other requirements: Imports R packages Biostrings, BiocGenerics, dplyr, GenomicAlignments, IRanges, methods, reshape2, rlang, Rsamtools, stats, stringr, tidyr, utils. To create input files, minimap2 and samtools are required (or equivalent software to align data to reference and create a bam file).

Licence: MIT

Data availability

The data set supporting the results of this article are available in the European Nucleotide Archive repository, ERP135743, run ERR8778685 (nanopore reads of seven mutants and wild type) and ERR8778797 (nanopore reads of library).

Funding

European Research Council, H2020 Marie Sklodowska-Curie Research and Innovation program, 845976, R Espada; European Research Council, Consolidator Grant No. 647275 ProFF, Y. Rondelez;

Competing interests

The authors declare that they have no competing interests.

List of abbreviations

epPCR: error-prone PCR

PCR: polymerase chain reaction

SINGLe: SNPs In Nanopore reads of Gene Libraries

SNP: single nucleotide polymorphisms

Qscore: Quality score

Sk: Skewness

Authors' contributions

RE and YR designed research and conceptualization. YR performed supervision of the research. RE, and ADM performed experimental research. RE, NK and YR perform methodology and formal analysis. RE wrote the software. RE and YR wrote the manuscript, ADM and NK review and edited the manuscript.

References

1. Oxford Nanopore Technologies. [cited 1 Feb 2022]. Available: https://nanoporetech.com/
2. Sze MA, Schloss PD. The Impact of DNA Polymerase and Number of Rounds of Amplification in PCR on 16S rRNA Gene Sequence Data. mSphere. 2019;4. doi:10.1128/mSphere.00163-19
3. Thibodeau ML, O'Neill K, Dixon K, Reisle C, Mungall KL, Krzywinski M, et al. Improved structural variant interpretation for hereditary cancer susceptibility using long-read sequencing. Genet Med. 2020;22: 1892–1897.
4. Wang Y, Zhao Y, Bollas A, Wang Y, Au KF. Nanopore sequencing technology, bioinformatics and applications. Nature Biotechnology. 2021. pp. 1348–1365. doi:10.1038/s41587-021-01108-x
5. Sedlazeck FJ, Rescheneder P, Smolka M, Fang H, Nattestad M, von Haeseler A, et al. Accurate detection of complex structural variations using single-molecule sequencing. Nat Methods. 2018;15: 461–468.
6. Gong L, Wong C-H, Cheng W-C, Tjong H, Menghi F, Ngan CY, et al. Picky comprehensively detects high-resolution structural variants in nanopore long reads. Nat Methods. 2018;15: 455–460.
7. Loman NJ, Quick J, Simpson JT. A complete bacterial genome assembled de novo using only nanopore sequencing data. Nat Methods. 2015;12: 733–735.
8. Vaser R, Sović I, Nagarajan N, Šikić M. Fast and accurate de novo genome assembly from long uncorrected reads. Genome Res. 2017;27: 737–746.
9. Website. Available: https://nanoporetech.github.io/medaka
10. Chenhao Li, Kern Rei Chng, Esther Jia Hui Boey, Amanda Hui Qi Ng, Andreas Wilm, and Niranjan Nagarajan. Incseq: accurate single molecule reads using nanopore sequencing. Gigascience. 2016;5: s13742–016.
11. Karst SM, Ziels RM, Kirkegaard RH, Sørensen EA, McDonald D, Zhu Q, et al. High-accuracy long-read amplicon sequences using unique molecular identifiers with Nanopore or PacBio sequencing. Nat Methods. 2021;18: 165–169.

12. Krishnakumar R, Sinha A, Bird SW, Jayamohan H, Edwards HS, Schoeniger JS, et al. Systematic and stochastic influences on the performance of the MinION nanopore sequencer across a range of nucleotide bias. Sci Rep. 2018;8: 3159.

13. Huang Y-T, Liu P-Y, Shih P-W. Homopolish: a method for the removal of systematic errors in nanopore sequencing by homologous polishing. Genome Biol. 2021;22: 95.

14. Manual available in https://www.agilent.com/cs/library/usermanuals/Public/200550.pdf

15. Hu J, Fan J, Sun Z, Liu S. NextPolish: a fast and efficient genome polishing tool for long-read assembly. Bioinformatics. 2020;36: 2253–2255.

16. Li H. Minimap2: pairwise alignment for nucleotide sequences. Bioinformatics. 2018;34: 3094–3100.

17. Li H, Handsaker B, Wysoker A, Fennell T, Ruan J, Homer N, et al. The Sequence Alignment/Map format and SAMtools. Bioinformatics. 2009;25: 2078–2079.

Click here to access/download
Supplementary Material
SINGLe_Supplementary_reviewed.pdf
