# Supplementary material for: Accurate gene consensus at low nanopore coverage
Source: Gigascience. 2022 Nov 9;11:giac102. doi: 10.1093/gigascience/giac102 (PMC9646519; doi:10.1093/gigascience/giac102)
Supplement: giac102_GIGA-D-22-00024_Revision_2 [file giac102_giga-d-22-00024_revision_2.pdf]

GigaScience
Accurate gene consensus at low nanopore coverage
--Manuscript Draft--

**Manuscript Number:**	GIGA-D-22-00024R2
**Full Title:**	Accurate gene consensus at low nanopore coverage
**Article Type:**	Technical Note
**Funding Information:**	H2020 European Research Council (845976) — PhD Rocio Espada<br>H2020 European Research Council (647275) — PhD Rondelez Yannick
**Abstract:**	Background Nanopore technologies allow high throughput sequencing of long strands of DNA at the cost of a relatively large error rate. This limits its use in the reading of amplicon libraries in which there are only a few mutations per variant and therefore they are easily confused with the sequencing noise. Consensus calling strategies reduce the error but sacrifice part of the throughput on reading typically 30 to 100 times each member of the library.<br>Findings In this work, we introduce SINGLe (SNPs In Nanopore reads of Gene Libraries), an error correction method to reduce the noise in nanopore reads of amplicons containing point variations. SINGLe exploits that in an amplicon library, all reads are very similar to a wild type sequence from which it is possible to experimentally characterise the position-specific systematic sequencing error pattern. Then, it uses this information to reweight the confidence given to nucleotides that do not match the wild type in individual variant reads, and incorporates it on the consensus calculation.<br>Conclusions We tested SINGLe in a mutagenic library of the KlenTaq polymerase gene, where the true mutation rate was below the sequencing noise. We observed that contrary to other methods, SINGLe compensates for the systematic errors made by the basecallers. Consequently, SINGLe converges to the true sequence using as little as 5 reads per variant, fewer than the other available methods.
**Corresponding Author:**	Rondelez Yannick, PhD<br>ESPCI Paris/CNRS/PSL<br>Paris, FRANCE
**Corresponding Author Secondary Information:**	
**Corresponding Author's Institution:**	ESPCI Paris/CNRS/PSL
**Corresponding Author's Secondary Institution:**	
**First Author:**	Rocio Espada
**First Author Secondary Information:**	
**Order of Authors:**	Rocio Espada<br>Adèle Dramé-Maigné<br>Nikola Zarevski<br>Rondelez Yannick, PhD
**Order of Authors Secondary Information:**	
**Response to Reviewers:**	Dear Dr Zauner,<br><br>We submit the manuscript with the requested modifications:<br><br>1. Our dataset in GigaDB has been added to the bibliography and referred to in the Data availability section:<br>"The data set supporting the results of this article are available in the European Nucleotide Archive repository, ERP135743, run ERR8778685 (nanopore reads of

	seven mutants and wild type) and ERR8778797 (nanopore reads of library), and in GigaDB [18] (Sanger sequencing for the seven mutants of Klen Taq, sequence of wild type KlenTaq gene, and the full code and processed data to reproduce the figures in the manuscript and supplementary material)."<br><br>2 and 3. SINGLe has been registered in Scicrunch and bio.tools and cited in the availability section:<br>"Software is registered in scicrunch.org (identifier SINGLe, RRID:SCR_022810) and biotools (https://bio.tools/SINGLe)."<br>Please notice that in scicrunch the curation takes around 5 business days so the link should be available by the end of this week or beginning of next one.<br><br>4. We have checked that our manuscript follows the journal style.<br><br>Sincerely,
**Additional Information:**	
**Question**	**Response**
Are you submitting this manuscript to a special series or article collection?	No
**Experimental design and statistics**<br><br>Full details of the experimental design and statistical methods used should be given in the Methods section, as detailed in our Minimum Standards Reporting Checklist. Information essential to interpreting the data presented should be made available in the figure legends.<br><br>Have you included all the information requested in your manuscript?	Yes
**Resources**<br><br>A description of all resources used, including antibodies, cell lines, animals and software tools, with enough information to allow them to be uniquely identified, should be included in the Methods section. Authors are strongly encouraged to cite Research Resource Identifiers (RRIDs) for antibodies, model organisms and tools, where possible.<br><br>Have you included the information requested as detailed in our Minimum Standards Reporting Checklist?	Yes

Powered by Editorial Manager® and ProduXion Manager® from Aries Systems Corporation

**Availability of data and materials**<br><br>All datasets and code on which the conclusions of the paper rely must be either included in your submission or deposited in publicly available repositories (where available and ethically appropriate), referencing such data using a unique identifier in the references and in the "Availability of Data and Materials" section of your manuscript.<br><br>Have you have met the above requirement as detailed in our Minimum Standards Reporting Checklist?	Yes

Powered by Editorial Manager® and ProduXion Manager® from Aries Systems Corporation

Manuscript

Click here to access/download;Manuscript;SINGLE_manuscript.docx

Title page

Title:

Accurate gene consensus at low nanopore coverage

Authors:

Espada Rocío.

Gulliver Lab, ESPCI Paris, PSL University, CNRS, 75005 Paris, France.

Contact: rocio.espada@espci.fr

ORCID 0000-0003-3829-473X

Zarevski Nikola. Gulliver,

Gulliver Lab, ESPCI Paris, PSL University, CNRS, 75005 Paris, France.

Contact: niko.zarevski@gmail.com

ORCID 0000-0001-5045-2425

Dramé-Maigné Adèle.

Gulliver Lab, ESPCI Paris, PSL University, CNRS, 75005 Paris, France.

Contact: docdeldou@gmail.com

ORCID 0000-0003-3586-0361

Rondelez Yannick.

Gulliver Lab, ESPCI Paris, PSL University, CNRS, 75005 Paris, France.

Contact: yannick.rondelez@espci.fr

ORCID 0000-0002-2565-476X

Corresponding author

Abstract

Background Nanopore technologies allow high throughput sequencing of long strands of DNA at the cost of a relatively large error rate. This limits its use in the reading of amplicon libraries in which there are only a few mutations per variant and therefore they are easily confused with the sequencing noise. Consensus calling strategies reduce the error but sacrifice part of the throughput on reading typically 30 to 100 times each member of the library.

Findings In this work, we introduce SINGLe (SNPs In Nanopore reads of Gene Libraries), an error correction method to reduce the noise in nanopore reads of amplicons containing point variations. SINGLe exploits that in an amplicon library, all reads are very similar to a wild type sequence from which it is possible to experimentally characterise the position-specific systematic sequencing error pattern. Then, it uses this information to reweight the confidence given to nucleotides that do not match the wild type in individual variant reads, and incorporates it on the consensus calculation.

Conclusions We tested SINGLe in a mutagenic library of the KlenTaq polymerase gene, where the true mutation rate was below the sequencing noise. We observed that contrary to other methods, SINGLe compensates for the systematic errors made by the basecallers. Consequently, SINGLe converges to the true sequence using as little as 5 reads per variant, fewer than the other available methods.

Keywords:

Nanopore sequencing; consensus sequence; low coverage; gene library

Findings

Background

Nanopore is a powerful technology for high throughput DNA sequencing, currently commercialised by Oxford Nanopore Technologies [1]. It provides sequence base calls reconstructed from conductivity records during the translocation of a single DNA molecule through a protein pore. This approach offers portability and real time

sequencing, using simple experimental protocols, for a relatively low cost. A minION device can read DNA strands of various lengths, from PCR products up to megabase genomic fragments, and current versions return at least 5×10^9 bases in one run. Therefore, it is an attractive device for sequencing libraries of amplicons that are too long for other next generation sequencing technologies. There is an increasing interest in using next generation sequencing technologies for analysing gene libraries that are highly diverse but have low variability, i.e. containing many different sequences differing from each other by only a few point mutations and for which a reference is available. This is the case in directed evolution experiments the genetic libraries typically originate from a single ancestral sequence (the wild type) that has been submitted to limited randomization, for example using error-prone PCR (epPCR) [2]. Another application is the detection of structural variants in cancer cells [3].

Unfortunately, nanopore's relatively high error rate (≈ 6-15%) prevents the accurate detection of point genetic variation directly from individual reads [4][5][6] and specific tools are not yet available. Previous work aiming at high quality sequencing from nanopore data has concentrated on polishing tools such as Nanopolish [7], or Racon [8] combined with Medaka [9]. These approaches start from a draft assembly and use the coverage depth to compute an averaged consensus at each position, via various computational approaches. Nanopolish reports an accuracy over 99.5% for a 29x sequencing coverage, and Medaka 98% in detection of single nucleotide polymorphisms (SNP) with a coverage of 100x. While these tools primarily apply to genome assembly, a number of experimental protocols were developed in order to apply these pipelines in the specific case of amplicon library sequencing. These strategies aim to read and associate several replicates of the same molecule. This has been achieved by creating sequence concatenates using rolling circular amplification [10], which retrieved an accuracy of 99.5% for coverage of 150x, and via gene barcoding prior to amplification [11] with a reported accuracy over 99.9% for 25x coverage. Inconveniently, these methods reduce the number of different variants that can be studied, because a part of the sequencing throughput is invested in reading each sequence multiple times.

In this work, we introduce SINGLe (SNPs In Nanopore reads of Gene Libraries), a method which improves detection of single mutations via consensus calling in reads of libraries for which a reference sequence is known. SINGLe is first trained on a set of reads of the reference by a nanopore sequencer, and then it is applied to the reads of the actual library to correct their quality scores (Qscore). Finally, these values can be

used in the consensus calling of individual variants.

Here, we applied SINGLe to the gene of KlenTaq, a truncated variant of the well-known Taq polymerase, of approximately 1.7 kb in length. We first tested it on a small set of seven known mutants containing 2 to 9 point mutations, and later a larger library of approximately 1200 variants. SINGLe reduced the sequencing noise, allowing a better identification of true point mutations. Therefore, as few as 5-7 reads return a trustable consensus sequence, outperforming the state-of-the-art tools for consensus computation of nanopore sequencing, Medaka and Nanopolish. This translates into a more efficient exploitation of the sequencing throughput.

SINGLe method

We used a nanopore sequencer to read 5847 strands of the wild type KlenTaq gene (length 1662 nucleotides), for which we have a ground truth sequence obtained by Sanger sequencing (supplementary Sequence S1). In this data set, we can confidently attribute mismatches between the read sequences and the known wild type as sequencing errors, and matches as correct reads. In Figure 1A (and a normalised version in Figure S1), we plotted the distribution of correct/error nucleotides according to the Qscore returned by Oxford nanopore's basecaller, Guppy. The Qscore assigned to each nucleotide tends to be low when a wrong nucleotide is assigned, as expected. Notice that the inverse is not true: some low Qscores correspond to correctly basecalled bases, so both distributions overlap. Thus, a simple classification based on the Qscores is not possible to distinguish signal from noise. We used this same dataset to plot the counts of errors by position and nucleotide (Figures 1B and S2). The errors are not homogeneously distributed, and they are more frequent in some positions of the DNA sequence than others. Previous work has also shown that nanopore sequencing produces some systematic errors [4,12,13], even for high accuracy basecalling. These two observations inspired SINGLe as a procedure to reduce the non-random part of the sequencing errors, using the information contained in the Qscore.

The first step in SINGLe is to fit the probability of being a correct read on these wild type reads. We counted the errors in each position and nucleotide and plotted it against the Qscore (Figure 1C). We fitted this relation by a logistic regression using a binomial model, which provided a classifier able to convert the reported Qscore to the probability that this read is indeed correct. Nevertheless, as it was computed over wild type reads which

do not contain true mutations, the model was heavily biassed against mutations. This is not representative of the actual proportion of errors/correct reads present in the mismatches of a set of mutants. To adapt the classifier, an *a priori* expectation of mutations ($p_{prior-right}$) is needed, which must come from independent information. In the presented case, the variant sequences originated from an epPCR, for which we possessed an estimate of the mutation rate $m_{n\rightarrow n'}$ given by the manufacturer [14]. We computed the *a priori* probability of observing nucleotide n' as $p_{prior-right}(n\rightarrow n') = m_{n\rightarrow n'} \cdot \langle m\rangle / [\Sigma_n \text{counts\_reference}(n)\ \Sigma_{n'} m_{n\rightarrow n'}]$, where <m> is the mean number of mutations expected in the library (reported by the epPCR kit manufacturer), and counts_reference(n) is how many times the nucleotide n is present in the wild type DNA strand. We also set the *a priori* expectation of an observed mismatch to be a sequencing error ($p^{p,n}_{prior-error}$) to the sequencing error rate at that position in the wild type set, independently of the Qscore. Using these values we computed for each strand, position (p) and nucleotide (n), the probability of observing a mutation with Qscore=Q as $P^{p,n}_{mutation}(Q) = [\text{counts}^{p,n}(Q) / \Sigma_{Q'} \text{counts}^{p,n}(Q')] \cdot p^{p,n}_{prior-error}$ where $\text{counts}^{p,n}$ is the number of times the nucleotide n appears in position n across all the reads. Similarly, we computed the probability of observing a wild type nucleotide with Qscore=Q as $P^{p,n}_{wildtype}(Q) = [\text{counts}^{p,wildtype}(Q) / \Sigma_{Q'} \text{counts}^{p,wildtype}(Q')] \cdot p_{prior-right}(\text{wildtype}\rightarrow n)$. Finally, we normalised $N^{p,n}_{correct}(Q) = P^{p,n}_{wildtype} / [P^{p,n}_{wildtype}(Q) + P^{p,n}_{mutation}(Q)]$. This process shifts the logistic regression towards the higher Qscore, allowing the classifier to accept a number of observed mismatches consistent with the prior expectation (Figure 1D). The fits were done independently for each position and possible mismatched nucleotides. To include deletions in this analysis (which do not have a Qscore assigned by the basecaller), we fixed their confidence value as the minimum of the Qscore of their direct nearest neighbors in the nucleotide sequence. This decision was inspired by the observation that the Qscore is correlated between consecutive nucleotides (Figure S3). Insertions were ignored as very few are expected (<1%) and it is not possible to obtain enough reads to fit all insertion possibilities. In applications where SINGLe is used to compute a high quality consensus, the original score of the inserted bases can be carried over. We also separated the fits for forward and reverse strands as the error rate per position is different in each case (see 'Are reverse and forward reads different in Nanopore Sequencing' in Supplementary material). All together, we obtained 13296=1662×4×2 regressions, one for each position of the gene (1662 bp), for each non-wild type nucleotide or deletion (4 possibilities in total for each position) and for the forward and reverse sense of sequencing. Please refer to the supplementary material for a brief discussion on 'How many Nanopore reads are needed to fit SINGLe?'.

The regressions were then used to re-score the mismatches in the mutant library: for each non wild type nucleotide aligned to the reference, we evaluated the Qscore reported by Guppy in the SINGLe fit obtained for that particular position and nucleotide and defined this value (p_{SINGLe}) as its probability of being correct p_{right}. For nucleotides read as wild type their Qscore are directly transformed into a p_{right} according to the Q values reported by Guppy: $p_{Guppy}=1-10^{-Qscore/10}$. Finally, to compute a consensus sequence we performed a weighted count of each nucleotide (and deletion) in each position (by summing p_{SINGLe} values instead of ones), and defined the consensus nucleotide as the one with higher value. Homopolymers regions were sorted so that the deletions are always at the 3' side on the forward strand. To compare, we also computed the variant consensus sequence using p_{Guppy} instead of p_{SINGLe} for nucleotides that do not match the wildtype, or by unweighted majority vote. In these cases, we did not sort the homopolymers region as it had a detrimental effect (see Figure S4).

Analysis

We tested SINGLe in a small set composed of seven variants of KlenTaq (named #1 to #7) which we obtained from independent bacterial clones and barcoded them using the nanopore barcoding kit before sequencing. Their true sequence was obtained by Sanger. Each contains 2-9 point mutations (supplementary table S1). Variants #1 to #5 only present nucleotide substitutions. Variant #6 has 7 substitutions, two of them in consecutive positions, and one deletion in a non homopolymer region. Variant #7 has 5 substitution and a deletion in a homopolymer ('GG' to 'G-').

Signal to noise ratio

A straightforward procedure to filter errors is to only trust read nucleotides which have a probability of being correct p_{right} higher than a threshold. In this section, we compared how this process performs over the seven KlenTaq variants when using either $p_{right}=p_{Guppy}$ or $p_{right}=p_{SINGLe}$.

We defined *signal* as the number of mismatches to wild type known to be actual mutations with p_{right} higher than the threshold (true positives), and *noise* as the number of those mismatches known to be a wild type nucleotide (false positives). The counts are also weighted by p_{right} for each nucleotide, i.e. instead of summing one for each occurrence, we summed the p_{right} associated with the nucleotide. Results are shown in Figure 2A (ROC curve), Figure 2B (signal-to-noise ratio) and Figure S5 (signal-to-noise ratio without weighting counts by

p_{right}). For all thresholds, SINGLe has a higher signal to noise ratio (up to 6 times higher, depending on the cut off), thus facilitating the identification of actual mutations. This remains true when no cut off is applied (cut off = 0). Notice that the ratio is different for both methods at cutoff zero because the counts are weighted by p_{right}.

Consensus sequences

We compared the consensus sequences of the 7 KlenTaq variants obtained by SINGLe and by other methods. Given several aligned nanopore reads of the same variant, the consensus can be computed by simple majority counting how many times each nucleotide was read in one position and keeping the most frequent. We call this method 'no weights'. The counts can be weighted by p_{Guppy} and choose the nucleotide with larger weighted count. We call this method 'Guppy'. Or the counts can be weighted by p_{SINGLe} ('SINGLe' method). We also computed the consensus using Nanopolish, which works directly on the raw electrical signal instead of the basecalled sequences in combination with HMMs to detect base variations [7], Medaka that counts nucleotides and uses a neural network to compare to a draft assembly and define mutations [9], and NextPolish which takes into account the neighbours of the nucleotide [15].

For each method, we computed the consensus independently for each variant using subsets of 3 to 50 sequences drawn randomly from all available reads, and repeated 50 times for each subset size. In Figure 2D, we plotted the success rate on the consensus computation, i.e. how many times the obtained consensus matches exactly the true sequence. For variants #1 to #6, the convergence is faster when using SINGLe weights: perfect consensuses are obtained for more than 90% of attempts starting from 5-7 sequences. Nanopolish reaches a 90% of success rate in 8 to 15 reads but fails for variant #1 (it systematically misses mutation G23A). Medaka requires more than 20 reads to reach 90% of success, and it does not converge for variants #4 and #5. NextPolish needs between 15 and 50 reads depending on the variant. Finally, using p_{guppy} or no weights has a poorer performance, not reaching 90% of success for 50 reads for any of the variants #1-#6. Notice that variant #6 has two consecutive mutations and they are properly detected by SINGLe. Variant #7 has a deletion in a homopolymer which is a challenging mutation to detect. In this case, SINGLe needs 35 reads to converge to the true sequence, still outperforming Medaka, Guppy and no weights (they need 45 reads). Only Nanopolish converges faster, with 15 reads.

Figure 2C shows the total number of mismatches (averaged over the 50 trials) reported by each method for variant #3, according to the number of reads used to compute the consensus. For any set size, SINGLe reports the closest number of true mutations compared to the other methods. In Figure S6 this is analysed in more detail: we classified the nucleotides in each consensus sequence according to true/false mutations and true/false wild types and observed that actually SINGLe detects true mutations with the fewest number of reads while keeping the lowest rate of false mutations. Nanopolish has a high rate of false wild types and underestimates the true mutations. Medaka has a similar behaviour and on top adds false mutations. Consensus using Guppy scores or no weight report a high number of false mutations, and so does NextPolish (though with a lower error rate).

Consensus in a large gene library

We also tested SINGLe on a large library of mutants of the Klen Taq gene obtained by epPCR, containing approximately 1200 variants with a mean mutation rate of 8muts/kB. The variants are unknown, but associated with a barcode of 36 nucleotides downstream the STOP codon. We sequenced the library using nanopore, grouped the reads according to the barcode and computed the consensus sequence for the reads associated to each barcode. We first confirmed the consistency of the different consensus methods on this library, with respect to the results obtained in previous sections of this article. We chose the most represented barcode (901 reads) and computed the success rate using Medaka and SINGLe for subsets of reads. As ground truth we used the consensus computed with all available reads, which is the same for all methods except Nanopolish (Table S2). As shown in Figure 3A, SINGLe returns the correct consensus sequence over 90% of the times when using at least 5 reads, while Medaka needs 15. Similar results were obtained for the other 9 most frequent barcodes using various methods for computing the consensus sequence (Table S2 and Figure S7).

We computed consensus sequences using SINGLe or Medaka for all the mutants in our library, provided that the identifying barcode is present at least four times in our dataset, and compared the number of mutations reported by both methods (Figure 3B). Out of the 1174 variants for which we had at least 10 reads, both methods report the same consensus in 1039 cases (89%). Among the 610 variants for which we had 6 to 10 reads, we obtained the same consensus sequence using Medaka or SINGLe only in 339 variants (56%), and for the 839 variants with less than 5 reads only 23% of the variants obtained the same consensus with both methods. For the other variants, independently of the number of available reads, Medaka returns more

mutations than SINGLe in the wide majority of cases. This is consistent with the observation in the previous section: Medaka tends to predict more mutations than there actually are. Less than 1% of all computed consensuses have more mutations by SINGLe than by Medaka.

If SINGLe improves the consensus sequences by reducing systematic errors from the basecalling, then the mutations predicted for a randomly mutated gene library should be homogeneously distributed along the sequence. In Figure 3C we show that this is the case. When there are more than 10 reads available in each cluster, SINGLe and Medaka predict mutations that are similarly distributed throughout the gene, as reflected by the skewness of the distribution of mutation counts (sk), around 0.8 in both cases. When there are only 6 to 10 reads available, Medaka tends to predict mutations on some preferred spots, increasing sk to 3.8. The effect is even larger for 5 or less reads: Medaka shows strong systematic errors (sk around 7), while SINGLe's reaches sk=1.3. In Figure S8, this same analysis is performed for other consensus calling methods and they all have a higher sk than SINGLe. Finally, we also compared the bias of the mutations in our library to the one reported by the manufacturer (Figure S9). All methods (except Nanopolish) had a correlation of .94 when sequences with more than 10 reads are used. Only SINGLe reaches this value for the sequences with 4 or 5 reads available.

Discussion

The relatively high error rate in single molecule nanopore sequencing limits some applications such as the analysis of libraries containing many different, but genetically similar, sequences. The approach we introduce here, SINGLe, leverages the fact that the sequencing errors in this case are partly systematic, as previously noted [4,12]. We accumulate many reads from the reference gene to build a sequence-specific error model that locally corrects for the sequencing biases. Applying this procedure on a set of seven variants of the KlenTaq gene with an average mutation rate of 3 bases/kb, we showed that correcting the confidence values provides a large increase in the signal to noise ratio. Consequently, the consensus calling returns 90% of perfect results from typically 5-7 reads. This implies a faster convergence than other methods currently used: nanopolish requires 8 to 15 reads, Medaka needs at least 20-50 reads and NextPolish between 15 and 50 reads to achieve a similar performance. Therefore, with SINGLe a lower burden on the sequencing throughput is taken to obtain true consensus sequences. Even when using very few reads to compute a consensus, SINGLe

detects the true mutations (high sensibility) without increasing the false mutations as much as other methods do (specificity). We also tested SINGLe in a library of approximately 1200 variants of KlenTaq, which are uniquely barcoded for clustering of the DNA strands. When many reads are available, Medaka and SINGLe report a similar number of mutations. But for variants with few reads, only 23% of the times both methods match and Medaka predicts more mutations in the remaining 77% of the cases. Furthermore, when we plotted the location of the mutations detected by each method, we observed that the ones returned by SINGLe are spread along the strand, while other methods present some hot spot positions. We interpret them as a consequence of the systematic errors produced by nanopore sequencing and that SINGLe overcomes.

SINGLe needs an expected average number of mutations in the test set. Here, our reference sequence was assumed to be perfect, and we could evaluate precisely the average mutation rate in the test set, because it originated from a controlled experimental mutagenesis protocol. In other situations it would be possible to use short read sequencing, for example Illumina, to evaluate this number. If the full sequence is submitted to short read high quality sequencing, it would even be possible to obtain more precise priors, for example specific to each position and nucleotide. Our approach would then be used to phase these statistical mutations to single long reads. An underlying assumption of our method is that the distribution of Qscore observed at a particular position for the wild type base reflects appropriately the distribution of Qscore that would be observed for a variant base at that position. This approximation is necessary since the error model is built from a single sequence and hence has a single ‘‘true” base per position. Fortunately, the difference in Qscore distributions for ‘‘true” versus ‘‘error” seems large enough for our method to perform well within that approximation.

SINGLe needs to characterise the sequencing errors done on an appropriate reference sequence. Because it applies a probabilistic approach on the erroneous nanopore reads, irrespective of what mechanism actually causes the systematic errors, it should not depend on the genes’ properties. On the other hand, SINGLe is limited to analyse variants which are close neighbours of the reference, and where mutations can be considered to be independent. We did not try to adapt the method to detect alterations beyond point replacements or deletions, which may require a more complex analysis pipeline. Encouragingly, variant 6 contained two contiguous mutations, and was properly analysed by our consensus approach. Finally, to our knowledge SINGLe is the first tool fully focused in analysing gene libraries sequenced by standard nanopore technology. Our approach provides a large improvement of signal to noise at very little experimental effort or

throughput reduction. The reads of the reference sequences, needed to train SINGLe, can be obtained simultaneously with the libraries using standard barcoding protocols and only use a small fraction of the sequencing throughput. There are no other modifications to the experimental protocol, and the computational error correction process can be simply added to any analysis pipeline after basecalling. SINGLe allows an exploitation of the sequencing throughput 10 to 20 times better than Medaka, and 4 times better than Nanopolish but with a better performance as we found that Nanopolish systematically misses some mutations. Therefore, SINGLe can be combined with experimental methods to obtain more accurate consensus sequences of large libraries of long genetic elements.

SINGLe is available as an R package that fits the errors on reads of a reference sequence, it then assigns p_{SINGLe} values on a library and a consensus sequence can be computed if a table with the barcodes in each read is provided. Its inputs are the .sam files obtained after a minimap2 alignment and samtool nucleotides count, the prior mutational rate and the reference sequence.

Methods

Samples preparation and sequencing

KlenTaq wildtype and seven variants: the wild type gene was amplified using a high fidelity PCR (Q5 polymerase from NEB) from a stored plasmid following NEB recommendations with primers GGGATTATTCTTTGGCGCTCAGCCAAT and ACCATGCGTCTGCTGCATGAAT. The mutants were obtained via epPCR using Agilent's kit GeneMorph II. We started from 1.1 nM of dam-methylated DNA, and we used the same primers as for the wild type. Thermocycling was performed as follows: 95°C for 2 min, followed by 25 cycles of [95°C for 30 sec + 65°C for 30 sec + 72°C for 2 min] and a final extension at 72°C for 10 min. In both cases, we digested the PCR product with DpnI (NEB) and purified it using columns (Macherey-Nagel). We put the genes in a pIVEX vector via Gibson assembly (NEB Hi-Fi DNA assembly) using 125ng of gene DNA (93ng for wild type gene), 100 ng of vector in an approximately 2:1 insert:vector molar ratio and incubated for 15 min at 50°C. We purified and concentrated DNA with a Zymo Research kit. We transformed the product into chemocompetent KRX bacteria. We spread them on a Petri dish with LB and Ampicillin (one plate for the library, another for the wild type). We incubated overnight at 37°C and the next day we randomly picked some clones from the library and from the wild type. We verified the presence of the plasmid via colony PCR using DreamTaq polymerase (Thermofisher). Positive clones were grown overnight in liquid LB with antibiotics and mini-prepped to obtain plasmid DNA. A fraction of the plasmid was used for high quality sequencing (Sanger sequencing). Another fraction of each clone was used for amplification by PCR with Q5

polymerase (NEB) in independent tubes. We used primers which included the minION barcodes adapters: ACTTGCCTGTCGCTCTATCTTCAGTGTGCTGGAATTCGCCCTTTTA and TTTCTGTTGGTGCTGATATTGCAGACCACAACGGTTTCCCTCTAGAAATA. Thermocycling was performed as follows: 98°C for 30sec, 23 cycles of [98°C for 10sec + 59°C for 30sec + 72°C for 1min], final extension at 72°C for 2min. We digested the product with Dpn1 (NEB), gel purified it using Macherey-Nagel kit. We proceeded to sequencing following standard Oxford Nanopore protocols. We used one barcode for wild type and one for each of the mutants 1 to 7. We used kits EXP-PCB001 for barcoding, SQK-LSK108 for ligation, EXP-LLB001 for flow cell loading. minION flow cell version was R9.4/FLO-MIN106, thus the sequencing was 1D. After data pre-processing (see section minION reads pre-processing), we had the following reads per variant: 716 (variant 1), 453 (variant 2), 645 (variant 3), 428 (variant 4), 422 (variant 5), 369 (variant 6), 358 (variant 7).

KlenTaq large library: Mutants were obtained via epPCR using Agilent's kit GeneMorph II. We started from 0.12ng of DNA (pIVEX vector containing KlenTaq gene). We used primers GCCAGTGTGCTGGAATTCGCCCTTTTATTAATG and CCCTCTAGAAATAATTTTGTTTAACTTTAAGAAGGAGATATACCATG. Thermocycling was performed as follows: 95°C for 2 min, followed by 30 cycles of [95°C for 30 sec + 58°C for 30 sec + 72°C for 2 min] and final extension at 72°C for 10 min. We digested the product with DpnI (NEB) for 1 hour and purified it using columns (Macherey-Nagel). We put the mutagenized genes in a pIVEX vector, previously amplified with primers that incorporated a random barcode downstream the STOP codon (aattccagcacactggcDHVBDHVBDHVBDHVBDHVBDHVBDHVBDHVBDHVBaagcccgaaaggaagctgag and ttaaagttaaacaaaattatttctagagggaaaccgttg). Previously a SalI site was added into the pIVEX vector, upstream the T7 promoter. We used NEB Hi-Fi DNA assembly using 100ng of vector DNA, 103 ng of insert DNA, in a 2:1 insert:vector molar ratio and incubated for 15 min at 50°C. We purified and concentrated DNA with a Zymo Research kit. We transformed the product into chemocompetent T7 Express lysY/Iq bacteria (NEB C3013I) via heat shock and spread them on a Petri dish with LB and Ampicillin. We incubated overnight at 37°C and obtained around 1000 CFU. We grew the library overnight at 37°C in liquid LB with Ampicillin and extracted the plasmid using Macheray Nagel kit. We digested the library plasmid with EcoRI-HF and SAlI-HF from NEB, in rCutSmart buffer at 37°C for 1h40min plus an inactivation step at 65°C for 20'. We added proteinase K and incubated at 37°C for 15 min. We gel purified the sample to keep the fragment containing the KlenTaq gene, and used Zymo research kit for concentration and purification of the DNA. We dialysed our sample using millipore membranes for 1h. For nanopore oxford sequencing we used a flongle (version FLO-FLG001), and SQK-LSK110 kit for sample preparation. We started with 110 fmol and loaded 50 fmol into the flow cell.

minION reads pre-processing

minION raw data was basecalled using ONT Guppy version 5.1 using model dna_r9.4.1_450bps_sup.cfg. We kept reads

which had a length of 1700 to 2100 nucleotides and a mean Qscore larger than 10 (except for the large library, for which we used a Qscore cut-off of 15). For the wild type and the seven variants we also used Guppy for demultiplexing. For the KlenTaq large library, we used a custom script made in the lab to group barcodes by exact match. Sequences were aligned to the reference wild type using minimap2 version 2.21 [16], using the options minimap2 -ax map-ont --sam-hit-only. We used samtools 1.7 [17] to create sorted bam files via the commands samtools view -S -b and samtools sort.

Consensus by Nanopolish

We used the scripts provided by Oxford Nanopore, multi_to_single_fast5 and single_to_multi_fast5 (version 4.0) to split fast5 files and reassemble them according to the associated barcode. We then used the commands nanopolish index ; samtools sort; samtools index; and nanopolish variants --consensus, according to the Nanopolish manual, to compute the consensus. Nanopolish version is 0.13.3.

Consensus by Medaka

We used racon (version 1.4) to polish our sequences and used it as an input in the consensus computation by medaka (version 1.4) which we run by the command line medaka_consensus with default parameters.

Consensus by NextPolish

We used NextPolish (version 1.4.1) with options task=best, rerun=3, genome= KlenTaq wild type sequence, and lgs_options -min_read_len 1k -max_depth 100.

Availability

Project name: SINGLe

Project home page: https://github.com/rocioespci/single, also available in bioconductor 10.18129/B9.bioc.single

Operating system: Platform independent

Programming language: R (>= 4.1).

Other requirements: Imports R packages Biostrings, BiocGenerics, dplyr, GenomicAlignments, IRanges, methods, reshape2, rlang, Rsamtools, stats, stringr, tidyr, utils. To create input files, minimap2 and samtools are required (or equivalent software to align data to reference and create a bam file).

Licence: MIT

Software is registered in scicrunch.org (identifier SINGLe, RRID:SCR_022810) and biotools (https://bio.tools/SINGLe).

Data availability

The data set supporting the results of this article are available in the European Nucleotide Archive repository, ERP135743, run ERR8778685 (nanopore reads of seven mutants and wild type) and ERR8778797 (nanopore reads of library), and in GigaDB [18] (Sanger sequencing for the seven mutants of Klen Taq, sequence of wild type KlenTaq gene, and the full code and processed data to reproduce the figures in the manuscript and supplementary material).

Funding

European Research Council, H2020 Marie Sklodowska-Curie Research and Innovation program, 845976, R Espada; European Research Council, Consolidator Grant No. 647275 ProFF, Y. Rondelez;

Competing interests

The authors declare that they have no competing interests.

List of abbreviations

epPCR: error-prone PCR

PCR: polymerase chain reaction

SINGLe: SNPs In Nanopore reads of Gene Libraries

SNP: single nucleotide polymorphisms

Qscore: Quality score

Sk: Skewness

Authors' contributions

RE and YR designed research and conceptualization. YR performed supervision of the research. RE, and ADM performed experimental research. RE, NK and YR perform methodology and formal analysis. RE wrote the

software. RE and YR wrote the manuscript, ADM and NK review and edited the manuscript.

References

1. Oxford Nanopore Technologies. [cited 1 Feb 2022]. Available: https://nanoporetech.com/

2. Sze MA, Schloss PD. The Impact of DNA Polymerase and Number of Rounds of Amplification in PCR on 16S rRNA Gene Sequence Data. mSphere. 2019;4. doi:10.1128/mSphere.00163-19

3. Thibodeau ML, O'Neill K, Dixon K, Reisle C, Mungall KL, Krzywinski M, et al. Improved structural variant interpretation for hereditary cancer susceptibility using long-read sequencing. Genet Med. 2020;22: 1892–1897.

4. Wang Y, Zhao Y, Bollas A, Wang Y, Au KF. Nanopore sequencing technology, bioinformatics and applications. Nature Biotechnology. 2021. pp. 1348–1365. doi:10.1038/s41587-021-01108-x

5. Sedlazeck FJ, Rescheneder P, Smolka M, Fang H, Nattestad M, von Haeseler A, et al. Accurate detection of complex structural variations using single-molecule sequencing. Nat Methods. 2018;15: 461–468.

6. Gong L, Wong C-H, Cheng W-C, Tjong H, Menghi F, Ngan CY, et al. Picky comprehensively detects high-resolution structural variants in nanopore long reads. Nat Methods. 2018;15: 455–460.

7. Loman NJ, Quick J, Simpson JT. A complete bacterial genome assembled de novo using only nanopore sequencing data. Nat Methods. 2015;12: 733–735.

8. Vaser R, Sović I, Nagarajan N, Šikić M. Fast and accurate de novo genome assembly from long uncorrected reads. Genome Res. 2017;27: 737–746.

9. Website. Available: https://nanoporetech.github.io/medaka

10. Chenhao Li, Kern Rei Chng, Esther Jia Hui Boey, Amanda Hui Qi Ng, Andreas Wilm, and Niranjan Nagarajan. Incseq: accurate single molecule reads using nanopore sequencing. Gigascience. 2016;5: s13742–016.

11. Karst SM, Ziels RM, Kirkegaard RH, Sørensen EA, McDonald D, Zhu Q, et al. High-accuracy long-read amplicon sequences using unique molecular identifiers with Nanopore or PacBio sequencing. Nat Methods. 2021;18: 165–169.

12. Krishnakumar R, Sinha A, Bird SW, Jayamohan H, Edwards HS, Schoeniger JS, et al. Systematic and stochastic influences on the performance of the MinION nanopore sequencer across a range of nucleotide bias. Sci Rep. 2018;8: 3159.

13. Huang Y-T, Liu P-Y, Shih P-W. Homopolish: a method for the removal of systematic errors in nanopore sequencing by homologous polishing. Genome Biol. 2021;22: 95.

14. Manual available in https://www.agilent.com/cs/library/usermanuals/Public/200550.pdf

15. Hu J, Fan J, Sun Z, Liu S. NextPolish: a fast and efficient genome polishing tool for long-read assembly. Bioinformatics. 2020;36: 2253–2255.

16. Li H. Minimap2: pairwise alignment for nucleotide sequences. Bioinformatics. 2018;34: 3094–3100.

17. Li H, Handsaker B, Wysoker A, Fennell T, Ruan J, Homer N, et al. The Sequence Alignment/Map format and SAMtools. Bioinformatics. 2009;25: 2078–2079.

18. Espada R; Dramé-Maigné A; Zarevski N; Rondelez Y. (2022): Supporting data for "Accurate gene consensus at low nanopore coverage" GigaScience Database. http://dx.doi.org/10.5524/102265

Figures' legends

Figure 1: A Distribution of Qscore returned by Guppy basecaller for each nucleotide on the sequencing of the wild type KlenTaq gene, classified as correct reads (green) and errors (red). **B** Nanopore sequencing errors per position on reads of the wild type KlenTaq gene. Colours indicate the nucleotide reported. Wild type (correct) nucleotides are not plotted. Only positions 100 to 150 are shown here. An equivalent plot for all positions is available in Figure S2. **C** Example of logistic regression over reads of a known wild type sequence. Black dots are the proportion of correct nucleotides with a given Qscore in one position and in comparison to one possible error. Dashed red line is the logistic regression performed. **D** Same plots as C after data was weighted according to the prior probabilities. Blue line is p_{SINGLe}.

Figure 2: A True positive rate (TPR) vs false positive rate (FPR) (or ROC curve) for SINGLe (blue) and Guppy (red). **B** Signal to noise ratio when classifying mismatches as mutations when they reach p_{right} indicated on the x axis, for SINGLe (blue) and Guppy (red). **C** Mean number of mismatches between the consensus of variant #3 (4 sub) and the wild type sequence when using a subset of sequences (x axis) to compute the consensus. Colours indicate the method used for computing the consensus. **D** Success rate on obtaining the true sequence when computing the consensus with a subset of reads. The size of the subset used is indicated on the x axis. The success rate is computed over 50 different consensus on independent subsets of reads. The colours indicate the method used for the consensus calling. Each panel has one of the 7 variants analysed, and the number of substitutions (sub) and deletions (del) are indicated on top.

Figure 3: A For the most frequent variant in the library, success rate of the consensus computed from subsets of available reads using Medaka (green) or SINGLe (blue). **B** Comparison of the consensus sequences returned by Medaka and SINGLe. We splitted the results by the number of reads available for each variant (less than 5, 6 to 10, and more than 10). The bars on the middle indicate the proportion of variants whose consensus sequence is the same either computed by Medaka or by SINGLe. The left bar indicates the proportion of variants whose consensus sequences have more mutations when computed with Medaka than when computed by SINGLe, and the right bar the opposite. **C** Mismatches to wild type on the consensus computed by Medaka (upper panels) and SINGLe (lower panels) by position, and classified according to the number of reads available for the consensus computation (label on top). To quantify the systematicity of the mismatches, we used the skewness

(sk) of the distribution of counts.

Figure 1

Click here to access/download;Figu

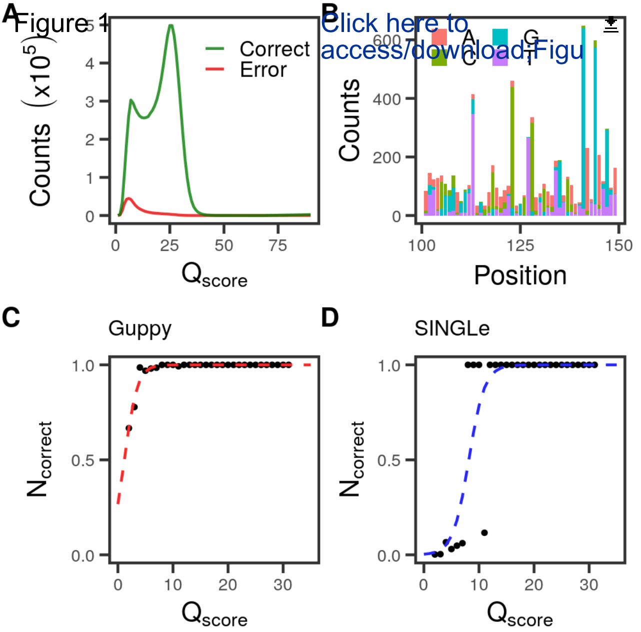

Figure 2

Click here to access/download;Figure;Figure2.pdf

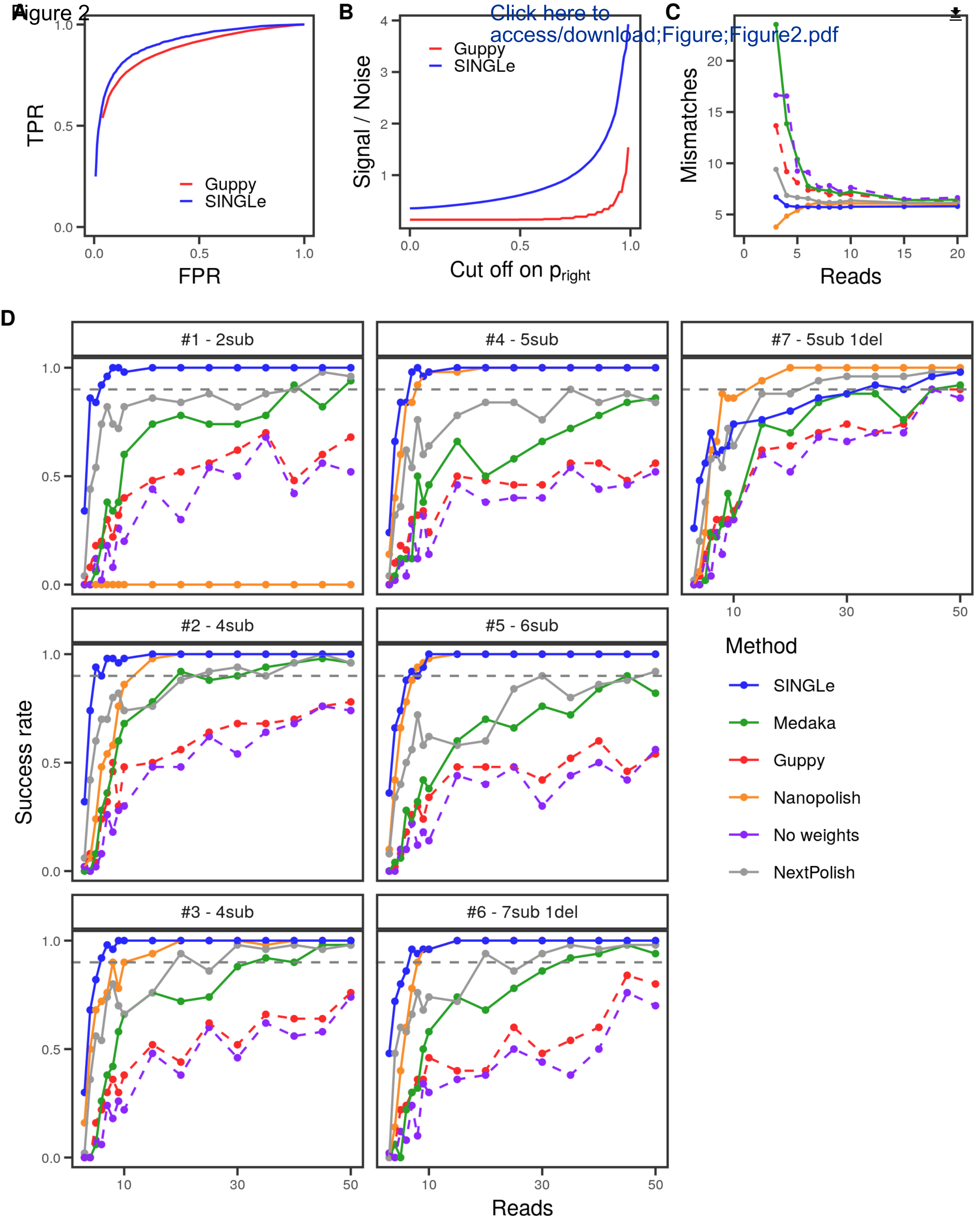

Figure 3

Click here to access/download;Figure;Figure3.pdf

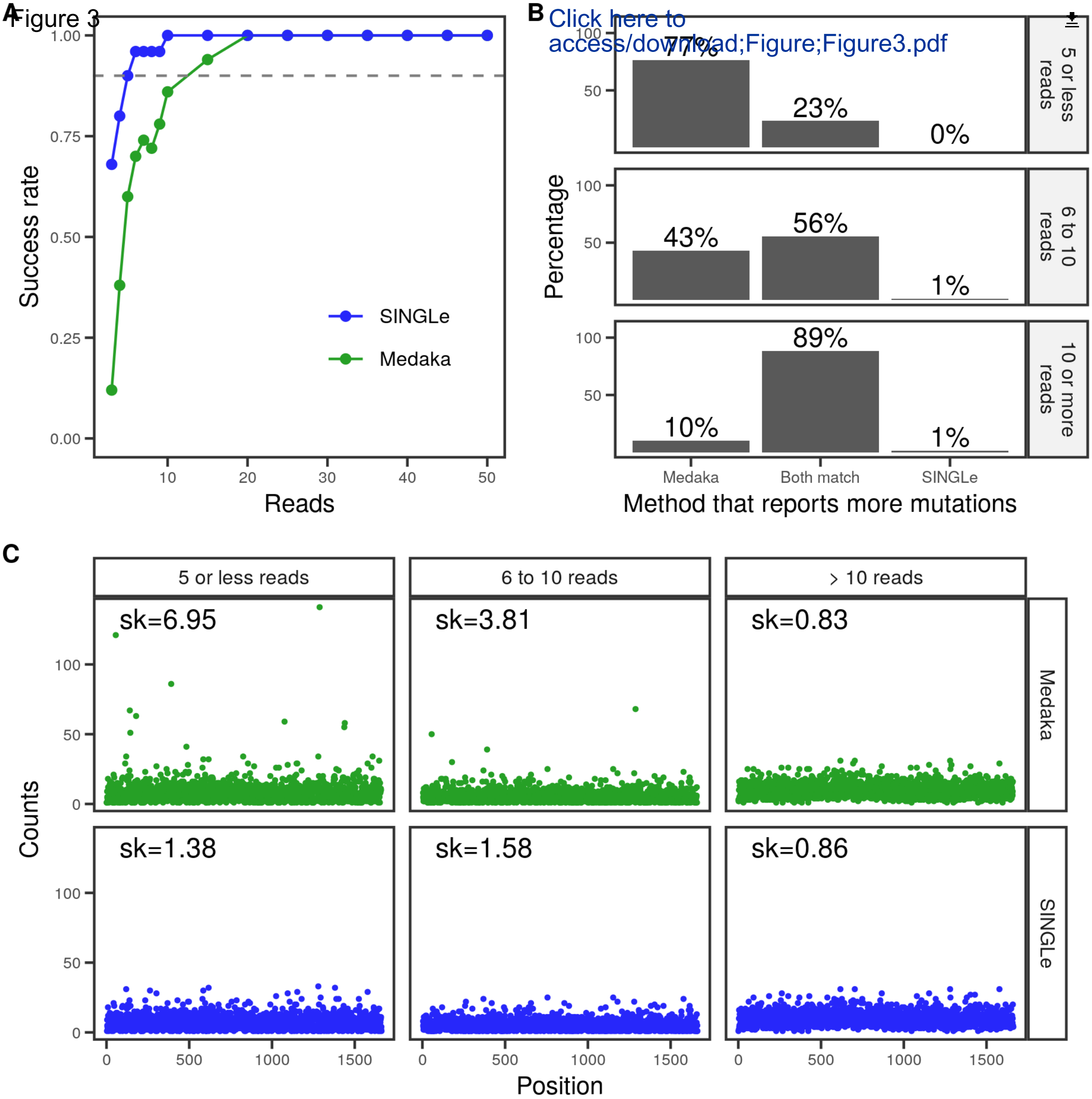

Click here to access/download
Supplementary Material
SINGLe_Supplementary_reviewed.pdf
