# Supplementary material for: Accurate gene consensus at low nanopore coverage
Source: Gigascience. 2022 Nov 9;11:giac102. doi: 10.1093/gigascience/giac102 (PMC9646519; doi:10.1093/gigascience/giac102)
Supplement: giac102_Supplemental_File [file giac102_supplemental_file.pdf]

Accurate gene consensus at low nanopore coverage

Espada Rocío, Zarevski Nikola, Dramé-Maigné Adèle,Rondelez Yannick

Supplementary information

ATGCGTCTGCTGCATGAATTTGGTCTGCTGGAAAGCCCGAAAGCACTGGAAGAAGCCCCTTGGCCTCCGCCTGAAGGTGCA

TTTGTTGGTTTTGTTCTGAGCCGTAAAGAACCGATGTGGGCTGATCTGCTGGCACTGGCAGCAGCACGTGGTGGTCGTGTTC

ATCGTGCACCGGAACCGTATAAAGCACTGCGTGATCTGAAAGAAGCACGCGGACTGCTGGCAAAAGATCTGAGCGTTCTGG

CCCTGCGTGAAGGTCTGGGTCTGCCTCCGGGTGATGATCCGATGCTGCTGGCATATCTGCTGGATCCGAGCAATACCACACC

GGAAGGTGTTGCACGTCGTTATGGTGGTGAATGGACCGAAGAAGCAGGCGAACGCGCAGCACTGAGCGAACGTCTGTTTG

CAAATCTGTGGGGTCGTCTGGAAGGTGAAGAACGTCTGCTGTGGCTGTATCGTGAAGTTGAACGTCCGCTGAGCGCAGTTC

TGGCACACATGGAAGCAACCGGTGTTCGTCTGGATGTTGCCTATCTGCGTGCACTGAGCCTGGAAGTTGCAGAAGAAATTG

CACGCCTGGAAGCAGAAGTTTTTCGTCTGGCAGGTCATCCGTTTAATCTGAATAGCCGTGATCAGCTGGAACGTGTTCTGTT

TGATGAACTGGGCCTGCCTGCAATTGGTAAAACCGAAAAAACCGGTAAACGTAGCACCAGCGCAGCCGTTCTGGAAGCCCT

GCGCGAAGCACATCCGATTGTTGAAAAAATTCTGCAGTATCGCGAACTGACCAAACTGAAAAGCACCTATATCGATCCGCTG

CCGGATCTGATTCATCCGCGTACCGGTCGTCTGCATACCCGTTTTAATCAGACCGCAACCGCCACCGGTCGCCTGAGCAGCA

GCGATCCGAATCTGCAGAATATTCCGGTTCGTACACCGCTGGGTCAGCGTATTCGTCGTGCATTTATTGCCGAAGAAGGTTGG

CTGCTGGTTGCACTGGATTATAGCCAGATTGAACTGCGTGTTCTGGCGCATCTGAGCGGTGATAAAAATCTGATTCGTGTTTT

TCAAGAGGGTCGCGATATTCATACCGAAACCGCAAGCTGGATGTTTGGTGTTCCGCGTGAAGCAGTTGATCCGCTGATGCGT

CGTGCAGCAAAAACCATTAACTTTGGTGTGCTGTATGGTATGAGCGCACATCGTCTGAGCCAAGAACTGGCAATTCCGTATG

AAGAAGCACAGGCCTTTATTGAACGTTATTTTCAGAGCTTTCCGAAAGTTCGTGCATGGCTGAAAAAAACCCTGGAAGAGG

GACGTCGTCGTGGTTATGTTGAAACCCTGTTTGGTCGTCGTCGCTATGTTCCGGATCTGGAAGCACGTGTTAAAAGCGTTCG

TGAAGCCGCAGAACGTATGGCCTTTAATATGCCGGTTCAGGGCACCGCAGCAGATCTGATGAAACTGGCCATGGTTAAACTG

TTTCCACGGCTGGAAGAAATGGGTGCACGTATGCTGCTGCAGGTTCATGATGAGCTGGTGCTGGAAGCGCCTAAAGAACGT

GCAGAAGCCGTTGCCCGTCTGGCCAAAGAAGTTATGGAAGGCGTTTATCCGCTGGCAGTTCCGCTGGAAGTGGAAGTTGG

TATTGGTGAAGATTGGCTGAGCGCCAAAGAA

Sequence S1: Nucleotide sequence of the wild type gene of KlenTaq.

Variant	Mutations
#1 - 2sub	G23A C245T
#2 - 4sub	C867G G1120A C1214T G1316A
#3 - 4sub	T425A G846A C851T G1403A
#4 - 5sub	G94C A378G A905G G1023T A1381T
#5 - 6sub	G303A G376A C517G T857A T1056A G1550A
#6 - 7sub 1del	A41T G331T G349A C772T C879T **C1474A C1475T** **G1530-**
#7 - 5sub 1del	G307A **G490-** T659A T675A G993A A1554G

Table S1: Mutations on the seven KlenTaq variants tested. The two consecutive mutations in variant #6 are highlighted in bold font, and deletions are indicated in grey.

Barcode	Reads	Mutations
AAATTACGAAACTTAGGTGGAAATGCAGATATTAG	515	G223A G238A C379T A395G C592T G633A T647A T749C T750A A860T A877T G926A C929T T948C T1017A T1056C A1102T G1123A G1212T A1548G G1550A G1639T C1655T
ACGCGCACGTAGACAGATACGAAGGCCGACAGGAG	505	G139A G174T C421T T525G A585G C592T T594A G676- G831A G881A
ACGGGACCGCATAAACGTATAAAGAAGTAAAGTCG	503	T60C G62- (G62T) T86G A185G A186T T242C A369T G416- T464A A500T C804T C907A T1079G C1174A C1239T
ATAGTACCATGTATCTATGGTTAGTCTATACTA	527	A428- G890C G978A
GAGTTAAGGTGCAAGGGTATATGTTCCCATGTG	675	T161C T309A A339G G499T T596A T630A A737T A758G C934T A1152- A1206G G1307- A1308- C1309- T1317C C1394T A1543-
GTCCACGCAAACTCATGTGGTCGGACCTAACCATG	559	C10G G142T A186T G243- G263C C371T A377G T471C T512A T617A C625A A673T T702A A870G T887C C1115T T1165G T1321C C1433G C1475G T1586A
GTCCATGTAAGCACAGGTCCTACTTAAGATGTTAG	491	T15C T126C A186T T596A G775T T857A C869A C879T A957G G1068A C1081A G1104A G1116A T1266A C1345T T1448A T1454A A1580T
GTCTGCCCACGGATCTGTGCATAGATACATGCACC	505	G257A G275A G360C G498A C503T G593C T819G T1019A A1223T T1448C C1468G G1533A A1619T
TAGCTAGTGAGTAGTATATGAGTGCGTGAAGGCA	608	A18T T302A T497A T533C G562T C581T G636A G643A G676A G854A C1372T T1418C A1437G A1578T
TCAGTTATTACGTCCTGCGGACAGTAAGGTCCGAG	901	G234A T258G G261- A369T G643C C668T A756T A1153G C1280T C1332T C1383T C1457T A1578G C1615A T1616C (T1616-)
Missed (or alternative) by nanopolish	Extra (or alternative) by Medaka	Missed by SINGLe

Table S2: Consensus sequence for the ten most frequent variants in the KlenTaq library using all available reads. The mutations missed by Nanopolish are in orange, and between parentheses the mutation that differ: in the last sequence, instead of detecting T1616C as the other methods, Nanopolish returns T1616-. In blue, the mutation that is only detected by Medaka, and between parentheses the mutation that differs from other methods. Finally in pink, the deletion that SINGLe does not detect.

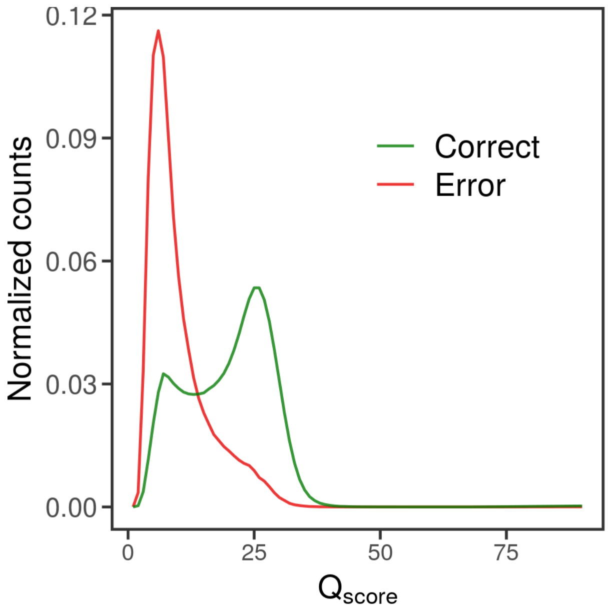

Figure S1: Normalized distribution of nucleotide's Qscore returned by Guppy basecaller during the sequencing of the wild type gene of KlenTaq, classified as correct reads (green) and errors (red).

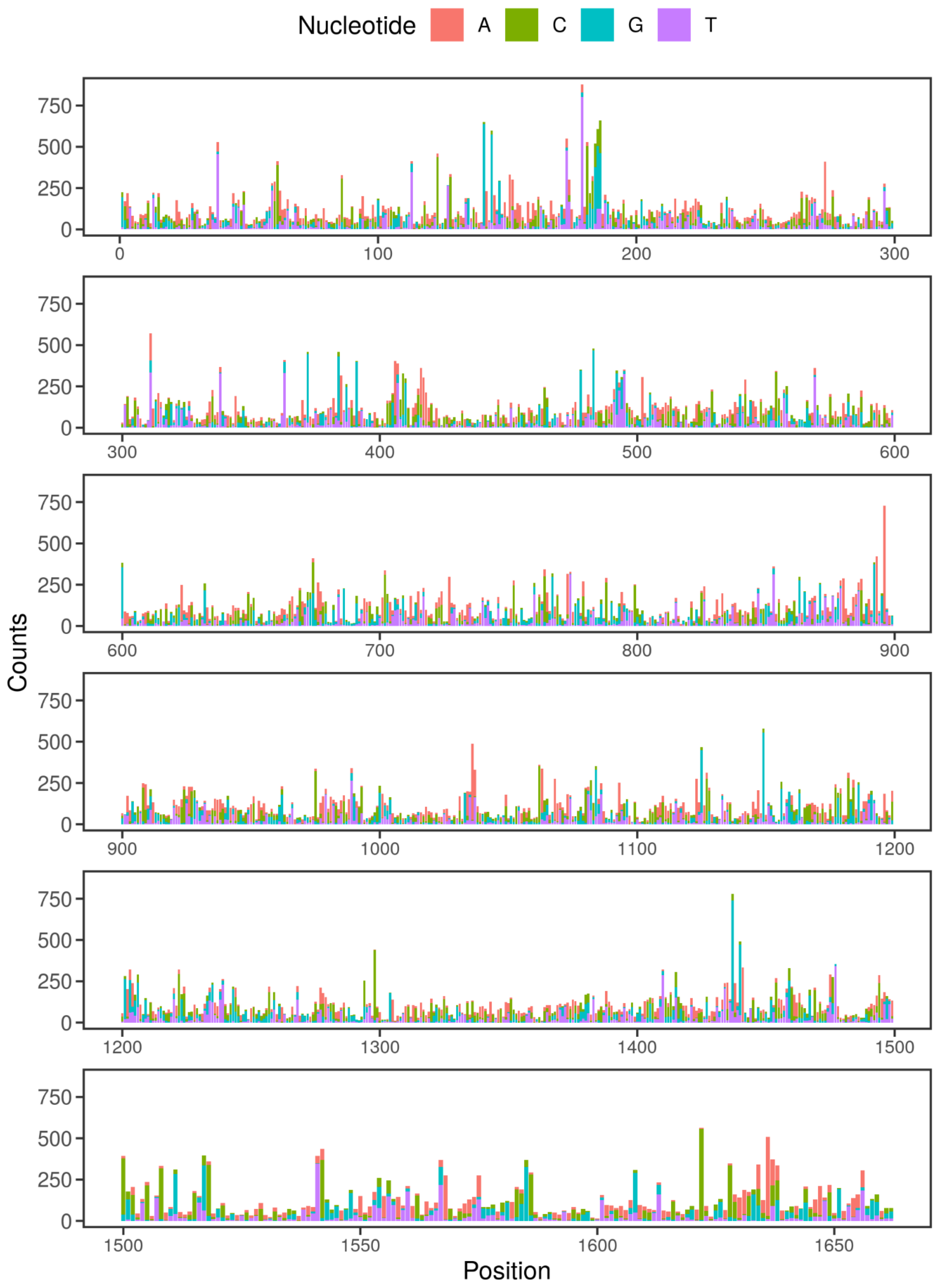

Figure S2: Errors per position produced by guppy basecaller on nanopore reads of the wild type gene of KlenTaq. Colours indicate the nucleotide informed by the basecaller. Wild type (correct) nucleotides are not shown.

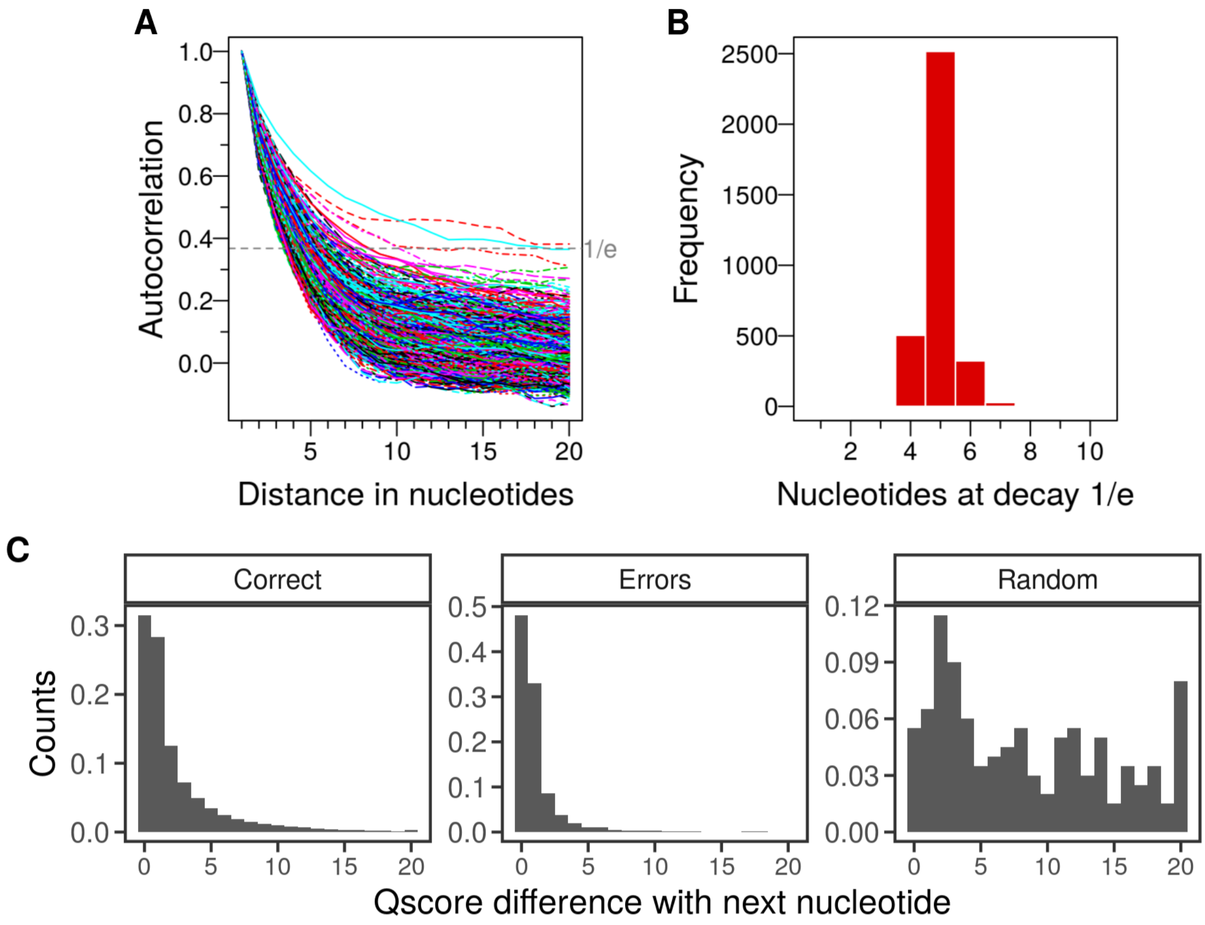

Figure S3: A Autocorrelation function of the Qscore on a DNA strand read by nanopore sequencing. Each line corresponds to an independent read. All of them belong to the sequencing of the wild type gene of KlenTaq. **B** Histogram of the nucleotide in which the autocorrelation decays to 1/e. Values over 10 are not shown. **C** Distribution of the difference of the Qscore values between a nucleotide and the following in a read, separated by correct reads (left) and errors (middle). This was computed on reads of the forward strand of variant 2. Positions related to the true mutations and deletions were excluded. As a control, we computed the difference of the Qscores for random pairs of nucleotides.

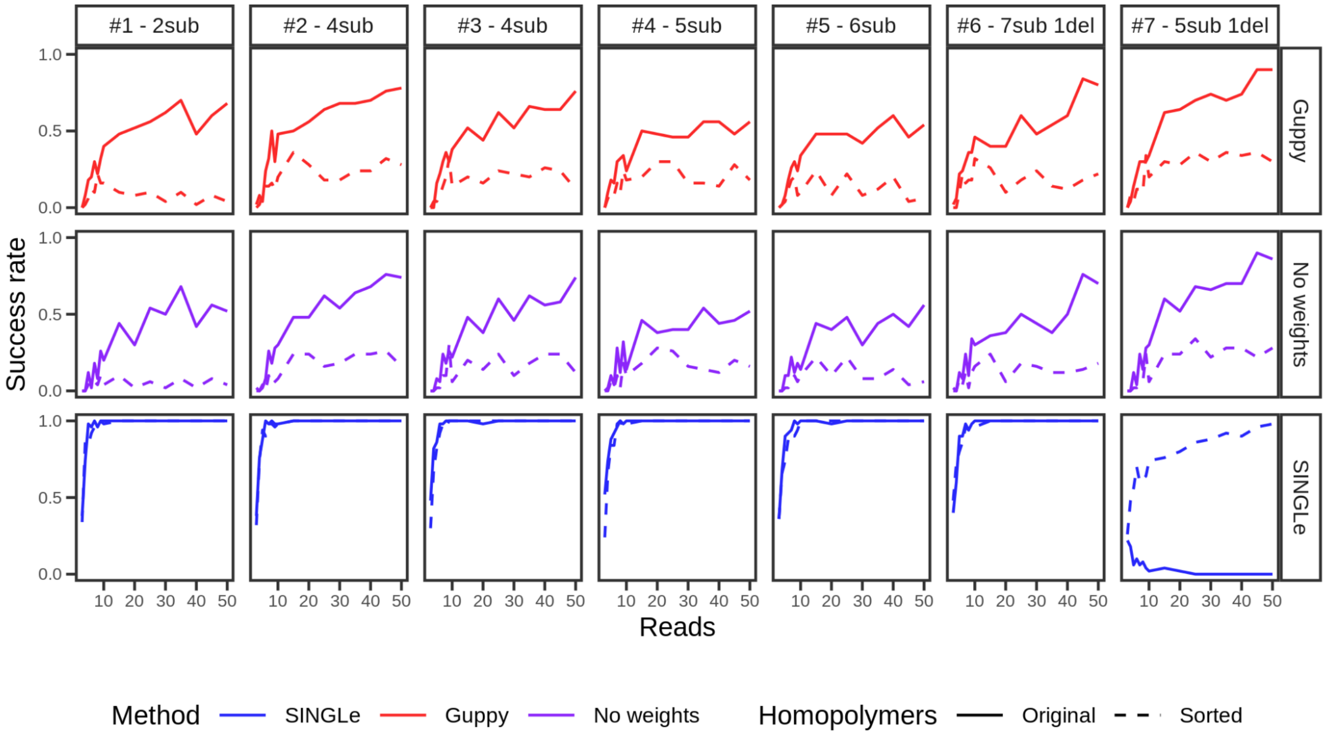

Figure S4: Success rate of consensus computation when sorting or not the nucleotides within homopolymers. We computed the consensus using the alignment as returned by minimap2. In this case, when there is a deletion in a homopolymer, it is assigned to any position within the homopolymer positions (solid lines). We also tested sorting the homopolymers, meaning that we always locate the gaps at the 3' side of the forward strand of the homopolymer region (dashed lines). This had a detrimental effect in the consensus computed with p_{Guppy} (red curves) and for the unweighted consensus (purple curves) on the seven mutants analysed. Accumulating the deletions at the end of the homopolymers led to a higher detection of deletions even in non mutated homopolymers (due to sequencing artefacts), whereas, without sorting, these false deletions are averaged out among several positions. In the case of SINGLe, sorting homopolymers has a minimal effect on mutants with no true deletion (variants #1-#5) or with a deletion in a non-homopolymer region (variant #6), while it drastically improves its performance for the detection of a deletion within a homopolymer (variant #7,bottom left panel).

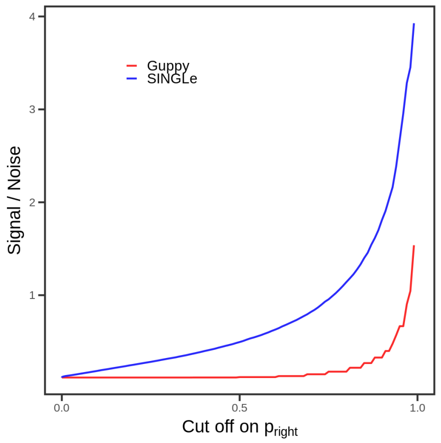

Figure S5: Signal to noise ratio, without weighting the counts by p_{right}.

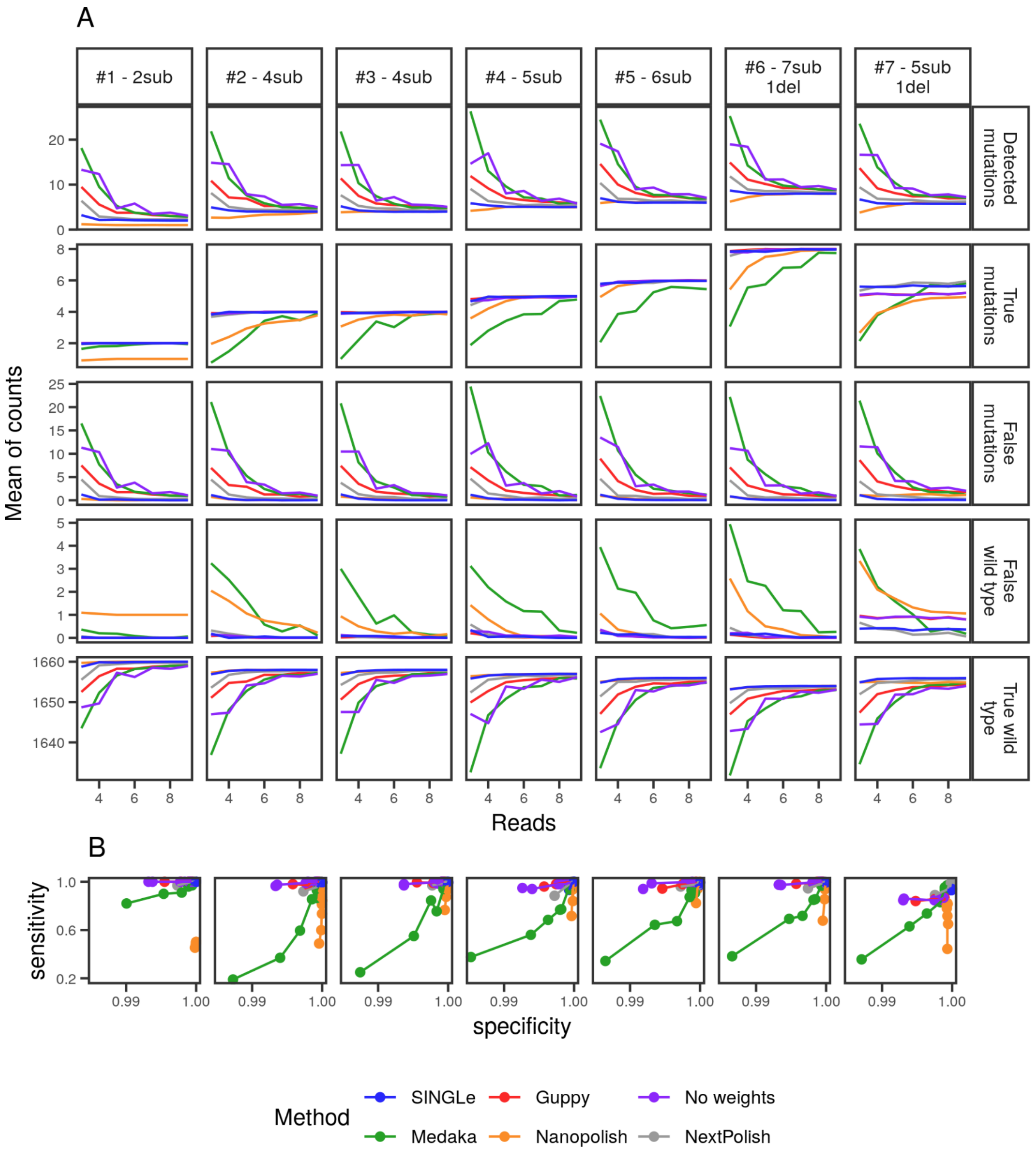

Figure S6: A Classification of nucleotides in the consensus computed from subsets of available reads, using different methods. The panels are organised in columns according to the KlenTaq variant. In this plot, we refer to the mutations as positives and the wild type nucleotides as negatives. The first row shows the total number of mutations detected. The second one, from these mutations, how many are true mutations (true positives). The third row, how many predicted mutations are actually errors (false positives). The fourth row shows how many nucleotides are wrongly reported as wild type (false negatives), and the last row how many wild type nucleotides were properly detected (true negatives). In all cases, the counts on the y axis are an average computed on the 50 consensus obtained with different subsets of nanopore reads. The colours indicate the method used for computing the consensus, as indicated in the legend at the bottom. **B** For each variant (column panels) and method (colour code in legend at the bottom), we computed the sensitivity as true mutations / (true mutations + false wild type) and the specificity as true wild type/ (true wild type + false mutations).

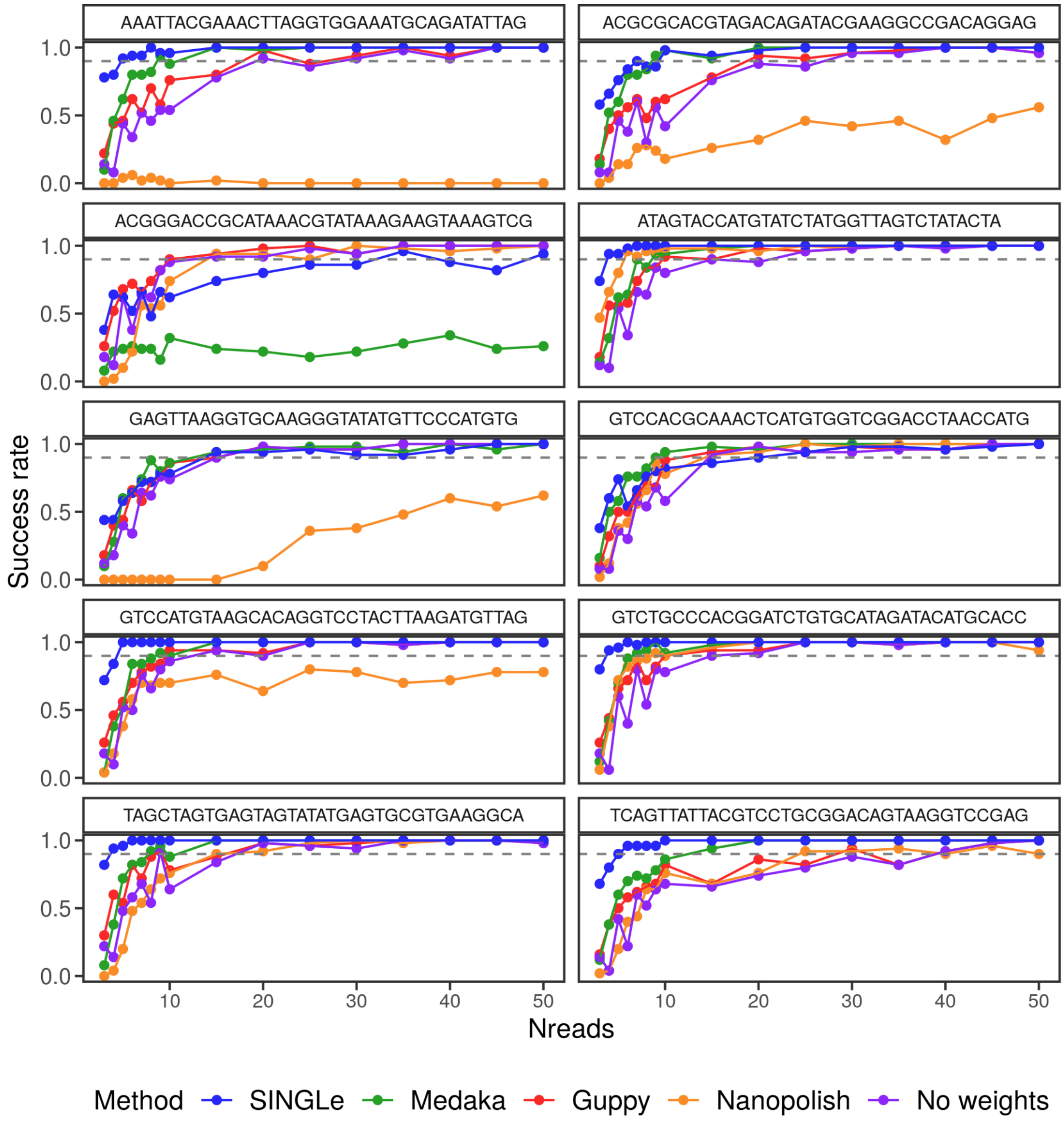

Figure S7: For the ten most frequent variants in the KlenTaq library (each named after their DNA barcode on the top of each panel), success rate of the consensus computation from subsets of available reads.

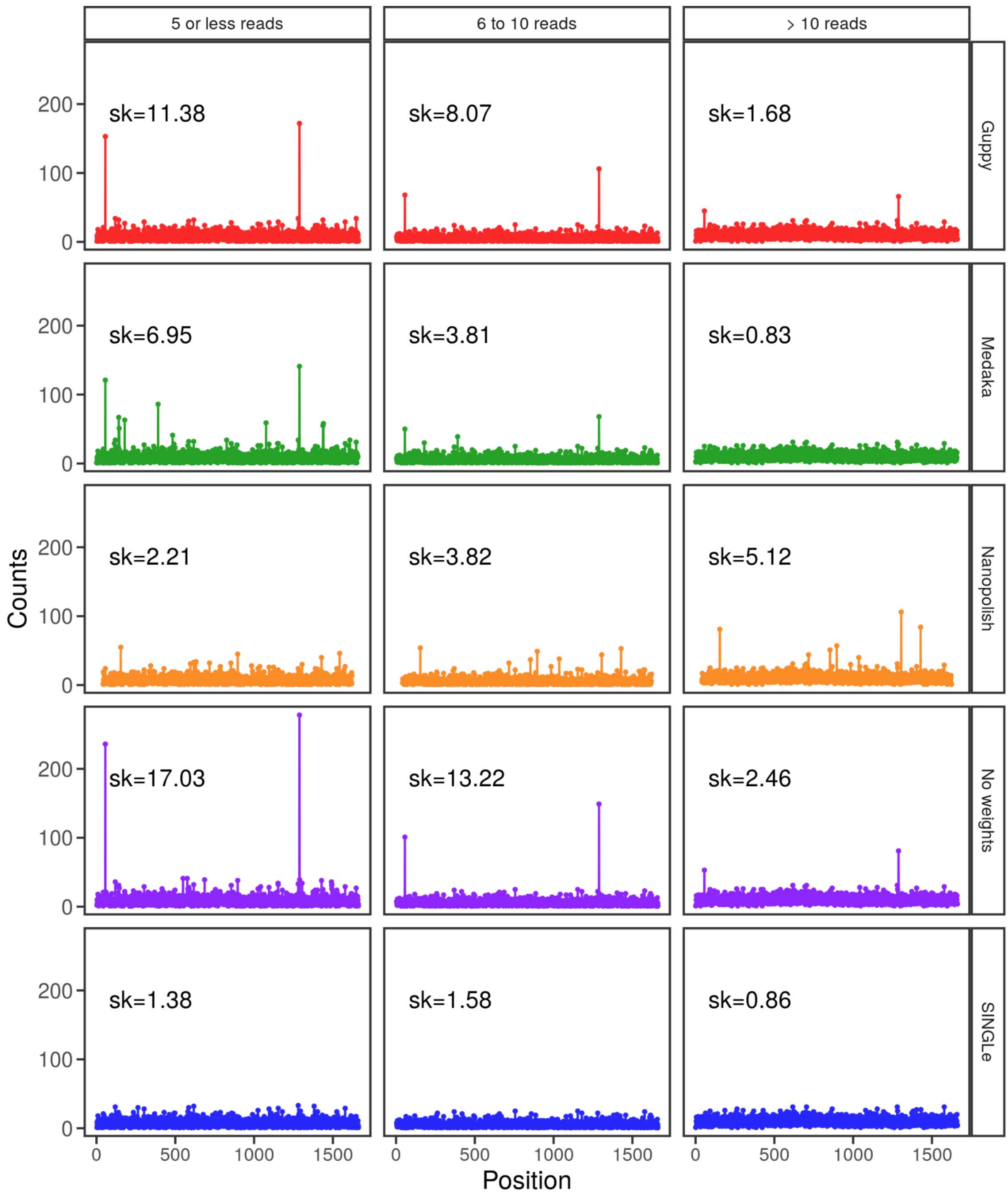

Figure S8: Mismatches to wild type on the consensus computed by all tested methods (label on the right) by position, and classified according to the number of reads available for the consensus computation (label on top). In all cases, and specially when only few sequences are available, SINGLe has a lower skewness of the mismatches detected. This reflects that mutations are randomly distributed along the sequence, as expected from epPCR.

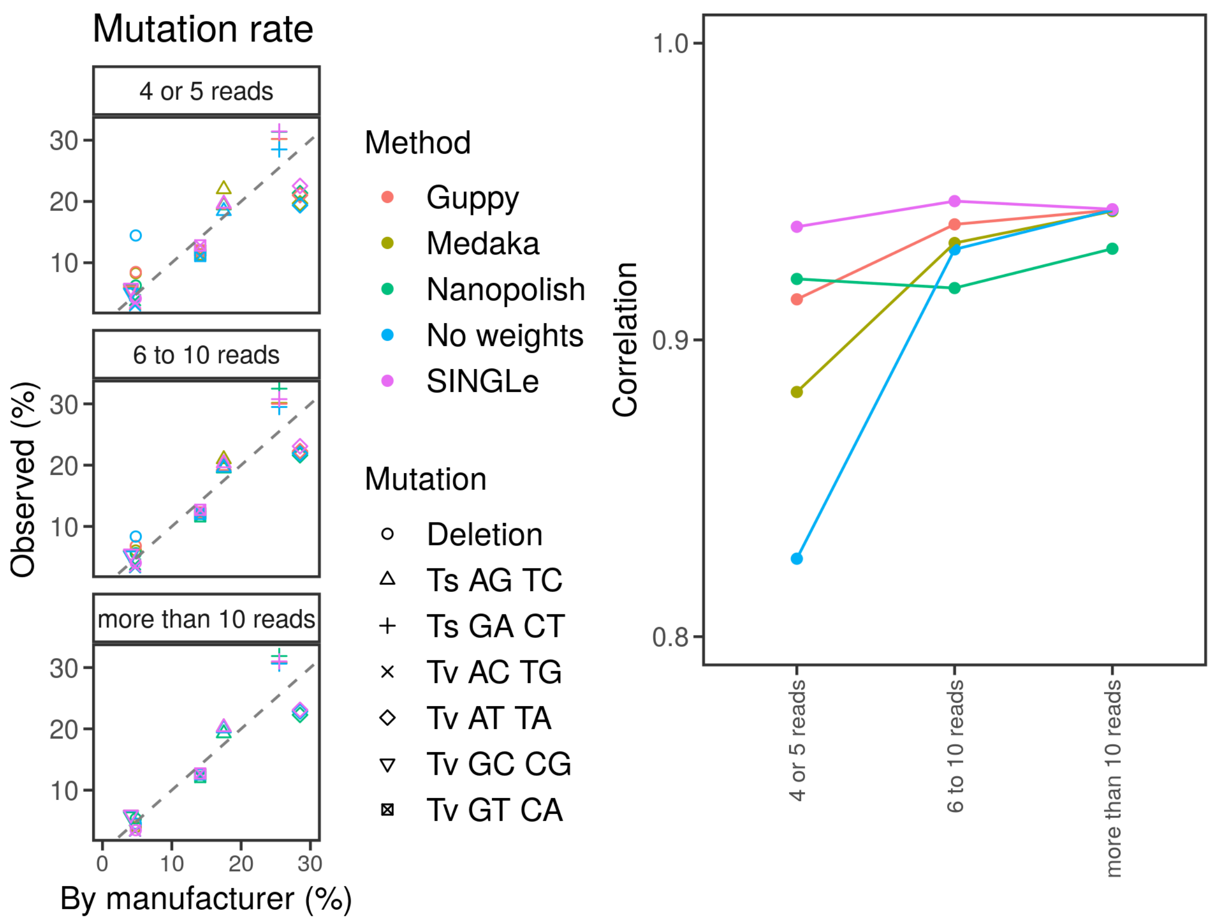

Figure S9: Comparison of mutational bias reported by the manufacturer of the epPCR kit, and the obtained mutations on the reads of the large KlenTaq library, according to the method used to compute consensus (colour code in the legend). On the left, on the x axis the percentage of mutations by type (shape) reported by the manufacturer vs observed on the y axis. We splitted the plot into three panels according to the available reads to compute the consensus. On the right panel, the correlation of observed and expected mutational rate obtained from the plots on the left, for each method (colour), and according to the number of available reads (x axis).

How many Nanopore reads are needed to fit SINGLe?

A rough estimation of the number of reads can be done from the average mutation rate. In the presented example, the error rate computed on the reads of the wild type is 3.97%. We measured around 3000 reads of the wild type sequence (in each strand sense). This means that the average number of errors per case (strand, position and nucleotide -or deletion) should be around 30. Nevertheless, the distribution of errors is not homogeneous (Fig. S2). In our dataset there are some cases with over 100 errors, and some with only 1 or 2. Making it harder to estimate the number of reads of the wild type sequence to obtain a good fit for every position and nucleotide.

We looked at this variability by plotting for each case the counts (C) of errors used to fit vs the quality of the fit (Q) calculated as 1- deviance of null model / deviance of model (parameters returned by the glm function in R), in Figure S10. There is some relation between these two variables, but we looked closer at particular examples (denoted in colored points). We chose combinations of nucleotide-position that are actual mutations in the small set of KlenTaq mutants (ie, they have been evaluated for consensus calling in the main manuscript).

On one extreme, we have the example position 879, nucleotide T, reverse strand, in which there is a lot of data (195 data points) and therefore the quality of the fit is very good (0.81). A good fit can still be obtained with a few counts. This is the case of position 303, nucleotide A and reverse strand that only has 7 data points but the quality of the fit is 0.98. The opposite is also true: the low quality of the fit is not always due to low number of observations as the measurements might not be distributed in a sigmoid curve, as the position 1316, nucleotide A, forward strand. In this case, the fit could probably be improved by ignoring outliers. Finally, there are cases in which there are very low counts and the resulting Q is very low. This is the case for position 331, nucleotide T, forward strand: in over 3000 reads of the wild type sequence, only once there was an error. This means that the reads in this particular position are accurate. Nevertheless, SINGLe fits a line close to 1 for any Qscore, meaning that any observation done on the mutants in this position will have a high p_{SINGLe}, consistently with the low error rate.

The lower the error rate, the more reads are needed to obtain enough statistics. But if the error rate is too low, and because there is always the majority of correct reads, SINGLe does not have a detrimental effect.

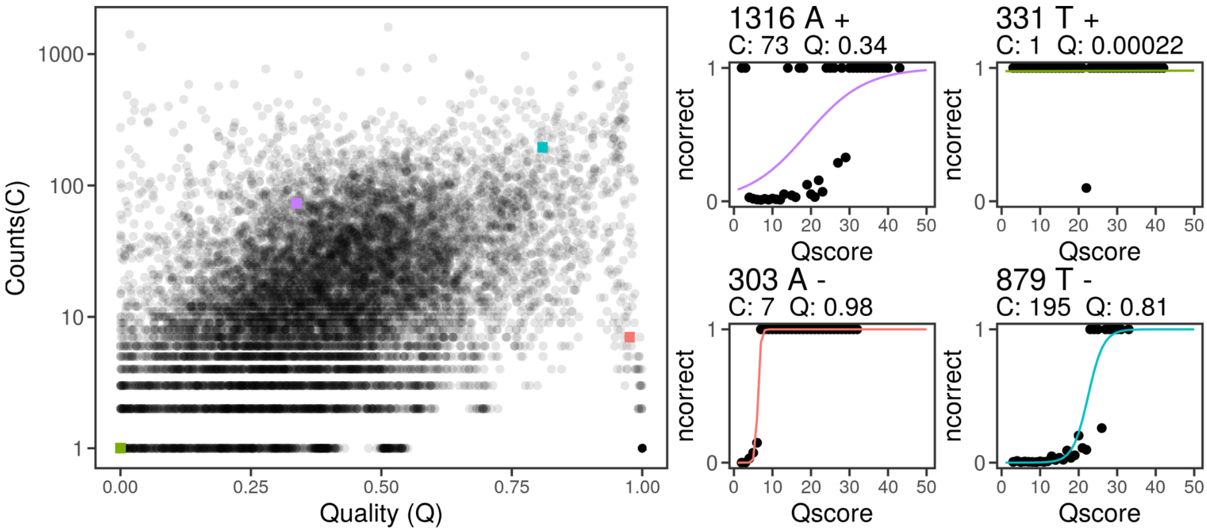

Figure S10: How many errors were detected in a particular case (position, nucleotide, strand) and used for fitting (Counts C) vs the quality of the fit (Q). The y-axis is in log scale. Some examples are marked in color in this panel and the fit is plotted on the right. The upper labels indicate position, nucleotide, strand (+ forward, - reverse), counts and quality of the fit. The selected examples are cases of mutations that appear in the small set of KlenTaq mutants.

Are reverse and forward reads different in Nanopore sequencing?

For double stranded DNA, nanopore sequencers read any of both strands. Here we refer to the forward strand to the one in the sense of the gene, and reverse strand to the other one. We explored if the reads of the wild type gene of KlenTaq done on the forward or reverse strand have different error patterns in Nanopore sequencing. First we noticed that the Qscore distributions are similar for reverse and forward reads (Fig. S11A). Secondly, we computed the percentage of errors for each reading direction separately. We obtained an error rate of 3.8% for the forward reads vs 4.06% for the reverse reads.
We then looked at the error rates per position, and plotted them one sense against the other one (Fig. S11B). We observed that these values do not correlate, meaning that the error rate in a given position is different if reading forward or reverse strands of DNA. Finally, we verified that this leads to different results on the logistic regression fit for the forward and reverse strand, as shown in some examples in Fig. S11C.

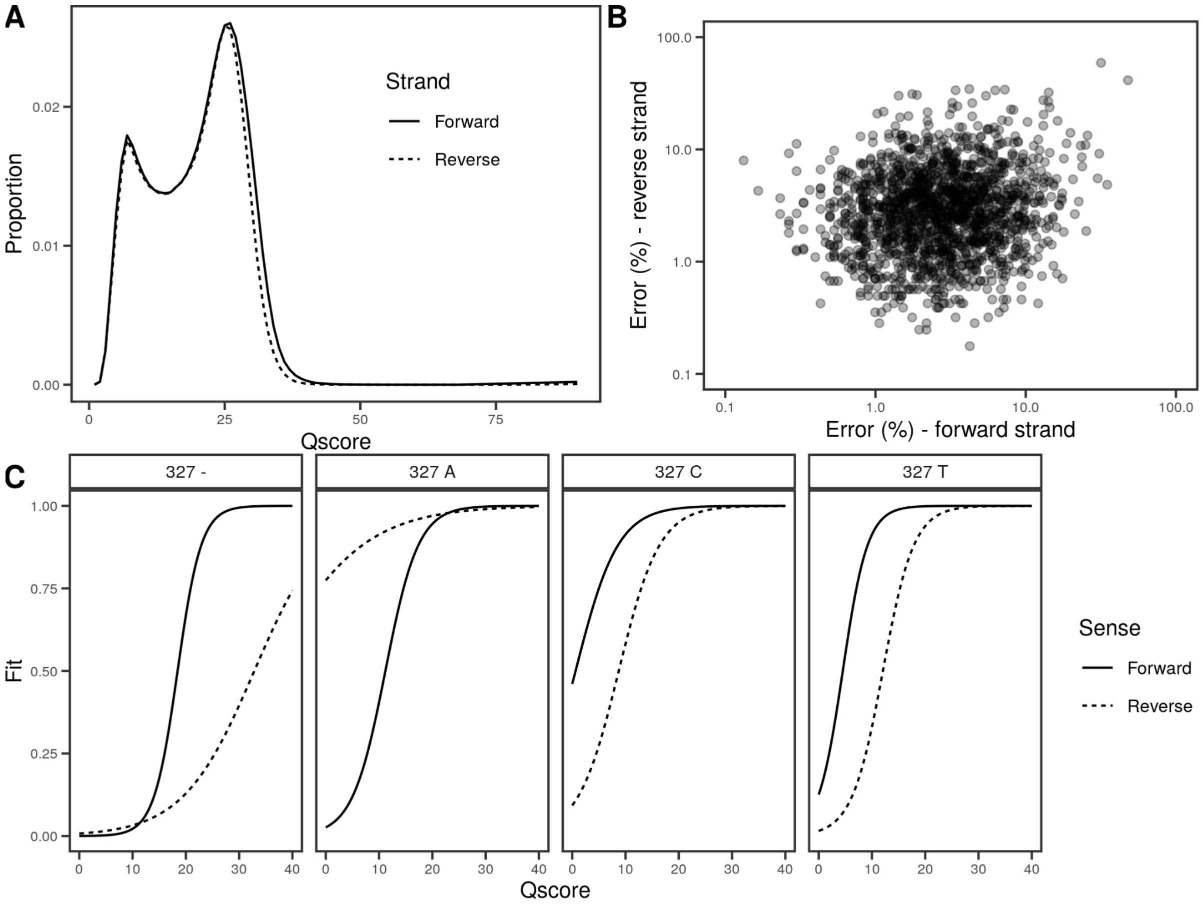

Figure S11: Analysis on the reads of the wild type gene of KlenTaq, separated by reading sense (forward or reverse strand). **A** Distribution of the Qscore separated by forward and reverse strand. **B** Comparison of the error rate per position in forward (horizontal axis) vs reverse (vertical axis) strands. **C** Comparison of the fits on the reverse and forward strands for some chosen position-nucleotide.
