# Supplementary material for: Accurate gene consensus at low nanopore coverage
Source: Gigascience. 2022 Nov 9;11:giac102. doi: 10.1093/gigascience/giac102 (PMC9646519; doi:10.1093/gigascience/giac102)
Supplement: giac102_Response_to_Reviewer_Comments_Original_Submission [file giac102_response_to_reviewer_comments_original_submission.pdf]

# Author's Response To Reviewer Comments

Close

Dear Dr Zauner,

I submit a corrected version of our manuscript, in which we have incorporated all the suggestions done by the reviewers. In particular, as requested, we have extended the explanation of the method, clarified the accessibility to the raw data and included the full code to reproduce the analysis in our git hub. Below, we address one by one the points raised by the reviewers.

Best wishes,
Yannick

We thank the reviewers for taking their time to carefully read our manuscript and provide valuable feedback. They have raised a number of interesting questions that we address below and that have been included in the reviewed version of our manuscript.

#-----------#

Reviewer #1: The paper "Accurate gene consensus at low nanopore coverage" describes a method SINGLe, which characterizes the position-specific systematic sequencing error pattern with logistic regression, and produces higher quality consensus calls with low coverage over a small region of highly covered area. SINGLe focuses on mutation calling using nanopore sequencing in a small region of interest on a genome. This type of tool is novel and can be useful for development for e.g. clinical tests or any other amplicon sequencing application. The coverage required to converge at a mutation call is smaller than other tools previously reported, which saves sequencing cost and/or gives more reliable results. Previous tools focus on genome assembly, while SINGle focused on mutation calling in small regions of interest on a genome.

Major feedback:
1 - The manuscript only demonstrates that the method works on one specific gene region. The authors should at least include an extra gene region to showcase that the method is robust regardless of the composition of the bases and motifs in that target region. At the moment, it is unclear if it is applicable to other regions as the authors suggested.

For this short manuscript, we limited the discussion to the KlenTaq gene used as an example This is to avoid overloading the manuscript and keep it at a reasonable length. However, we have used SINGLe in the NBI nicking enzyme gene (33% GC content) in our laboratory and the results are similar. In fact, since SINGLe applies a probabilistic approach on the erroneous nanopore reads, irrespective of what mechanism actually causes the systematic errors, we do not expect a strong dependence on gene properties. We have included this point in the discussion: “SINGLe needs to characterize the sequencing errors done on an appropriate reference sequence. Because it applies a probabilistic approach on the erroneous nanopore reads, irrespective of what mechanism actually causes the systematic errors, it should not depend on the genes’ properties. “

2 - The method could be more clearly described. For instance, there is mention of reweighting based on priors, but this is not further explained. The method is not very complex, but nevertheless it would be good to be explicit about these things.

We thank the reviewer for this suggestion and have extended the explanation of the method: “To adapt the classifier, an a priori expectation of mutations ($p_{prior-right}$) is needed, which must come from independent information. In the presented case, the variant sequences originated from an epPCR, for which we possessed an estimate of the mutation rate $m_{n \to n'}$ given by the manufacturer [14]. We computed the a priori probability of observing nucleotide n’ as $p_{prior-right}(n \to n') = m_{n \to n'} \cdot /[\Sigma n$

counts_reference(n) Σn′ m_n→n′ ], where is the mean number of mutations expected in the library (reported by the epPCR kit manufacturer), and counts_reference(n) is how many times the nucleotide n is present in the wild type DNA strand. We also obtained the a priori expectation of an observed mismatch to be a sequencing error (p^{p,n}_prior−error) as the sequencing error rate at that position in the wild type set, independently of the Qscore. Using these values we computed for each strand, position (p) and nucleotide (n), the probability of observing a mutation with Qscore=Q as P^{p,n}_mutation (Q)= [counts^{p,n}(Q) /ΣQ′ counts^{p,n}(Q′) ]·p^{p,n}_prior−error where counts^{p,n} is the number of times the nucleotide n appears in position n across all the reads. Similarly, we computed the probability of observing a wild type nucleotide with Qscore=Q as P^{p,n}_wildtype(Q)=[counts^{p,wildtype}(Q)/ ΣQ′ counts^{p,wildtype}(Q′)] · p_prior−right(wildtype→n). Finally, we normalised N^{p,n}_correct (Q)= P^{p,n}_wildtype/[ P^{p,n}_wildtype(Q)+ P^{p,n}_mutation(Q)]. This process shifts the logistic regression towards the higher Qscore, allowing the classifier to accept a number of observed mismatches consistent with the prior expectation (Figure 1D). The fits were done independently for each position and possible mismatched nucleotides."

Moreover, the depth of the number of wildtype sequences needed for training is not clearly stated in the main text. It is crucial to know if other people want to apply the method to larger regions in the genome. (The author mentioned they used 5847 full length coverage of the wildtype but no discussion/ analysis was provided on whether this can be lower or what the lower limit is.)

We have added a discussion on this topic on the Supplementary Material (page S12), and refer to it in the main text: " Please refer to the supplementary material for a brief discussion on 'How many Nanopore reads are needed to fit SINGLe?'."

3 - While the regressions are done strand specific, no analysis is done on e.g. strand bias, while it is known to be very important for accurate consensus calling. Are the results indeed different for the different strands?

Indeed, the patterns of errors in each position are different on the forward and reverse reads. We added a discussion on the supplementary material (page S13), and referred to it in the main text: "We also separated the fits for forward and reverse strands as the error rate per position is different in each case (see 'Are reverse and forward reads different in Nanopore Sequencing' in Supplementary material). ". It is true that a better consensus is obtained by combining nanopore reads from both directions, but this is independent of SINGLe.

4 - Have the authors considered taking into account the observed neighbour correlation (Fig S2) in the prediction (i.e. by taking more than 1 feature into account in the logistic regression).

(Note that Fig S2 is now Fig S3) This is an interesting suggestion. This is one option that we have considered at the beginning of the project and that would be interesting to consider as an extension of our approach for higher mutational loads. In the current approach we assume that true mutations are scarce, and therefore that the neighbor pattern is always the wild type. It is under this assumption that we build one independent logistic regression for every position and every possible mismatch.

5 - It would make the work more valuable if more discussion around the potential applications of this method is provided. It is mentioned in the introduction Line: 72 that it can be used in an evolution experiment. The case demonstrated is ePCR introduced mutations on a single gene (KlenTaq Polymerase gene). Can the authors clarify the link between the two, or link to other clinical applications such as tumor gene sequencing?

Thanks for the suggestion. We have clarified that error prone PCR is the method used for generating gene libraries in directed evolution experiments: "For example, in directed evolution experiments the genetic libraries typically originate from a single ancestral sequence (the wild type) that has been submitted to limited randomization, for example using error-prone PCR (epPCR) [2]. " We have also included as potential applications structural variants detection in cancer, with a reference: "Another example is the detection of structural variants in cancer cells [3]." in the introduction.

Minor feedback:
6 - Some figures can be enlarged. (e.g. Fig 3B).

We have increased the size and quality of our figures. In particular, we have changed the presentation of data in Fig. 3B to make it easier to interpret.

7 - The manuscript is submitted as a technical note type of paper. According to the reporting standards, the manuscript should contain a section on data availability and testing to enable readers to easily reproduce the results. However, the access to data is not clearly described in the main text. Testing procedures are also not provided in github as far as this reviewer can deduce.

Previously, this information was included in the availability section, but now it has been moved to "data availability" following GigaScience guidelines and the reviewer's suggestion. We have uploaded an R notebook including all the codes used for this study to our git-hub repository.

8 - Typo:Fig 3B xlabel: diference

We have corrected the typo.

9 - Typo: Line 266: "f"igure should be capital

We have replaced 'figure' by 'Figure'.

10 - Clarify Availability, Operating system (If it is a R package, I expected it to be cross platform. Or please report precise operation system (e.g. x86_64-pc-linux-gnu (64-bit)), Programming language: R (version x.x.x), Other requirements (missing). The instructions to use the program are minimal on github. Consider copying the vignettes to Github README:
https://github.com/rocioespci/single/blob/master/vignettes/single.Rmd

We have clarified the information on the availability requested by the reviewer and included the vignette to the git-hub readme file. SINGLe is now available in bioconductor, a repository for R packages related to biological assays. Therefore, it has been tested in different platforms (linux, windows, macOS), and this information has been included in the manuscript (availability section). The Github readme has been extended, including the information on the vignette, as suggested by the reviewer.

#-----------#

Reviewer #2: In the manuscript submitted by Rocio et al. the authors present a new method, SINGLe, to reduce noise in nanopore sequencing data in the scope of direct evolution experiments. In essence, SINGLe is a machine learning approach utilizing logistic regression trained on reference sequences to model the probability of true variants in random mutagenesis libraries (by error-prone PCR) sequenced using Oxford nanopore technology. In the results, the authors show that SINGLe shows a better performance than other approaches (like nanopolish or racon/medaka) with respect to the discrimination between correctly and incorrectly called SNPs and the number of reads necessary for building a correct consensus sequence of one variant. In summary, the submitted manuscript is a technical note that is relatively long but incomplete in terms of details on methodology and execution. Consequently, I have several major issues that will be listed in the following.

1) The structure of the manuscript could be enhanced from my point of perspective in several ways. First, the authors are not clearly separating descriptive methodology from the presented results. For instance, details about the applied library preparation, sequencing, and analysis protocols and pipelines are somewhat distributed over several sections, making it hard to follow the authors without hopping through the manuscript.
Also, the manuscript has in essence just two main sections, i.e., Findings and Methods. However, the Findings section contains also a Method subsection but at the same time also contains a Background subsection, which is clearly not a finding.

This article is submitted as a technical note and it follows GigaScience editorial guidelines. The Findings section includes the background, the methodology related to SINGLe (the new methods we are presenting in this work), and the results. On the other hand, the Methods section includes previously available methodologies that were used. To clarify this, we have changed the title to 'SINGLe method' inside the Findings section.

2) The writing could be enhanced. There are quite a lot of occasions of grammar errors or misspellings.

We have thoroughly reviewed the grammar and the spelling of the manuscript.

3; line 81 ff.) It is not clear what the data basis for the following observations are. Even though the overall statement of the correlation of quality scores and errors and the fact that nanopore errors are not evenly distributed is not to be questioned, from a strict scientific point of view it should be made clear how this was observed, precisely on what data.

We specified the sample we used for this analysis "We used a nanopore sequencer to read 5847 strands of the wild type KlenTaq gene (length 1662 nucleotides), for which we have a ground truth sequence obtained by Sanger sequencing (supplementary Sequence S1). In this data set, we can confidently attribute mismatches between the read sequences and the known wild type as sequencing errors, and matches as correct reads."

4; line 82 f. and Figure 1A) As pointed out in the manuscript, the q-scores are directly related to the error rate, i.e. a low q-score indicates an erroneous base call. However, (the reference to) Figure 1A does not make this so obvious as it sounds in the text, as the figure is not normalised with respect to total counts. In fact, the total numbers of correct/false base calls is irrelevant to make this point, it is more about the reported error rate (the q-scores) vs the observed error rate (also in terms of q-scores). Something like a qq-plot would be more appropriate to make this point.

The first affirmation is only partially true. While it is true that errors tend to have a low Qscore, there are also plenty of correct reads that have a low Qscore, as shown by the lower peak of the green distribution on figure 1A. Thus, a low q-score does not necessarily indicate an erroneous base call. The aim of figure 1A is to make this point clear. So we have clarified: "The Qscore assigned to each nucleotide tends to be low when a wrong nucleotide is assigned, as expected. Notice that the inverse is not true: some low Qscores correspond to correctly basecalled bases, so both distributions overlap. Thus, a simple classification based on the Qscores is not possible to distinguish signal from noise. "
We chose not to show the normalized distributions in the main text because the peak on the errors distribution is higher than the correct reads. To the distracted reader, it could look that nanopore sequencers make a higher proportion of errors than correct reads and this is not true. We have included the normalized distribution in the supplementary material (Figure S1).

5; line 96 ff. and line 110) Where were the 6000 KlenTaq reference sequences obtained from? The later Methods section only details about the small and large variant library, not about the wild type library.

We agree that the information was not properly stated. The reference KlenTaq was measured together with the seven clones. The overall experimental procedure is similar, and it has now been specified in the Methods.

Is there any kind of PCR involved here, and if so, how do the authors separate between sequencing errors or other errors sources (because, for instance, an error introduced during PCR when amplifying a specific gene is - if sequenced correctly - not a sequencing error even if the generated read mismatches with the reference)?

This is indeed true: if there was a base substitution during PCR a mismatch with the reference would not mean a sequencing error. To reduce the possibility of mutations during PCR, we used Q5 high fidelity polymerase (NEB) for amplification of the gene prior to nanopore sequencing, following the suggested conditions. This method has an extremely low error rate during replication: 10-7 substitutions per base per doubling [Potapov V, Ong JL (2017) https://doi.org/10.1371/journal.pone.0169774], which is 6

order of magnitudes lower than the Nanopore sequencing error rate. Taking into account these considerations, we neglected the substitution occuring during the PCR amplification. In addition one may note that if the library to be SINGLed is prepared following the same library preparation protocol as the reference (for example, as a pool), then the error introduced at the molecular level, if systematic, would be included in the SINGLe correction.

6; line 105 and ff.) The abbreviation ePCR for error-prone PCR is very unusual. To the best of my knowledge, epPCR is the established short term for error-prone PCR and I would advise in using it instead of ePCR.

We replaced "ePCR" by "epPCR" in the manuscript.

7; line 106 ff.) The two sentences beginning with 'In this range …' are difficult to read and should be revised. Also, the sequencer does not report a mismatch nor a wild type or a mutation, it just reports a nucleotide that can either be correct or not in comparison to a specified reference.

The text has been modified and now it reads: "For nucleotides read as wild type their Qscore are directly transformed into a $p_{right}$ according to the Q values reported by Guppy: $p_{Guppy}=1-10-Qscore/10$.". We have also removed the expression "the sequencer reports a mismatch/wildtype".

8; line 123 ff.) What type of logistic regression was used, what is the underlying distribution? How was the model evaluated, for goodness of fit (over- underfit)?

We used a binomial model for the logistic regression. We have included this information in the main text: "We fitted this relation by a logistic regression using a binomial model". We have also added in the Supplementary material an evaluation of the goodness of fit calculated as 1- deviance of null model / deviance of model and discussed the implications on SINGLe fits (page S12).

9; line 130 ff.) How well does the 'estimation of the mutation frequency' of the ePCR that the authors possess reflect the actual mutation rate (see also later comments)? Is there other literature on that issue?

We verified the mean number of mutations in the consensus sequences obtained after analysis of the large library: 8.4 mutations / kb. This value is close to the estimated value by the Mutenzyme II kit (8 mutations/kb), the commercial kit we used for error prone PCR.

10; line 131 ff.) How is the a priori sequencing error rate defined and what is it based on? And how was the re-weighting of the wild type fit carried out in detail?

We have extended the description of the method on the main text to clarify these points: "To adapt the classifier, an a priori expectation of mutations ($p_{prior-right}$ ) is needed, which must come from independent information. In the presented case, the variant sequences originated from an epPCR, for which we possessed an estimate of the mutation rate $m_{n\rightarrow n'}$ given by the manufacturer [14]. We computed the a priori probability of observing nucleotide n' as $p_{prior-right}(n\rightarrow n')= m_{n\rightarrow n'} \cdot /[\Sigma_n counts\_reference(n) \Sigma_{n'} m_{n\rightarrow n'} ]$, where is the mean number of mutations expected in the library (reported by the epPCR kit manufacturer), and counts_reference(n) is how many times the nucleotide n is present in the wild type DNA strand. We also obtained the a priori expectation of an observed mismatch to be a sequencing error ($p^{p,n}_{prior-error}$) as the sequencing error rate at that position in the wild type set, independently of the Qscore. Using these values we computed for each strand, position (p) and nucleotide (n), the probability of observing a mutation with Qscore=Q as $P^{p,n}_{mutation} (Q)= [counts^{p,n}(Q) /\Sigma_{Q'} counts^{p,n}(Q') ]\cdot p^{p,n}_{prior-error}$ where $counts^{p,n}$ is the number of times the nucleotide n appears in position n across all the reads. Similarly, we computed the probability of observing a wild type nucleotide with Qscore=Q as $P^{p,n}_{wildtype}(Q)=[counts^{p,wildtype}(Q)/ \Sigma_{Q'} counts^{p,wildtype}(Q')] \cdot p_{prior-right}(wildtype\rightarrow n)$. Finally, we normalised $N^{p,n}_{correct} (Q)= P^{p,n}_{wildtype}/[ P^{p,n}_{wildtype}(Q)+ P^{p,n}_{mutation}(Q)]$. This process shifts the logistic regression towards the

higher Qscore, allowing the classifier to accept a number of observed mismatches consistent with the prior expectation (Figure 1D). The fits were done independently for each position and possible mismatched nucleotides. "

11; line 137 ff.) In this paragraph, the authors explain how they have inferred q-scores for nucleotide positions that were deleted in the mutant type read set. First of all, what does 'fixed to the minimum Qscores of their nearest neighbors' mean precisely? To the minimum of the direct two 5' and 3' neighboring nucleotides or over a window?

To the minimum of the direct two 5' and 3' neighboring nucleotides, not over a window. We have clarified in the main text: "we fixed their confidence value as the minimum of the Qscore of their direct nearest neighbors in the nucleotide sequence."

Second, while there is indeed an obvious correlation of q-scores of neighboring nucleotides in a sequencing read, in my opinion this is for this application an oversimplification, as it is biased towards correctly called bases (which clearly make up the majority). As the authors have already pointed out, erroneous base calls tend to have lower quality scores and they do occur all over the read. Then, how well do q-scores of these erroneous positions correlate with their neighbors? A deletion event introduced during sequencing is also an error and should be in line with or within range of q-scores of erroneous base calls.

We have included panel C in figure S3 (previously Fig S2) to address this question. We plotted the distribution of difference in the Qscore (reported by guppy basecaller) for a nucleotide and its closest neighbor on the 3' side, classified by being an error or a correct read. In both cases, most nucleotides have a small difference of Qscore with its neighbor, and the difference is smaller for errors. As a control, we showed this is not the case for random pairs of nucleotides. This supports that for any read, and in particular for errors, there is some correlation between the Qscore of one nucleotide and its neighbors.

And why were insertions ignored altogether?

It is mainly a methodological constraint. Insertions can be very diverse (they do not have a defined length), and it is hard to accumulate enough data to fit them. For each position and possible insertion, we would need a few appearances by error in the wild type sequence to fit them. While the current version of SINGLe ignores them all together, the corrected confidence values for no-insertion positions and the original Qscores assigned to the insertions could be used in other methods to compute the consensus (for example, samtools) and decide whether a insertion is true or is a sequencing error. Finally, we conceived SINGLe for gene libraries using error prone PCR. In these cases, insertions are rare events (less than 1% of the mutations according to the kit's manufacturer).

We have added to the text: " Insertions were ignored as very few are expected (<1%) and it is not possible to obtain enough reads to fit all insertion possibilities. In applications where SINGLe is used to compute a high quality consensus, the original score of the inserted bases can be carried over. "

12; line 173) How many variants were sequenced exactly? This should be known as each library was tagged with an individual barcode.

We have included this information in the section Samples preparation and sequencing: "After data pre-processing (see section minION reads pre-processing), we had the following reads per variant: 716 (variant 1), 453 (variant 2), 645 (variant 3), 428 (variant 4), 422 (variant 5), 369 (variant 6), 358 (variant 7)."

13; line 189 ff.) If the threshold is zero, i.e. the cutoff on p_right and p_guppy is zero, signal is defined as mismatches of know mutations, no matter the of actual q-score or p_right, and noise as mismatches of non-mutated positions. The actual nucleotide sequence is equal for both cases. How can it be that the signal to noise ratio is different for guppy and SINGLe?

The difference arises in the method for counting. The weighted count of true positives (signal) is not the number of correct nucleotides, but the sum of the p_right/p_guppy for those nucleotides. Because SINGLe reduces the p_right for the errors (false positives) in comparison to the scores assigned by Guppy, the ratio signal to noise is higher, even when a cut-off 0 is used.

In figure S5 (previously fig S3), in which the counts are not weighted, the ratio signal to noise for SINGLe and Guppy matches at cut off zero, as the reviewer points out.

We have updated the paragraph to clarify this point: "Notice that the ratio is different for both methods

at cutoff zero because the counts are weighted by p_right."

14; line 191 ff.) How were the counts weighted by p_right exactly. In this regard, the signal to noise ratio seems to be equal despite weighting being applied or not (comparison of Fig2 and supplementary Fig3), so what is the actual methodological difference here?

(Note that Fig S3 is now Fig S5).
This observation was answered in the previous comment and we have also clarified how the counts are weighted by p_right in the main text: "The counts are weighted by p_right for each nucleotide, i.e. instead of summing one for each occurrence, we summed the p_right associated with the nucleotide." While the general shape of the figure is similar, as pointed out by the reviewer in the previous comment (#13), when using the weighted values both methods differ, while this is not true for the regular counts. We decided to show Figure 2 with weighted counts on the main text because it shows how the weights increase the scores (i.e. the p_right values) of the correct reads and reduce those of the incorrect reads.

15; line 194 ff.) This paragraph is somewhat imprecise and lacks scientific references on how consensus calling and polishing of nanopore reads is done on a broad scale. For instance, nanopolish uses the raw signals of the nanopore sequencer (fast5) in combination with HMMs to detect base variations, which are then filtered and eventually combined.

We modified the text to explain how consensus is done by other methods, including references: "We also computed the consensus using Nanopolish, which works directly on the raw electrical signal instead of the basecalled sequences in combination with HMMs to detect base variations [7], Medaka that counts nucleotides and uses a neural network to compare to a draft assembly and define mutations [9], and NextPolish which takes into account the neighbours of the nucleotide [15]."

16; line 197 ff.) As far as I understood SINGLe, it essentially transforms the q-scores into a probability score. But how is that now incorporated into a consensus calling process? All the methodological details on this are missing in the manuscript. This also applies to the p_guppy and 'no weights' consensus pipeline. And what is the difference between p_guppy and 'no weights'?

The reviewer has correctly understood that SINGLe transforms the q-scores to a probability score. These probabilities are then used to weight the counts of occurrence of nucleotides used in consensus calling. We have clarified this point: "We compared the consensus sequences of the 7 KlenTaq variants obtained by SINGLe and by other methods. Given several aligned nanopore reads of the same variant, the consensus can be computed by simple majority counting how many times each nucleotide was read in one position and keeping the most frequent. We call this method 'no weights'. The counts can also be weighted by p_Guppy and choose the nucleotide with larger weighted count. We call this method 'Guppy'. Or the counts can be weighted by p_SINGLe ('SINGLe' method). "

17; line 203 ff.) The consensus calling was done for each of the seven variants independently, so seven times 47 times 50 sets. I would recommend to be precise here in the manuscript.

We have clarified: "We computed the consensus independently for each variant using subsets of 3 to 50 sequences drawn randomly from all available reads, and repeated 50 times for each subset size.".

18; line 208 ff. and Fig 2B) Why did nanopolish failed on the #1 dataset? Given the results on the other variants this seems to be highly unlikely.

Variant #1 has two mutations: G23A and C245T. Nanopolish fails systematically to find G23A.

19; line 223 ff. And Fig 2D) Based on the figure it can not be concluded that nanopolish misses mutations instead of reporting false positives, as false negatives are not considered or measured in this manuscript. This even more highlights that sensitivity (recall) is an important aspect, especially in the field of variant detection. So what is the false negative rate or the sensitivity? The whole evaluation is more about precision, but precision often comes at the price of sensitivity and finding low abundant / rare variants is of high interest for many applications. Why not defining TP=mismatches of known

mutations above p_right, FP=incorrect mismatches above p_right, TN=incorrect mismatches below p_right, and FN=mismatches of known mutation below p_right and presenting also incorporating evaluation statistics like F1 scores?

Following the reviewer's suggestion, we updated Figure S6 (previously, Fig S4) to show not only all detected mutations but also TP (true mutations), FP (false mutations), TN (true wild types) and FN (false wild type) nucleotides in the consensus obtained from subsets of nanopore reads, plus the sensitivity and the specificity.
We have also updated this paragraph in the main text to make it more accurate: "Figure 2C shows the total number of mismatches (averaged over the 50 trials) reported by each method for variant #3, according to the number of reads used to compute the consensus. For any set size, SINGLe reports the closest number of true mutations compared to the other methods. In Figure S6 this is analyzed in more detail: we classified the nucleotides in each consensus sequence according to true/false mutations and true/false wild types and observed that actually SINGLe detects true mutations with the fewest number of reads while keeping the lowest rate of false mutations. Nanopolish has a high rate of false wild types and underestimates the true mutations. Medaka has a similar behavior and on top adds false mutations. Consensus using Guppy scores or no weight report a high number of false mutations."

20; paragraph line 242) The way the authors have compared the results of the large library with that of the seven known variants is, at its best, only partially confirming the results of the latter one. First, why was medaka favoured over nanopolish if nanopolish showed clearly the better performance on the seven variants dataset?

We compared our method against Medaka in the main text because it is the method suggested by Oxford Nanopore and the most widely used. Nevertheless, comparison to other methods, including Nanopolish, are also reported in the supplementary material.

Given the related results in the supplement, nanopolish failed this time on the majority of the shown datasets. Why? What are these variants and how many actual mutations do they have, is there a pattern?

The reviewer is right and actually we found a mistake on the parsing of the mutations output by Nanopolish. We have now corrected (and double checked) this and the results have improved. Nevertheless, Nanopolish has a poor performance in some examples. The sequences of these variants are listed in table S2. In most cases, Nanopolish makes one or two mistakes in the sequence, but we do not find any pattern that relates them. Here we enumerate the errors:
For barcode AAATTACGAAACTTAGGTGGAAATGCAGATATTAG, Nanopolish predicts two extra substitutions (A1548G and G633A) in the consensus done by subsets of reads compared to consensus computed with all available sequences.
For barcode ACGCGCACGTAGACAGATACGAAGGCCGACAGGAG, predicts an extra substitution (C592T) in the consensus done by subsets of reads compared to consensus computed with all available sequences.
For barcode GAGTTAAGGTGCAAGGGTATATGTTCCCATGTG the deletion A1152- is missed in the consensus done by subsets of reads compared to consensus computed with all available sequences.
For barcode GTCCATGTAAGCACAGGTCCTACTTAAGATGTTAG, it predicts an extra substitution A957G in the consensus done by subsets of reads compared to consensus computed with all available sequences.

Second and above all, the way the ground truth is calculated is unclear. Was this done for each of the barcodes sets or over all reads?

The consensus was computed for the reads associated to each barcode independently. We clarified this point in the main text: "We sequenced the library using nanopore, grouped the reads according to the barcode and computed the consensus sequence for the reads associated to each barcode.".
If the former was the case, how does it comes that raw reads have an inferior performance (see supplemental Figure S6).
In figure S6 (now S7) 'raw' was accounting for the consensus computed with 'no weights'. We have corrected the legend of this figure. The worse performance is consistent with the observations of the first 7 mutants analyzed in the previous section.
If the latter was the case, a consensus over all of the 1200 variants would most probably be skewed towards the most present variants or towards the wild type, given the fact that epPCR should introduce errors evenly and at a random rate. In this sense, this read based consensus ground truth is biased towards high precision but low recall if the consensus ground truth harbors less mutations than the

actual ground truth, as this would result in not calling a mutation (high precision but low recall) being better than calling a wrong mutation (low precision but high recall).
This is correct, that is why we computed the consensus for the reads associated to each barcode independently.
Finally, in this case SINGLe does not converge after 5 reads but after 10 reads, as there is a significant drop right after 5 reads.
We have re-written this sentence in the main text: "As shown in Figure 3A, SINGLe returns the correct consensus sequence over 90% of the times when using at least 5 reads, while Medaka needs 15."

21; line 253 ff. and Fig 3B) This is hard to read and understand, as the figure shows no percentages but they are discussed likewise in the manuscript. Also, the figure legend is missing essential information (e.g. what the color coding exactly means).

We have edited Figure 3B, and the text: "We computed consensus sequences using SINGLe or Medaka for all the mutants in our library, provided that the identifying barcode is present at least four times in our dataset, and compared the number of mutations reported by both methods (Figure 3B). Out of the 1174 variants for which we had at least 10 reads, both methods report the same consensus in 1039 cases (89%). Among the 610 variants for which we had 6 to 10 reads, we obtained the same consensus sequence using Medaka or SINGLe only in 339 variants (56%), and for the 839 variants with less than 5 reads only 23% of the variants obtained the same consensus with both methods. For the other variants, independently of the number of available reads, Medaka returns more mutations than SINGLe in the wide majority of cases. This is consistent with the observation in the previous section: Medaka tends to predict more mutations than there actually are. Less than 1% of all computed consensuses have more mutations by SINGLe than by Medaka."

22; line 264) I tend to disagree, but SINGLe does not correct systematic errors. It may reduce systematic errors, but it does not, as far as it is described, specifically correct (newly) known systematic errors, as otherwise the type and nature of the systematic error along with the appropriate measurements would have been presented.

We replaced this line by: "If SINGLe improves the consensus sequences by reducing systematic errors from the basecalling, then the mutations predicted for a randomly mutated gene library should be homogeneously distributed along the sequence. "

23; line 266 ff.) What is the exact data basis here? How many datasets are below five, between 6 and ten, and above ten read counts, and how are they distributed?

There are 1174 variants with more than 10 reads, 610 with 6 to 10 reads, and 839 with less than five reads. We have incorporated this information, as cited in the response of comment #21.

24; line 311 ff.) SINGLe needs optimally the true reference sequence and the expected mutation rate, which both could also be obtained by other means, e.g. using high accuracy short read sequencing, as the authors have pointed out. However, this is in contradiction to the highlighted advantages of this approach presented here, i.e., low laboratory effort at high sequencing throughput. Have such applications being conceived or carried out to give a better understanding of the practicability of using SINGLe for such cases?

One example in which SINGLe can be helpful is directed evolution experiments. Generally, these start from a known wild type DNA sequence coding for a protein whose activity is to be enhanced. This wild type sequence is obtained by Sanger sequencing and it is used for the general experimental design, so it is not an extra constraint added by SINGLe. To proceed, copies of this gene are made by PCR adding random mutations on the gene. Today, commercial kits are available and the mean number of mutations on each variant is provided (and adjusted by the experimental conditions). Finally, SINGLe requires several reads of the wild type gene to characterize the error rate. This is the unique experimental effort that SINGLe implies. Because it is a unique wildtype gene, enough DNA material can be obtained by PCR and purification, and added to the sequencing run using Oxford Nanopore's pipeline for multiplexing of samples. In return, as SINGLe lowers the number of duplicated reads for each variant from ~20 to ~5, the number of variants in a single sequencing run is multiplied by four, allowing to study larger gene

libraries.

25) If I understand the methods correct, the same epPCR protocol was applied to the small and large library, but with a different cycle number (25 vs. 30), resulting in different mutation rates (3 muts/kb for the small and 8muts/kb for the large library). Why was a different rate chosen to begin with?

There is no particular reason for the different mutation rate. They are two independent experiments. Nevertheless, the high mutation rate for the large library (given by more cycles of PCR in error prone conditions) reduces the possibility of having wild type genes in the library for the downstream protocol. This is convenient as we aimed to find mutations in the gene. On the other hand, that is not a problem for the small library because the clones are individually chosen and their (non-wild type) sequence confirmed by Sanger sequencing.

Supplemental Table S2 shows the actual variants of the small library. Here, the first variant only shows two substitutions, which corresponds to a rate of 1.2muts/kb, whereas on the high end the variants show 4.7muts/kb. Is this the expected deviation (here about 60%), and if so, does this also apply to the large library, and if so, how well is this captured in the different datasets of the large library?

The consensus sequences obtained with SINGLe in the large library, for variants with more than 5 reads, contain between 1 and 30 mutations (thus 0.6 to 18 mutations/kb). This deviation is in line with previous studies on the mutational profile of error prone PCR libraries (see for example Drummond, D. A., Iverson, B. L., Georgiou, G., & Arnold, F. H. (2005). Why high-error-rate random mutagenesis libraries are enriched in functional and improved proteins. Journal of molecular biology, 350(4), 806-816. doi:10.1016/j.jmb.2005.05.023).
How well do the different tools perform for the different types of variants (in terms of number of mutations but also substitutions versus indels)?
We compared the percentage of each type of mutation detected in the consensus sequences vs the values reported by the manufacturer’s kit. We splitted the analyses for the variants according to the number of available reads (up to 5 reads, 6 to 10, more than 10), and by the method used to compute the consensus. We plotted the results in Fig S9, and computed the correlation between the observed values and the reported ones.
For all methods except Nanopolish, the correlation is the highest (.94) when looking at the variants with more than 10 reads. But when lowering the number of available reads, SINGLe outperforms the other methods.

This is important, as epPCR also have shown to be biased to some extent, favoring specific mutations over others, so introducing systematic errors of its own (see, for instance, Vanhercke et. al 2005).

Yes, it is well known that error prone PCR mutations are biased and these biases are indicated in the manual of the kit used ( available in
https://www.agilent.com/cs/library/usermanuals/Public/200550.pdf).

26) What was the decision of the authors to only include nanopolish racon/medaka for their evaluation? These tools have originally been developed to correct genome assemblies based on signal or read information, with nanopolish being considered as almost outdated by now. Other methods have been proposed to specifically correct for systematic errors in nanopore data (e.g. Homopolish, Nextpolish, NanoReviser). Why those newer tools have not been included for the evaluation?

As we discuss in the main text, most tools for nanopore sequencing are intended for de novo genome assembly rather than detecting few mutations in a well known sequence. Originally we had included Medaka because currently it is the standard for detecting mutations in nanopore sequencing reads and it is the software suggested by Oxford Nanopore, the company that provides the sequencer. We had also included Nanopolish which is less used currently because it takes a longer computation time, but it has been reported to be a little more accurate than Medaka (see for example Lee, Jin Young, et al. "Comparative evaluation of Nanopore polishing tools for microbial genome assembly and polishing strategies for downstream analysis." Scientific Reports 11.1 (2021): 1-11.).
Homopolish is designed for polishing genomes, a different use of nanopore sequencing that the one we propose in this article. Homopolish makes use of the available homologs sequences for correcting the reads, forcing natural occurring sequences. This is not the aim for reading DNA strands with synthetic mutations added in the lab.
NanoReviser is a method to improve basecalling using the raw electrical signal plus the predictions

already done by other basecallers. This software depends on Albacore, an old basecaller from Oxford Nanopore which is not accessible anymore and that has been replaced by Guppy. We could not install NanoReviser to compare.
We have included in our manuscript NextPolish among the methods tested using KlenTaq's seven variants. Results are included in figures 2 and S6, and in the main text: "We also computed the consensus using (...) NextPolish which takes into account the neighbors of the nucleotide [15]." Also: "Consensus using Guppy scores or no weight report a high number of false mutations, and so does NextPolish (though with a lower error rate)." In the Discussion: "This implies a faster convergence than other methods currently used: nanopolish requires 8 to 15 reads, Medaka needs at least 20-50 reads and NextPolish between 15 and 50 reads to achieve a similar performance. " In Methods: "Consensus by NextPolish. We used nextPolish (version 1.4.1) with options task=best, rerun=3, genome= KlenTaq wild type sequence, and lgs_options -min_read_len 1k -max_depth 100."

Figures:
27; Figure1B) It should be made clear that only a specific window of the whole gene was selected here (in contrast to the supplement).

We have clarified in the legend of figure 1 "Only positions 100 to 150 are shown here. An equivalent plot for all positions is available in Figure S2."

28; Figure1C/D) The legend states that a black dot is the proportion of correct reads against the q-scores. A sequencing read does have as many q-scores as it has nucleotide positions. How was this averaged or is a black dot actually the proportion over a single base position?

It is the proportion over a single base position. We have clarified it in the text: "Black dots are the proportion of correct nucleotides with a given Qscore in one position and in comparison to one possible error."

29; Figure 2 and 3) The legends are too long. A legend should not contain any interpretation of the shown data but only all information necessary to fully understand the figure.

We have reduced the length of the legends.

30; Supplementary Table S1) This is not a table.

We changed the name "Table S1" to "Sequence S1".

31; all Figures) I would suggest to provide higher resolution graphics. Overall, the depicted information is hard to grasp visually. I would rather separate some of the sub-graphics into individual figures but make them larger in size instead.

We have increased the figures resolution.

Close
