# Supplementary material for: Accurate gene consensus at low nanopore coverage
Source: Gigascience. 2022 Nov 9;11:giac102. doi: 10.1093/gigascience/giac102 (PMC9646519; doi:10.1093/gigascience/giac102)
Supplement: giac102_Response_to_Reviewer_Comments_Revision_1 [file giac102_response_to_reviewer_comments_revision_1.pdf]

Author's Response To Reviewer Comments

Close

Dear Dr Zauner,

We submit the manuscript with the requested modifications:

1. Our dataset in GigaDB has been added to the bibliography and referred to in the Data availability section:
"The data set supporting the results of this article are available in the European Nucleotide Archive repository, ERP135743, run ERR8778685 (nanopore reads of seven mutants and wild type) and ERR8778797 (nanopore reads of library), and in GigaDB [18] (Sanger sequencing for the seven mutants of Klen Taq, sequence of wild type KlenTaq gene, and the full code and processed data to reproduce the figures in the manuscript and supplementary material)."

2 and 3. SINGLe has been registered in Scicrunch and bio.tools and cited in the availability section:
"Software is registered in scicrunch.org (identifier SINGLe, RRID:SCR_022810) and biotools (https://bio.tools/SINGLe)."
Please notice that in scicrunch the curation takes around 5 business days so the link should be available by the end of this week or beginning of next one.

4. We have checked that our manuscript follows the journal style.

Sincerely,

Close
