# Supplementary material for: Accurate gene consensus at low nanopore coverage
Source: Gigascience. 2022 Nov 9;11:giac102. doi: 10.1093/gigascience/giac102 (PMC9646519; doi:10.1093/gigascience/giac102)
Supplement: giac102_Reviewer_1_Report_Original_Submission — Jeroen de Ridder -- 5/9/2022 Reviewed [file giac102_reviewer_1_report_original_submission.pdf]

Reviewer Report

Title: Accurate gene consensus at low nanopore coverage

Version: Original Submission **Date:** 5/9/2022

Reviewer name: Jeroen de Ridder

Reviewer Comments to Author:

The paper "Accurate gene consensus at low nanopore coverage" describes a method SINGLe, which characterizes the position-specific systematic sequencing error pattern with logistic regression, and produces higher quality consensus calls with low coverage over a small region of highly covered area. SINGLe focuses on mutation calling using nanopore sequencing in a small region of interest on a genome. This type of tool is novel and can be useful for development for e.g. clinical tests or any other amplicon sequencing application. The coverage required to converge at a mutation call is smaller than other tools previously reported, which saves sequencing cost and/or gives more reliable results. Previous tools focus on genome assembly, while SINGle focused on mutation calling in small regions of interest on a genome.

Major feedback:

- The manuscript only demonstrates that the method works on one specific gene region. The authors should at least include an extra gene region to showcase that the method is robust regardless of the composition of the bases and motifs in that target region. At the moment, it is unclear if it is applicable to other regions as the authors suggested.
- The method could be more clearly described. For instance, there is mention of reweighing based on priors, but this is not further explained. The method is not very complex, but nevertheless it would be good to be explicit about these things. Moreover, the depth of the number of wildtype sequences needed for training is not clearly stated in the main text. It is crucial to know if other people want to apply the method to larger regions in the genome. (The author mentioned they used 5847 full length coverage of the wildtype but no discussion/ analysis was provided on whether this can be lower or what the lower limit is.)
- While the regressions are done strand specific, no analysis is done on e.g. strand bias, while it is known to be very important for accurate consensus calling. Are the results indeed different for the different strands?
- Have the authors considered taking into account the observed neighbour correlation (Fig S2) in the prediction (i.e. by taking more than 1 feature into account in the logistic regression).
- It would make the work more valuable if more discussion around the potential applications of this method is provided. It is mentioned in the introduction Line: 72 that it can be used in an evolution experiment. The case demonstrated is ePCR introduced mutations on a single gene (KlenTaq Polymerase gene). Can the authors clarify the link between the two, orr link to other clinical applications such as tumor gene sequencing?

Minor feedback:

- Some figures can be enlarged. (e.g. Fig 3B).

- The manuscript is submitted as a technical note type of paper. According to the reporting standards, the manuscript should contain a section on data availability and testing to enable readers to easily reproduce the results. However, the access to data is not clearly described in the main text. Testing procedures are also not provided in github as far as this reviewer can deduce.
- Typo:Fig 3B xlabel: diference
- Typo: Line 266: "f"igure should be capital
- Clarify Availability, Operating system (If it is a R package, I expected it to be cross platform. Or please report precise operation system (e.g. x86_64-pc-linux-gnu (64-bit)), Programming language: R (version x.x.x), Other requirements (missing). The instructions to use the program is minimal on github. Consider copying the vignettes to Github README:
https://github.com/rocioespci/single/blob/master/vignettes/single.Rmd

Level of Interest

Please indicate how interesting you found the manuscript: Choose an item.

Quality of Written English

Please indicate the quality of language in the manuscript: Choose an item.

Declaration of Competing Interests

Please complete a declaration of competing interests, considering the following questions:

- Have you in the past five years received reimbursements, fees, funding, or salary from an organisation that may in any way gain or lose financially from the publication of this manuscript, either now or in the future?
- Do you hold any stocks or shares in an organisation that may in any way gain or lose financially from the publication of this manuscript, either now or in the future?
- Do you hold or are you currently applying for any patents relating to the content of the manuscript?
- Have you received reimbursements, fees, funding, or salary from an organization that holds or has applied for patents relating to the content of the manuscript?
- Do you have any other financial competing interests?
- Do you have any non-financial competing interests in relation to this paper?

If you can answer no to all of the above, write 'I declare that I have no competing interests' below. If your reply is yes to any, please give details below.

I declare that I have no competing interests

I agree to the open peer review policy of the journal. I understand that my name will be included on my report to the authors and, if the manuscript is accepted for publication, my named report including any attachments I upload will be posted on the website along with the authors' responses. I agree for my report to be made available under an Open Access Creative Commons CC-BY license (http://creativecommons.org/licenses/by/4.0/). I understand that any comments which I do not wish to be included in my named report can be included as confidential comments to the editors, which will not be published.

Choose an item.

To further support our reviewers, we have joined with Publons, where you can gain additional credit to further highlight your hard work (see: https://publons.com/journal/530/gigascience). On publication of this paper, your review will be automatically added to Publons, you can then choose whether or not to claim your Publons credit. I understand this statement.

Yes Choose an item.
