# Supplementary material for: Accurate gene consensus at low nanopore coverage
Source: Gigascience. 2022 Nov 9;11:giac102. doi: 10.1093/gigascience/giac102 (PMC9646519; doi:10.1093/gigascience/giac102)
Supplement: giac102_Reviewer_2_Report_Original_Submission — Sebastian JÃ¼nemann -- 5/13/2022 Reviewed [file giac102_reviewer_2_report_original_submission.pdf]

Reviewer Report

Title: Accurate gene consensus at low nanopore coverage

Version: Original Submission **Date:** 5/13/2022

Reviewer name: Sebastian JÃ¼nemann

Reviewer Comments to Author:

In the manuscript submitted by Rocio et al. the authors present a new method, SINGLe, to reduce noise in nanopore sequencing data in the scope of direct evolution experiments. In essence, SINGLe is a machine learning approach utilizing logistic regression trained on reference sequences to model the probability of true variants in random mutagenesis libraries (by error-prone PCR) sequenced using Oxford nanopore technology. In the results, the authors show that SINGLe shows a better performance than other approaches (like nanopolish or racon/medaka) with respect to the discrimination between correctly and incorrectly called SNPs and the number of reads necessary for building a correct consensus sequence of one variant. In summary, the submitted manuscript is a technical note that is relatively long but incomplete in terms of details on methodology and execution. Consequently, I have several major issues that will be listed in the following.

1) The structure of the manuscript could be enhance from my point of perspective in several ways. First, the authors are not clearly separating descriptive methodology from the presented results. For instance, details about the applied library preparation, sequencing, and analysis protocols and pipelines are somewhat distributed over several sections, making it hard to follow the authors without hopping through the manuscript. Also, the manuscript has in essence just two main sections, i.e., Findings and Methods. However, the Findings section contains also a Method subsection but at the same time also contains a Background subsection, which is clearly not a finding.

2) The writing could be enhanced. There are quite a lot of occasions of grammar errors or misspellings.

3; line 81 ff.) It is not clear what the data basis for the following observations are. Even though the overall statement of the correlation of quality scores and errors and the fact that nanopore errors are not evenly distributed is not to be questioned, form a strict scientific point of view it should be made clear how this was observed, precisely on what data.

4; line 82 f. and Figure 1A) As pointed out in the manuscript, the q-scores are directly related to the error rate, i.e. a low q-score indicates an erroneous base call. However, (the reference to) Figure 1A does not make this so obvious as is sounds in the text, as the figure is not normalized with respect to total counts. In fact, the total numbers of correct/false base calls is irrelevant to make this point, it is more about the reported error rate (the q-scores) vs the observed error rate (also in terms of q-scores). Something like a qq-plot would be more appropriate to make this point.

5; line 96 ff. and line 110) Where were the 6000 KlenTaq reference sequences obtained from? The later Methods section only details about the small and large variant library, not about the wild type library. Is there any kind of PCR involved here, and if so, how do the authors separate between sequencing errors or other errors sources (because, for instance, an error introduced during PCR when amplifying a specific gene is - if sequenced correctly - not a sequencing error even if the generated read mismatches

with the reference)?
6; line 105 and ff.) The abbreviation ePCR for error-prone PCR is very unusual. To the best of my knowledge, epPCR is the established short term for error-prone PCR and I would advise in using it instead of ePCR.
7; line 106 ff.) The two sentences beginning with 'In this range …' are difficult to read and should be revised. Also, the sequencer does not report a mismatch nor a wild type or a mutation, it just reports a nucleotide that can either be correct or not in comparison to a specified reference.
8; line 123 ff.) What type of logistic regression was used, what is the underlying distribution? How was the model evaluated, for goodness of fit (over- underfit)?
9; line 130 ff.) How well does the 'estimation of the mutation frequency' of the ePCR that the authors possess reflect the actual mutation rate (see also later comments)? Is there other literature on that issue?
10; line 131 ff.) How is the a priori sequencing error rate defined and what is it based on? And how was the re-weighting of the wild type fit carried out in detail?
11; line 137 ff.) In this paragraph, the authors explain how they have inferred q-scores for nucleotide positions that were deleted in the mutant type read set. First of all, what does 'fixed to the minimum Qscores of their nearest neighbors' mean precisely? To the minimum of the direct two 5' and 3' neighboring nucleotides or over a window? Second, while there is indeed an obvious correlation of q-scores of neighboring nucleotides in a sequencing read, in my opinion this is for this application an oversimplification, as it is biased towards correctly called bases (which clearly make up the majority). As the authors have already pointed out, erroneous base calls tend to have lower quality scores and they do occur all over the read. Then, how well do q-scores of these erroneous positions correlate with their neighbors? A deletion event introduced during sequencing is also an error and should be in line with or within range of q-scores of erroneous base calls. And why were insertions ignored altogether?
12; line 173) How many variants were sequenced exactly? This should be know as each library was tagged with an individual barcode.
13; line 189 ff.) If the threshold is zero, i.e. the cutoff on p_right and p_guppy is zero, signal is defined as mismatches of know mutations, no matter the of actual q-score or p_right, and noise as mismatches of non-mutated positions. The actual nucleotide sequence is equal for both cases. How can it be that the signal to noise ratio is different for guppy and SINGLe?
14; line 191 ff.) How were the counts weighted by p_right exactly. In this regard, the signal to noise ration seems to be equal despite weighting being applied or not (comparison of Fig2 and supplementary Fig3), so what is the actual methodological difference here?
15; line 194 ff.) This paragraph is somewhat imprecise and lacks scientific references on how consensus calling and polishing of nanopore reads is done on a broad scale. For instance, nanopolish uses the raw signals of the nanopore sequencer (fast5) in combination with HMMs to detect base variations, which are then filtered and eventually combined.
16; line 197 ff.) As far I understood SINGLe, it essentially transforms the q-scores into a probability score. But how is that now incorporated into a consensus calling process? All the methodological details on this are missing in the manuscript. This also applies to the p_guppy and 'no weights' consensus pipeline. And what is the difference between p_guppy and 'no weights'?
17; line 203 ff.) The consensus calling was done for each of the seven variants independently, so seven

times 47 times 50 sets. I would recommend to be precise here in the manuscript.
18; line 208 ff. and Fig 2B) Why did nanopolish failed on the #1 dataset? Given the results on the other variants this seems to be highly unlikely.
19; line 223 ff. And Fig 2D) Based on the figure it can not be concluded that nanopolish misses mutations instead of reporting false positives, as false negatives are not considered or measured in this manuscript. This even more highlights that sensitivity (recall) is an important aspect, especially in the field of variant detection. So what is the false negative rate or the sensitivity? The whole evaluation is more about precision, but precision often comes at the price of sensitivity and finding low abundant / rare variants is of high interest for many applications. Why not defining TP=mismatches of know mutations above p_right, FP=incorrect mismatches above p_right, TN=incorrect mismatches below p_right, and FN=mismatches of known mutation below p_right and presenting also incorporating evaluation statistics like F1 scores?
20; paragraph line 242) The way the authors have compared the results of the large library with that of the seven known variants is, at its best, only partially confirming the results of the latter one. First, why was medaka favored over nanopolish if nanopolish showed clearly the better performance on the seven variants dataset? Given the related results in the supplement, nanopolish failed this time on the majority of the shown datasets. Why? What are these variants and how many actual mutations do they have, is there a pattern? Second and above all, the way the ground truth is calculated is unclear. Was this done for each of the barcodes sets or over all reads? If the former was the case, how does it comes that raw reads have an inferior performance (see supplemental Figure S6). If the latter was the case, a consensus over all of the 1200 variants would most probably be skewed towards the most present variants or towards the wild type, given the fact that epPCR should introduce errors evenly and at a random rate. In this sense, this read based consensus ground truth is biased towards high precision but low recall if the consensus ground truth harbors less mutations than the actual ground truth, as this would result in not calling a mutation (high precision but low recall) being better than calling a wrong mutation (low precision but high recall). Finally, in this case SINGLe does not converge after 5 reads but after 10 reads, as there is a significant drop right after 5 reads.
21; line 253 ff. and Fig 3B) This is hard to read and understand, as the figure shows no percentages but they are discussed likewise in the manuscript. Also, the figure legend is missing essential information (e.g. what the color coding exactly means).
22; line 264) I tend to disagree, but SINGLe does not correct systematic errors. It may reduce systematic errors, but it does not, as far as it is described, specifically correct (newly) known systematic errors, as otherwise the type and nature of the systematic error along with the appropriate measurements would have been presented.
23; line 266 ff.) What is the exact data basis here? How many datasets are below five, between 6 and ten, and above ten read counts, and how are they distributed?
24; line 311 ff.) SINGLe needs optimally the true reference sequence and the expected mutation rate, which both could also be obtained by other means, e.g. using high accuracy short read sequencing, as the authors have pointed out. However, this is in contradiction to the highlighted advantages of this approach presented here, i.e., low laboratory effort at high sequencing throughput. Have such applications being conceived or carried out to give a better understanding of the practicability of using SINGLe for such cases?

25) If I understand the methods correct, the same epPCR protocoll was applied to the small and large library, but with a different cycle number (25 vs. 30), resulting in different mutation rates (3 muts/kb for the small and 8muts/kb for the large library). Why was a different rate chosen to begin with? Supplemental Table S2 shows the actual variants of the small library. Here, the first variant only shows two substitutions, which corresponds to a rate of 1.2muts/kb, whereas on the high end the variants show 4.7muts/kb. Is this the expected deviation (here about 60%), and if so, does this also apply to the large library, and if so, how well is this captured in the different datasets of the large library? How well do the different tools perform for the different types of variants (in terms of number of mutations but also substitutions versus indels)? This is important, as epPCR also have shown to be biased to some extent, favoring specific mutations over others, so introducing systematic errors of its own (see, for instance, Vanhercke et. al 2005).
26) What was the decision of the authors to only include nanopolish racon/medaka for their evaluation? These tools have originally been developed to correct genome assemblies based on signal or read information, with nanopolish being considered as almost outdated by now. Other methods have been proposed to specifically correct for systematic errors in nanopore data (e.g. Homopolish, Nextpolish, NanoReviser). Why those newer tools have not been included for the evaluation?
Figures:
27; Figure1B) It should be made clear that only a specific window of the whole gene was selected here (in contrast to the supplement).
28; Figure1C/D) The legend states that a black dot is the proportion of correct reads against the q-scores. A sequencing read does have as many q-scores as it has nucleotide positions. How was this averaged or is a black dot actually the proportion over a single base position?
29; Figure 2 and 3) The legends are too long. A legend should not contain any interpretation of the shown data but only all information necessary to fully understand the figure.
30; Supplementary Table S1) This is not a table.
31; all Figures) I would suggest to provide higher resolution graphics. Overall, the depicted information is hard to grasp visually. I would rather separate some of the sub-graphics into individual figures but make them larger in size instead.

Level of Interest

Please indicate how interesting you found the manuscript: Choose an item.

Quality of Written English

Please indicate the quality of language in the manuscript: Choose an item.

Declaration of Competing Interests

Please complete a declaration of competing interests, considering the following questions:

- Have you in the past five years received reimbursements, fees, funding, or salary from an organisation that may in any way gain or lose financially from the publication of this manuscript, either now or in the future?
- Do you hold any stocks or shares in an organisation that may in any way gain or lose financially from the publication of this manuscript, either now or in the future?
- Do you hold or are you currently applying for any patents relating to the content of the manuscript?
- Have you received reimbursements, fees, funding, or salary from an organization that holds or has applied for patents relating to the content of the manuscript?
- Do you have any other financial competing interests?
- Do you have any non-financial competing interests in relation to this paper?

If you can answer no to all of the above, write 'I declare that I have no competing interests' below. If your reply is yes to any, please give details below.

I declare that I have no competing interests.

I agree to the open peer review policy of the journal. I understand that my name will be included on my report to the authors and, if the manuscript is accepted for publication, my named report including any attachments I upload will be posted on the website along with the authors' responses. I agree for my report to be made available under an Open Access Creative Commons CC-BY license (http://creativecommons.org/licenses/by/4.0/). I understand that any comments which I do not wish to be included in my named report can be included as confidential comments to the editors, which will not be published.

Choose an item.

To further support our reviewers, we have joined with Publons, where you can gain additional credit to further highlight your hard work (see: https://publons.com/journal/530/gigascience). On publication of this paper, your review will be automatically added to Publons, you can then choose whether or not to claim your Publons credit. I understand this statement.

Yes Choose an item.
